# 1-*O*-Octadecyl-2-*O*-benzyl-*sn*-glyceryl-3-*phospho*-GS-441524 (V2043). Evaluation of Oral V2043 in a Mouse Model of SARS-CoV-2 Infection and Synthesis and Antiviral Evaluation of Additional Phospholipid Esters with Enhanced Anti-SARS-CoV-2 Activity

**DOI:** 10.1021/acs.jmedchem.3c00046

**Published:** 2023-04-11

**Authors:** Aaron F. Carlin, James R. Beadle, Alex E. Clark, Kendra L. Gully, Fernando R. Moreira, Ralph S. Baric, Rachel L. Graham, Nadejda Valiaeva, Sandra L. Leibel, William Bray, Rachel E. McMillan, Jonathan E. Freshman, Aaron F. Garretson, Rachael N. McVicar, Tariq Rana, Xing-Quan Zhang, Joyce A. Murphy, Robert T. Schooley, Karl Y. Hostetler

**Affiliations:** † Department of Medicine, University of California, San Diego, La Jolla, California 92093, United States; ‡ Department of Pathology, University of California, San Diego, La Jolla, California 92093, United States; § Department of Epidemiology, Gillings School of Global Public Health, University of North Carolina at Chapel Hill, Chapel Hill, North Carolina 27599, United States; ∥ Department of Pediatrics, University of California, San Diego, La Jolla, California 92093, United States; ⊥ Sanford Burnham Prebys Discovery Institute, La Jolla, California 92037, United States

## Abstract

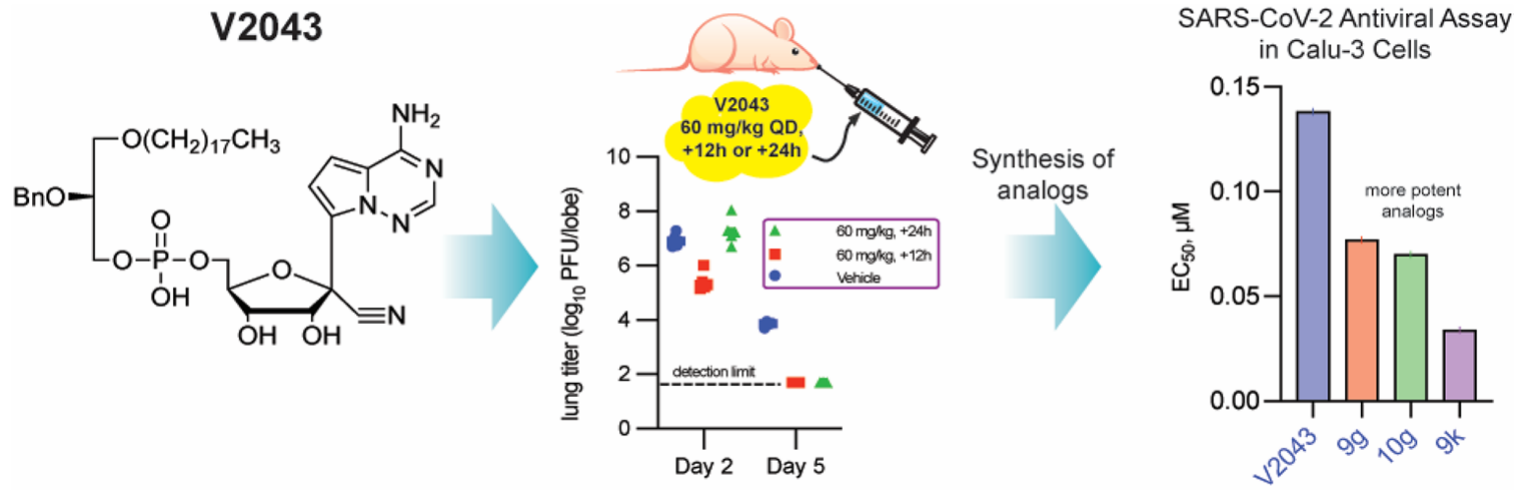

Early antiviral treatments, including intravenous remdesivir (RDV), reduce hospitalization and severe disease caused by COVID-19. An orally bioavailable RDV analog may facilitate earlier treatment of non-hospitalized COVID-19 patients. Here we describe the synthesis and evaluation of alkyl glyceryl ether phosphodiesters of GS-441524 (RVn), lysophospholipid analogs which allow for oral bioavailability and stability in plasma. Oral treatment of SARS-CoV-2-infected BALB/c mice with 1-*O*-octadecyl-2-*O*-benzyl-*sn*-glyceryl-3-*phospho*-RVn (60 mg/kg orally, once daily for 5 days starting 12h after infection) reduced lung viral load by 1.5 log_10_ units versus vehicle at day 2 and to below the limit of detection at day 5. Structure/activity evaluation of additional analogs that have hydrophobic ethers at the *sn*-2 of glycerol revealed improved *in vitro* antiviral activity by introduction of a 3-fluoro-4-methoxy-substituted benzyl or a 3- or 4-cyano-substituted benzyl. Collectively, our data support the development of RVn phospholipid prodrugs as oral antiviral agents for prevention and treatment of SARS-CoV-2 infections.

## Introduction

Since its emergence in late 2019, SARS-CoV-2 has resulted in the deaths of more than a million people in the United States and over 6.5 million people globally.^1^ Despite the dramatic impact of SARS-CoV-2 vaccines on COVID-19-related morbidity, COVID-19 remains the third most frequent cause of death in the U.S. behind only cardiovascular disease and cancer.^2^ The emergence of novel SARS-CoV-2 variants coupled with the natural decay of coronavirus immunity has contributed to successive waves of new and breakthrough infections since the advent of COVID-19 vaccines. Vaccine hesitancy and the partial vaccine protection provided to older and immunocompromised populations further compromise vaccine efficacy at the population level.^3−5^ Viral evolution has also severely compromised the efficacy of all currently available SARS-CoV-2-specific monoclonal antibodies for the prevention or treatment of the infection.^6^

Two orally bioavailable antiviral drugs are currently available, nirmatrelvir/ritonavir and molnupiravir.^7,8^ Nirmatrelvir/ritonavir reduces hospitalization and death, but the substantial number of ritonavir-related drug–drug interactions related to its effects on hepatic metabolism and gut transport mechanisms, as well as the physician reticence to dose-adjust other medications in patients who are otherwise stable, complicate its administration to a substantial fraction of patients.^9^ This is particularly problematic in immunocompromised and older populations who frequently require drugs that are contraindicated in persons receiving ritonavir.^9^ Molnupiravir is less affected by drug–drug interactions but is substantially less effective than nirmatrelvir/ritonavir in reducing morbidity.^8^ Viral variants with substantially lower susceptibility to nirmatrelvir/ritonavir have been selected *in vitro*.^10−12^ Collectively, these factors promise ongoing COVID-19-related morbidity and argue strongly for additional investments in the development of orally bioavailable antiviral agents. Our goal is to develop an orally bioavailable drug that is as clinically effective as nirmatrelvir/ritonavir and that can be administered to patients on complex drug regimens without major concerns about drug–drug interactions. Several oral analogs of RDV, including GS-621763, ATV006, and VV116, have demonstrated beneficial antiviral effects after oral administration in murine model systems.^13−15^ VV116 is a deuterated remdesivir hydrobromide that has recently demonstrated an impact of sustained clinical recovery from COVID-19 in humans that is non-inferior to that of nirmatrelvir/ritonavir.^16^

**Figure 1:**
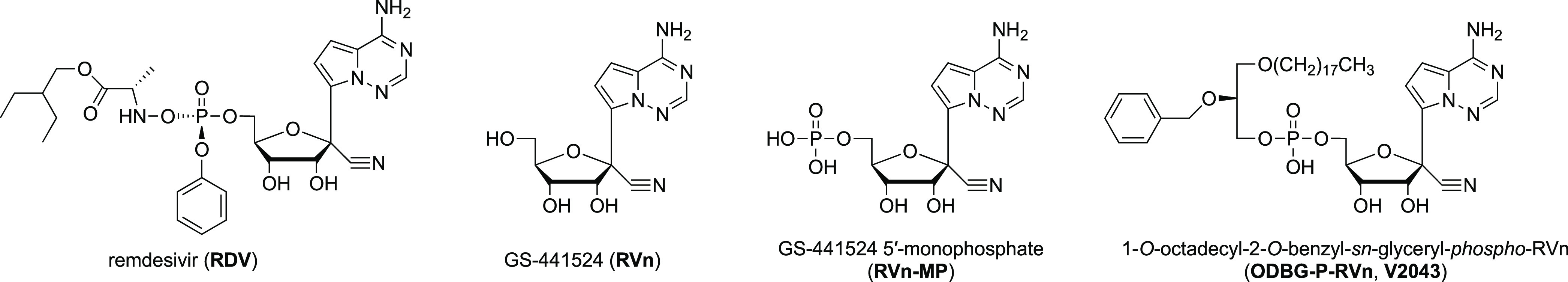
**Structures of remdesivir (RDV), its metabolites (RVn, RVn-MP), and V2043, an orally available RVn phospholipid prodrug.**

Remdesivir (RDV) reduces related morbidity and mortality from SARS-CoV-2, but its use requires intravenous administration.^17^ Administration to outpatients shortly after infection demonstrated substantial promise, but scalability of this approach is challenging since it requires six separate outpatient infusions over a three-day period.^18^ Availability of an orally available RDV prodrug might facilitate intervention early after infection, further reducing hospitalization and mortality from COVID-19. RDV is an aryloxy phosphoramidate prodrug that is converted by a series of reactions to GS-441524 (RVn), GS-441524 5′-monophosphate (RVn-MP), and RVn-triphosphate, the antiviral nucleotide substrate (Figure 1). Efficient, selective incorporation of RVn-triphosphate by viral RNA-dependent RNA polymerases leads to its potent inhibitory activity against positive-sense RNA viruses, including coronaviruses.^19^

During previous efforts to identify orally active antiviral prodrugs, we esterified various poorly absorbed nucleoside and nucleotide analogs with alkoxyalkyl and other phospholipid-like groups, creating analogs that resemble lysophospholipids.^20,21^ This modification facilitates absorption in the gastrointestinal tract, delivering the intact prodrug to the systemic circulation and efficiently loading target cells with active triphosphate (or diphosphate) metabolites, and may improve delivery to the lungs.^22^ The application of this strategy resulted in Tembexa (brincidofovir), a recently FDA-approved oral pill for smallpox.^23^

Applying this approach to prepare oral RDV analogs, we synthesized 1-*O*-octadecyl-2-*O*-benzyl-*sn*-glyceryl-*phospho*-RVn (ODBG-P-RVn, V2043, Figure 1) and showed that it inhibits SARS-CoV-2 replication in Huh7.5 cells (EC_50_ = 138 nM) and is orally bioavailable in Syrian hamsters.^24^ In this study we assessed the antiviral effect of V2043 in mice infected with SARS-CoV-2. For structure/activity assessment we investigated the effects of varying the long alkyl group at the *sn*-1 hydroxyl and making various substitutions at the *sn*-2 hydroxyl of the glyceryl phosphodiester on antiviral activity and cytotoxicity. Additional 1-*O*-alkyl-2-*O*-substituted glyceryl analogs were synthesized, including various alkyl, cycloalkyl, and substituted benzyl groups, and their anti-coronavirus activities and selectivities were assessed *in vitro*.

## Results and Discussion

### Oral V2043 Therapeutically Protects Mice Infected with SARS-CoV-2

**Figure 2:**
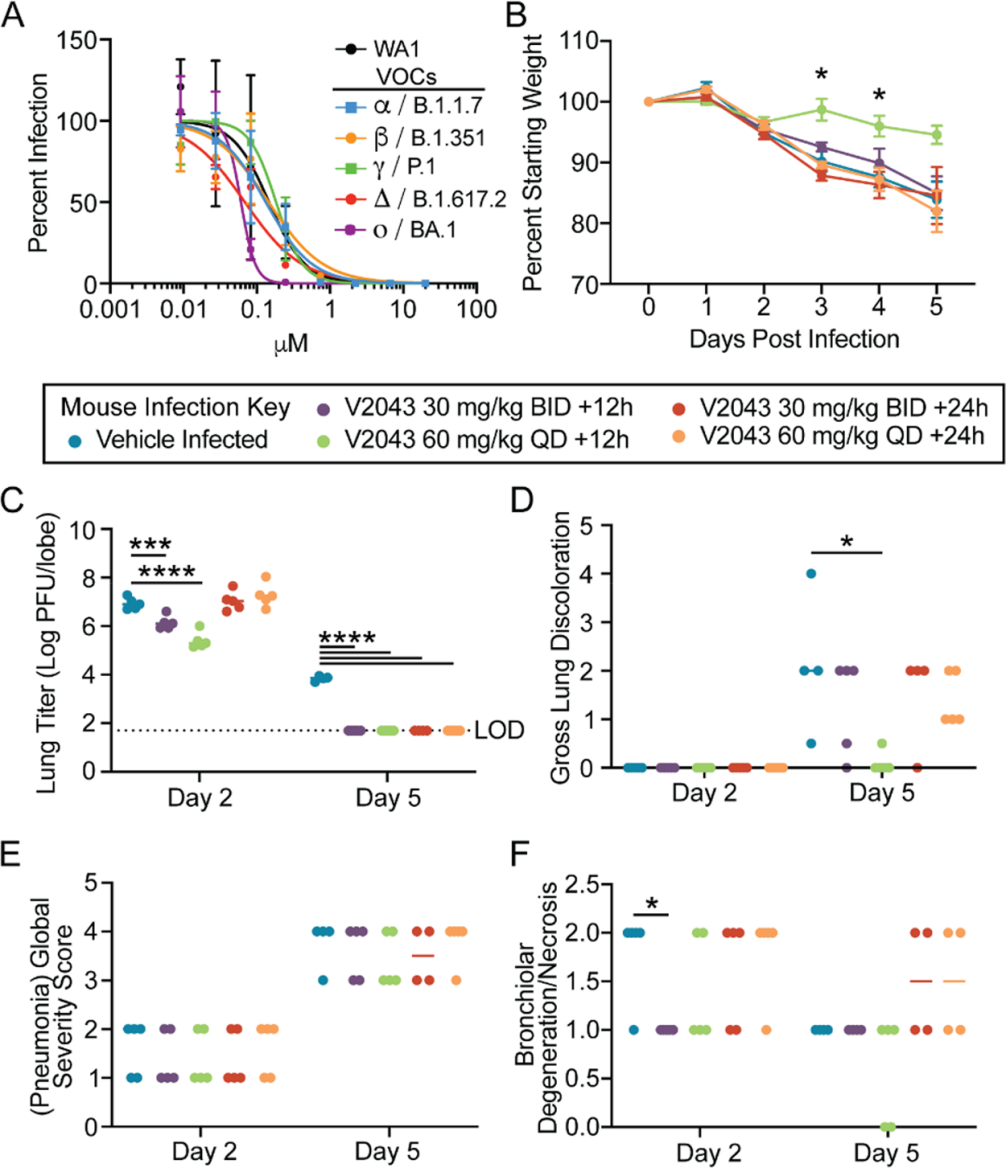
**Therapeutic oral administration of V2043 protects young BALB/c mice from SARS-CoV-2 infection.** (A) Average dose–response inhibition of authentic SARS-CoV-2 isolates, including variants of concern, by V2043 in Calu-3 cells. (B–F) Mice were infected with 1 × 10^4^ PFU SARS-CoV-2 MA10 and treated therapeutically with vehicle or 30 mg/kg V2043 or 60 mg/kg V2043 at 12h or 24h post-infection. (B) Relative daily weight of mice in each group (*n* = 10 per total group, *n* = 5 per group per experimental endpoint). (C) Lung viral titers were measured in mice that were therapeutically treated with vehicle or V2043 at the indicated dosing schedule (*n* = 10 per total group, *n* = 5 per group per experimental endpoint). The dashed line indicates the limit of detection (LoD). (D) Day 5 gross lung congestion scores were calculated in mice therapeutically treated with vehicle or V2043 (*n* = 5 per group). (E, F) Pneumonia Global Severity Score (E) and Bronchiolar Degeneration/Necrosis (F) were scored in a blinded manner. Data were analyzed using a two-way ANOVA (weight loss) or a Kruskal–Wallis test (lung titer, gross lung discoloration, and pathology scores), *P < 0.05, **P < 0.005, ***P < 0.0005, and ****P < 0.0001. Data in (B) and (C) are shown as means ± SEM. Data in (D), (E), and (F) are shown as medians.

We previously showed that V2043 is a lipid prodrug of RVn with submicromolar antiviral activity in a variety of cell types when tested against an early SARS-CoV-2 isolate, WA1 (USA-WA1/2020, lineage A, oral bioavailability, stability in plasma and bypass of the first phosphorylation step).^24^ Given the continued emergence of new variants, we infected Calu-3 cells, a human lung epithelial cell line, with SARS-CoV-2 variants of concern (VOCs) and found no significant difference in the half-maximal effective concentration (EC_50_) of V2043 (Figure 2A, Table 1).

To evaluate the capacity of orally administered V2043 to block viral replication and prevent severe disease in a mouse model of SARS-CoV-2 infection, we infected young (∼10- to 12-week-old) BALB/c mice with 10^4^ PFU of sequence- and titer-verified SARS-CoV-2 MA10.^25,26^ V2043 was administered PO at two dosages (30 mg/kg and 60 mg/kg) and two dosing schedules (QD and BID) starting 12h or 24h post-infection (+12h or +24h). Outcomes were assessed as follows: 1) weight loss post-viral infection, 2) viral titers at the terminal endpoints, 3) gross lung discoloration at the terminal endpoints post-infection, and 4) histopathological scoring of lung samples. Mice administered V2043 at the 60 mg/kg dose and QD +12h dosing schedule showed significant protection from weight loss on days 3 and 4 post-infection and nearly significant protection from weight loss on day 5 post-infection (Figure 2B). No other dosing/schedule groups showed statistical protection from weight loss upon infection. Treatment with V2043 (60 mg/kg QD +12h) reduced lung titers 1.52 log_10_ at day 2 post-infection, and all V2043 (+12h or +24h) treatment groups reduced lung titers >2.14 log_10_ at day 5 post-infection compared to placebo. Given that none of the animals in any V2043 treatment group had detectable virus on day 5, the antiviral efficacy is likely greater than 2.14 log_10_.

In comparing our results with published antiviral data, variations in the design of animal studies do not allow us to directly compare the potency of V2043 with other published results. The most closely related study is the study published by Schäfer et al.^13^ Using the same BALB/c mouse model infected with 10^4^ PFU SARS-CoV-2 strain MA10, they compared the relative efficacy of molnupiravir (30 mg/kg BID or 60 mg/kg BID) or GS-621763 (an oral prodrug of remdesivir nucleoside GS-441524) administered 12h or 24h post-infection. The molnupiravir 60 mg/kg BID dose results in similar exposures to those observed in humans receiving 800 mg BID, the FDA EUA-approved dose for humans with COVID-19. Schäfer et al. measured lung titers at only one time point, day 4 post-infection. At day 4 post-infection, the following reductions in lung virus titers were demonstrated: molnupiravir 30 mg/kg BID +12h = 2.1 log and 60 mg/kg BID +12h = 2.9 log_10_, molnupiravir 60 mg/kg BID +24h reduced = 2.6 log_10_, GS-621763 30 mg/kg BID +12h > 2.9 log_10_, and GS-621763 60 mg/kg BID +24h >2.9 log_10_. Although these results cannot be directly compared with our data (day 4 vs day 5), V2043 efficacy appears to be similar. However, on a molar basis, V2043 is substantially more active than molnupiravir and similar to GS-621763 (see Table S1 in the Supporting Information).

Mice that were treated with V2043 at the 60 mg/kg dose and QD +12h group also showed significantly lower gross lung congestion score on day 5 post-infection (Figure 2D). Histological specimens were graded for vasculitis/endotheliitis, type II pneumocyte hyperplasia, bronchiolar degeneration/necrosis, bronchiolar hyperplasia, bronchointerstitial pneumonia, and global pneumonia severity scores by a board-certified veterinary pathologist (Figure 2E,F). Compared to the vehicle-treated infected control, V2043 treatment resulted in no significant decreases in global pneumonia severity, bronchointerstitial pneumonia, type II pneumocyte hyperplasia, bronchiolar hyperplasia, or vasculitis/endotheliitis (Figure 2E). There were no significant differences in type II pneumocyte hyperplasia and vasculitis/endotheliitis. There was a significant difference in bronchiolar degeneration/necrosis in the 30 mg/kg dosing BID +12h group (day 2 but not day 5) (Figure 2F). These results demonstrate that therapeutic oral administration of V2043 improves virologic and some pathologic endpoints and that the degree of improvement is dependent on time of initiation and dose frequency. Additionally, V2043 should be effective against other VOCs (Table 1).

### Synthesis of V2043 Analogs

After confirming oral bioavailability and *in vitro* efficacy of lead compound V2043,^24^ we synthesized additional analogs of RVn to see if antiviral activity and selectivity could be improved. Compounds with alterations in the long alkyl group at the *sn*-1 position of the glyceryl moiety and compounds with various hydrophobic short alkyl groups at the *sn*-2 position were prepared, and their antiviral activity and cytotoxicity were evaluated.

**Scheme 1:**
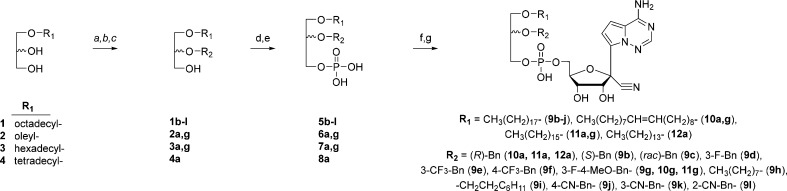
Synthesis of 1-*O*-Alkyl-2-*O*-Substituted Esters of GS-441524 5′-Monophosphate **Reagents and conditions**: (a) monomethoxytrityl chloride, DMAP, pyridine, rt, 18h; (b) benzyl or alkyl bromides, NaH, *tert*-butylammonium iodide 30 mol%, 18h; (c) p-TsOH 5 mol%, 1:1 MeOH/CHCl_3_, rt, 3h; (d) bis-trichloroethyl chlorophosphate, *N*-methylimidazole, pyridine, rt, 18h; (e) zinc, CHCl_3_, CH_3_COOH, rt, 6h; (f) diisopropylcarbodiimide (DIC, 2 equiv), *N*-methylimidazole (3 equiv), pyridine, 35 °C, 18h; (g) formic acid (88%), rt, 3h.

The new analogs were synthesized according to Scheme 1 using methods published by us and others.^20,27^ To facilitate the creation of a more diverse analog library, additional compounds were custom synthesized at J-Star Research, Inc. (South Plainfield, NJ) and Nanosyn, Inc. (Santa Clara, CA). Several 1-*O*-alkyl-*sn*-glycerols were purchased (**1**, **3**, Bachem Americas, Torrance, CA) or prepared (**2**, **4**) by alkylation of 2,3-isopropylidene glycerol with the appropriate bromoalkane or alkyl methanesulfonate.^28^ After monomethoxytrityl (or trityl) protection at the *sn*-3 hydroxyl,^29^ the *sn*-2 hydroxyl was alkylated with various alkyl, benzyl, or substituted benzyl bromides. Deprotection afforded a series of lipid glyceryl ethers which were phosphorylated by published procedures^30,28^ and then coupled to GS-441524-2′,3′-acetonide^31^ using diisopropylcarbodiimide (DIC)/*N*-methylimidazole (NMI)/pyridine. Finally, removal of the acetonide by treatment with formic acid provided target 1-*O*-alkyl-2-*O*-substituted *sn*-glyceryl-3*-phospho*-RVn. Tested compounds were characterized by ^1^H and ^13^C NMR and high-resolution mass spectroscopy (HRMS). Analysis by high-performance liquid chromatography (HPLC) showed purity greater than 95% for each tested compound except for **9g**, which was 94.7% by HPLC.

### Structural Modifications of Lipid Prodrug V2043 Improve Antiviral Activity

**Figure 3:**
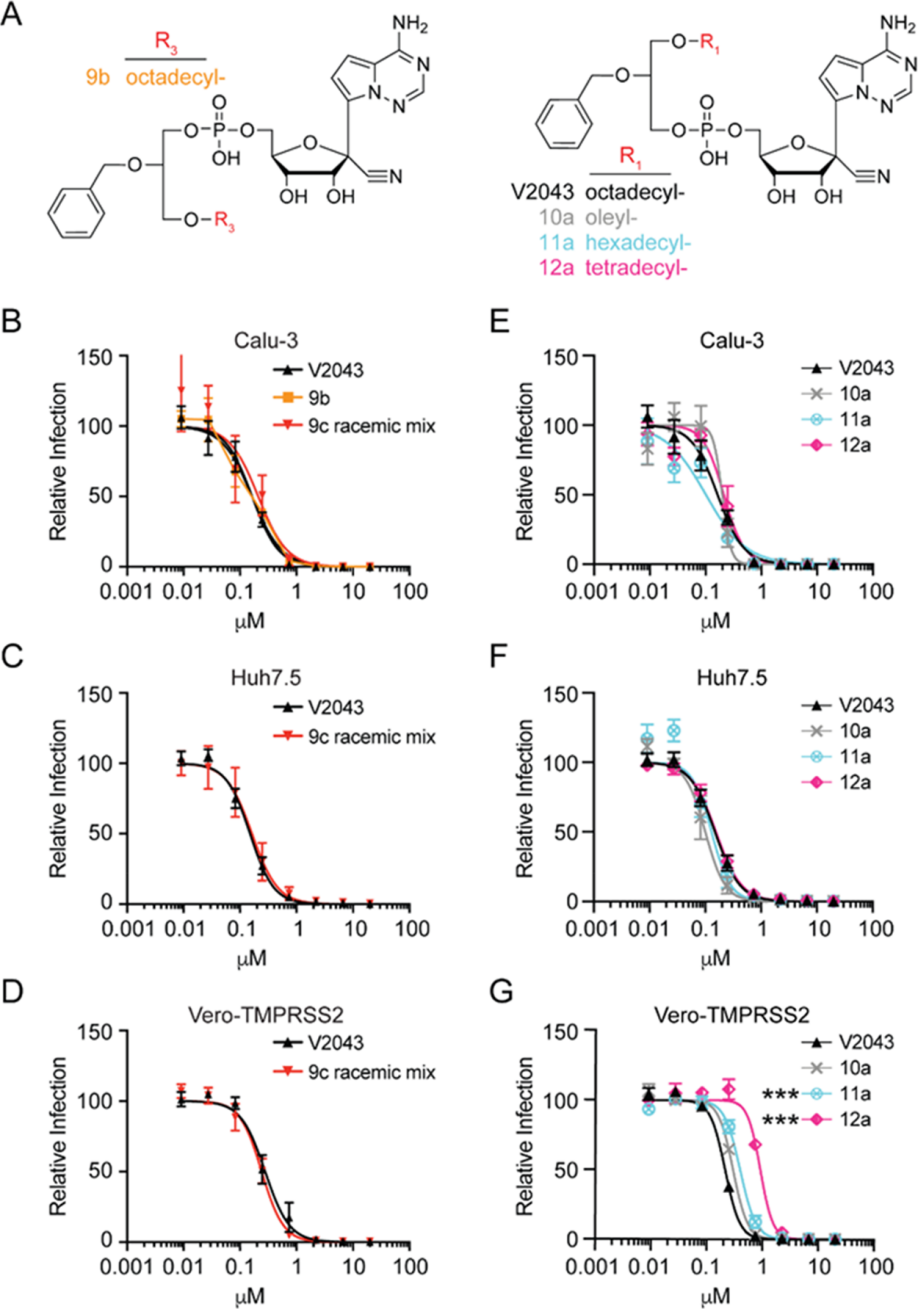
**Glycerol stereochemistry and R**_**1**_**long-chain alkyl modifications do not significantly alter the antiviral activity of 2-*O*-benzyl-*sn*-glyceryl-*phospho*-RVn.** (A) Structures of 3-*O*-octadecyl-2-*O*-benzyl-*sn*-glyceryl ester (**9b**), V2043, and R_1_-modified 2-*O*-benzyl-*sn*-glyceryl-*phospho*-RVn analogs with various R_1_ substitutions **10a**, **11a**, and **12a**. (B–G) Average dose–response inhibition of authentic SARS-CoV-2 isolate WA1 by the indicated compounds in Calu-3, Huh7.5, and Vero-TMPRSS2 cells. Relative infection was determined by immunofluorescence followed by automated counting. Data points and curves represent the mean ± SEM derived from at least three independent experiments performed in duplicate. LogEC_50_ values from each experiment were compared to results for V2043 by one-way ANOVA with Dunnett’s correction for multiple comparisons, ***P < 0.001.

We systematically evaluated the anti-SARS-CoV-2 activity of V2043 analogs in various *in vitro* models, including Calu-3, Huh7.5 (human hepatoma cell line), and VeroE6 (African green monkey kidney epithelial cell line) expressing TMPRSS2, a protease involved in SARS-CoV-2 entry (Vero-TMPRSS2). First, we determined if the configuration at the *sn*-2 chiral center affects the antiviral activity of V2043. The antiviral activities of V2043 (*R*-config), **9b** (*S*-config), and **9c** (racemic) isomers were indistinguishable in our assays (Figure 3A–D and Table 1). Hexadecyl (**11a**) or tetradecyl (**12a**) R_1_ analogs of V2043 (octadecyl) had similar EC_50_ values in Calu-3 and Huh7.5 cells but were significantly less active in Vero-TMPRSS2 cells (Figure 3E–G and Table 1).

An R_1_ oleyl analog showed anti-SARS-CoV-2 activity similar to that of V2043 (Figure 3E–G and Table 1). R_1_ long alkyl chains of 14 to 18 carbons generally provided similar antiviral activity. These data demonstrate that modifications of glycerol stereochemistry and R_1_ alkyl length or saturation do not significantly improve the *in vitro* anti-SARS-CoV-2 activity of 2-*O*-benzyl-*sn*-glyceryl-*phospho*-RVn analogs compared to V2043.

**Figure 4:**
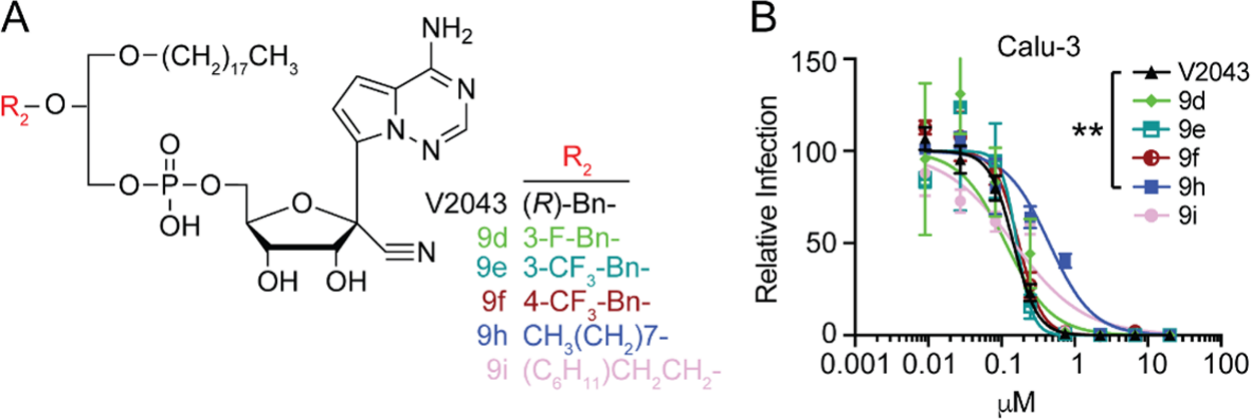
**Many hydrophobic lower alkyl modifications at R**_**2**_**are permissive but do not improve antiviral activity.** (A) Structures of R_2_-modified 1-*O*-octadecyl-*sn*-glyceryl-*phospho*-RVn analogs. (B) Average dose–response inhibition of authentic SARS-CoV-2 isolate WA1 by the indicated compounds in Calu-3 cells. Relative infection was determined by immunofluorescence followed by automated counting. Data points and curves represent the mean ± SEM derived from at least three independent experiments performed in duplicate. LogEC_50_ values from each experiment were compared to V2043 by one-way ANOVA with Dunnett’s correction for multiple comparisons, **P < 0.01.

**Figure 5:**
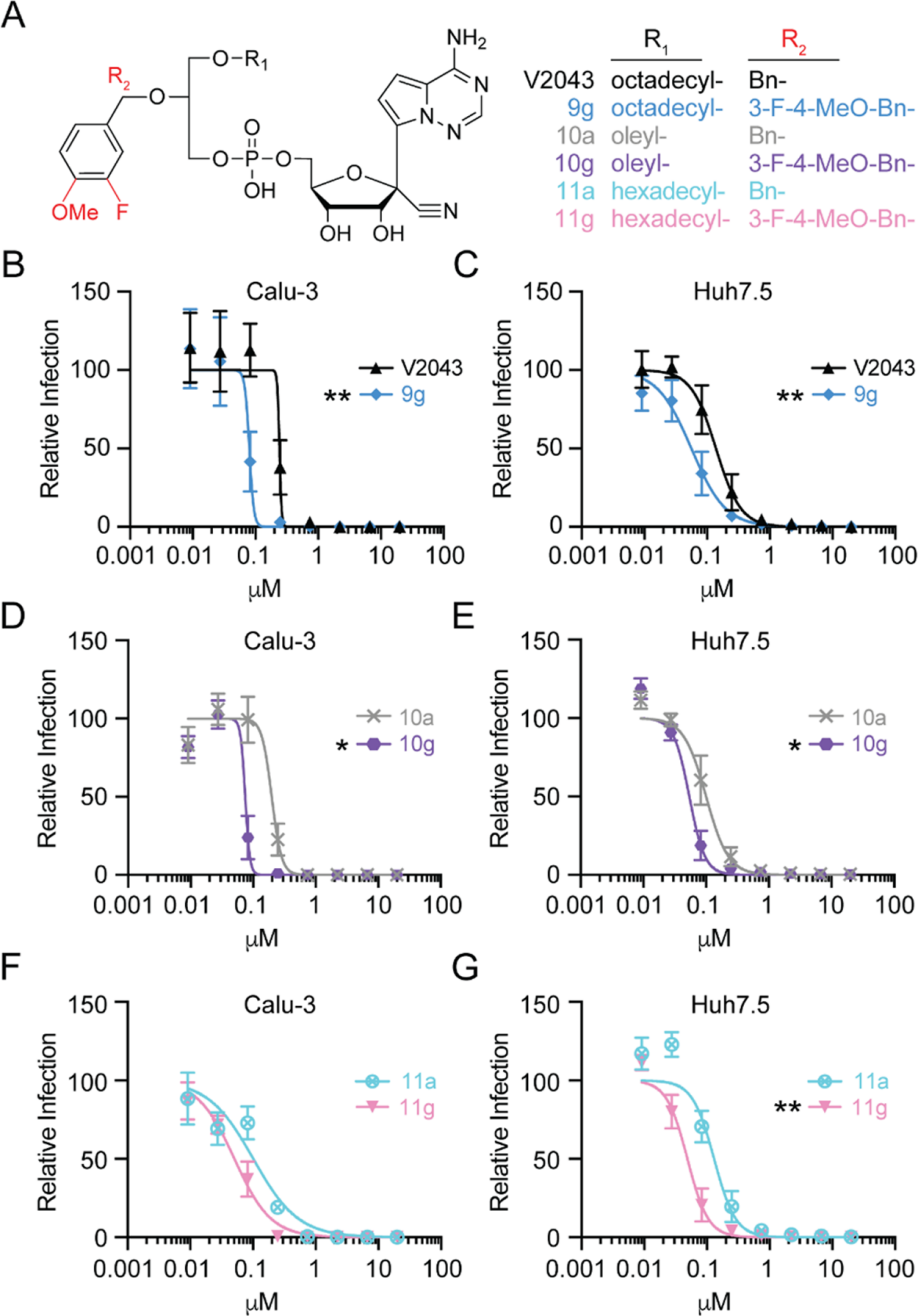
**R**_**2**_**3-F-4-MeO-benzyl substitution improves antiviral activity.** (A) Structures of paired compounds containing either Bn (V2043, **10a**, **11a**) or 3-F-4-MeO-Bn (**9g**, **10g**, **11g**) at R_2_. (B–G) Average dose–response inhibition of authentic SARS-CoV-2 isolate WA1 by the indicated compounds in Calu-3 and Huh7.5 cells. Relative infection was determined by immunofluorescence followed by automated counting. Data points and curves represent the mean ± SEM derived from at least three independent experiments performed in duplicate. LogEC_50_ values were compared by paired two-sided *t* test, *P < 0.05, **P < 0.01.

We next determined if modifying the R_2_ position of 1-octadecyl-*sn*-glyceryl-3-*phospho*-RVn could improve antiviral potency. A benzyl substitution at the R_2_ position was previously shown to increase lung exposure to a phospholipid prodrug of cidofovir.^22^ Many modifications, including 2-*O*-octyl (**9h**) or 2-*O*-ethylcyclohexyl (**9i**) substituents, or single substitutions on the 2-*O*-benzyl group, such as addition of 3-F (**9d**), 3-CF_3_ (**9e**), or 4-CF_3_ (**9f**), gave activity similar to that of V2043 but provided no improvement of inhibitory activity (Figure 4A,B). Select modifications of the R_2_ benzyl significantly improved antiviral potency. Compound **9g**, which contains a 3-F-4-MeO-substituted benzyl group, demonstrated improved anti-SARS-CoV2 activity in Calu-3 and/or Huh7.5 cells when compared to V2043 (Figures 5A,B and Table 1).

**Figure 6:**
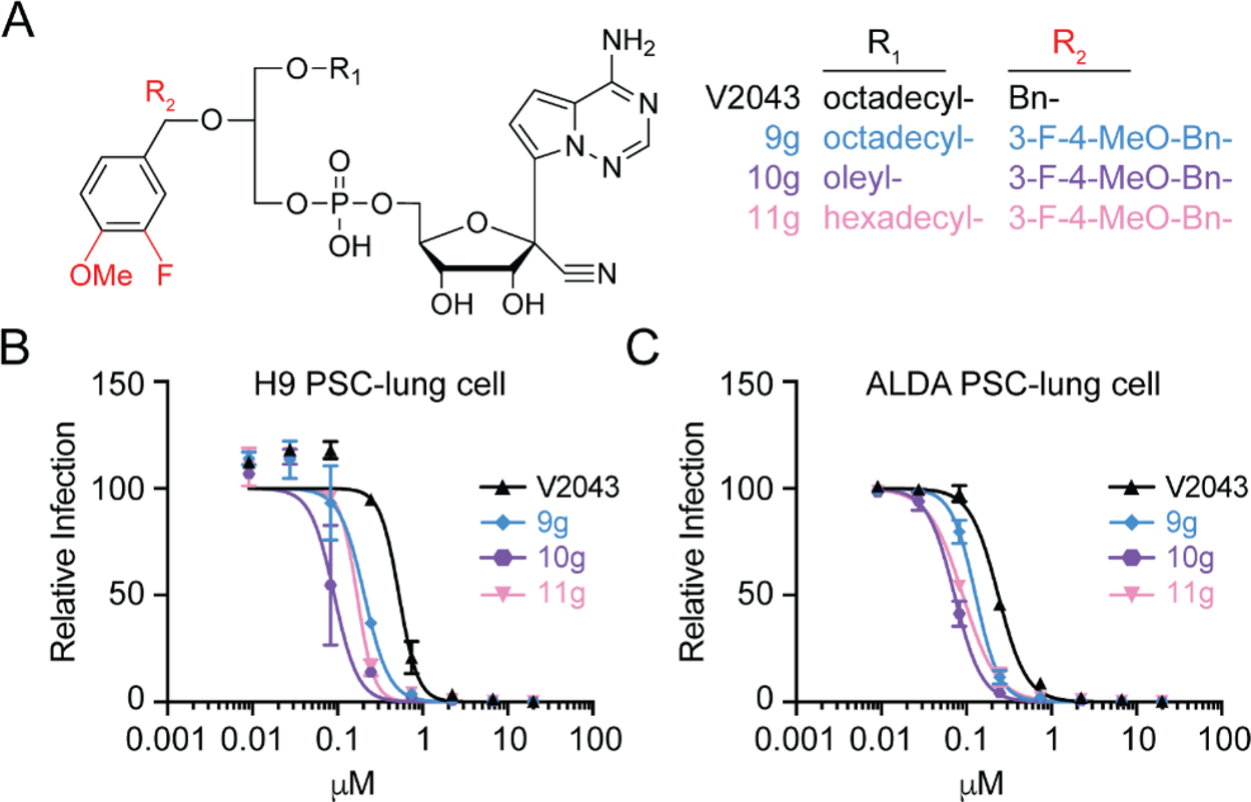
**3-F-4-MeO-Bn-substituted analogs are more potent than V2043 in human pluripotent stem cell-derived lung cells.** (A) Structures of V2043, **9g**, **10g**, and **11g**. (B, C) Average dose–response inhibition of authentic SARS-CoV-2 isolate WA1 by the indicated compounds in two different PSC-lung cells (H9 and ALDA). Relative infection was determined by immunofluorescence followed by automated counting. Data points and curves represent the mean ± standard deviation (SD) of one experiment performed in duplicate.

Additionally, compounds **10g** and **11g**, which contain 3-F-4-MeO-benzyl substitutions, had lower EC_50_ values in Calu-3 and Huh7.5 cells when compared to their corresponding unsubstituted benzyl analogs, **10a** and **11a** (Figure 5A and D–G and Table 1). The 3-F-4-MeO-benzyl-substituted compounds **9g**, **10g**, and **11g** were among the most active compounds tested, with EC_50_ (Calu-3) = 0.077, 0.070, and 0.051 μM, respectively (Table 1). We next compared the antiviral activity of V2043, **9g**, **10g**, and **11g** using two human pluripotent stem cell-derived lung cells (PSC-lung). The 3-F-4-MeO-benzyl-substituted compounds **9g**, **10g**, and **11g** were more potent in both PSC-lung cells, with average EC_50_ values of 0.167, 0.082, and 0.130 μM, respectively (Figure 6A–C and Table 1). Compared to V2043, with EC_50_ = 0.138 μM (Calu-3) and 0.382 (PSC-lung), the presence of the 3-F-4-MeO substitutions provided approximately 2–4.5-fold improved antiviral activity in these *in vitro* human lung cell models (Table 1). These results demonstrate that 3-F-4-MeO substitution of the R_2_ benzyl improves antiviral potency in multiple cell types independent of R_1_ alkyl length or saturation.

**Figure 7:**
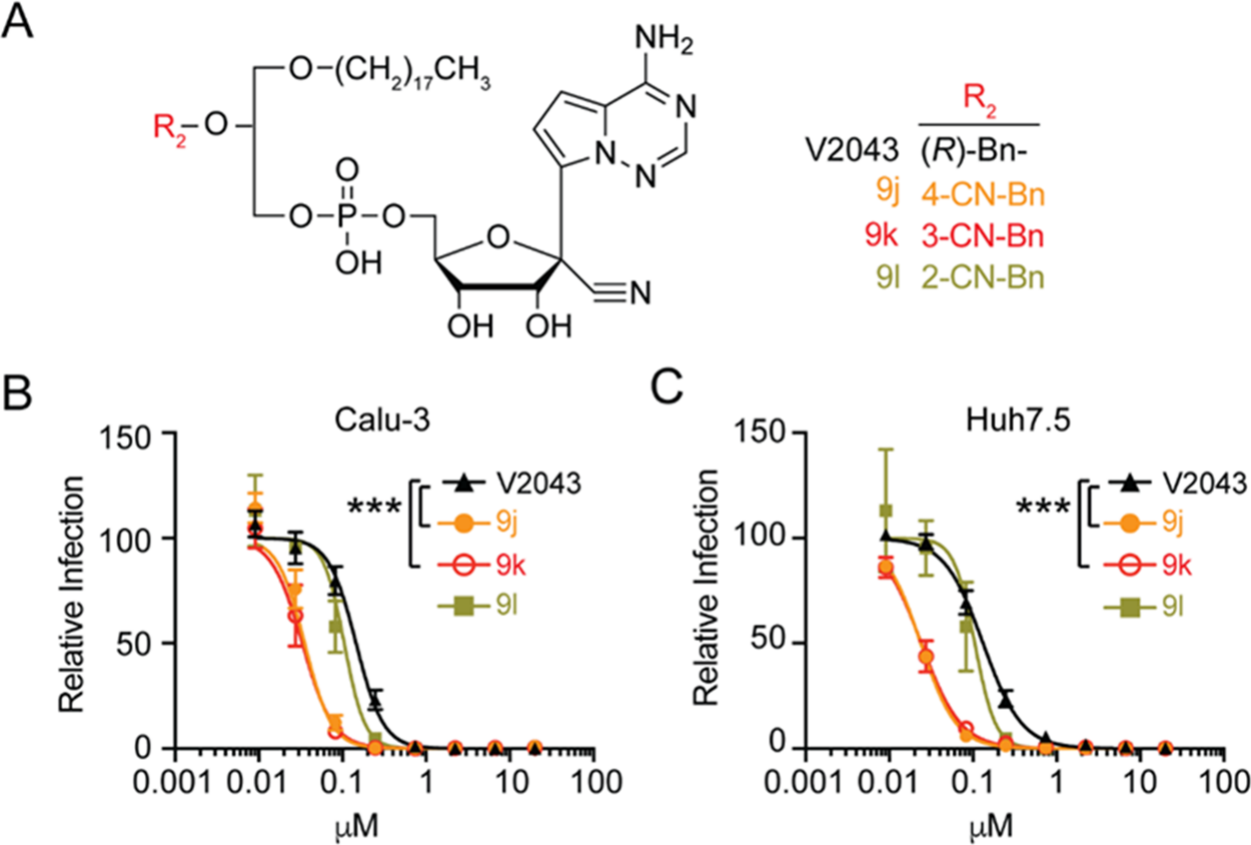
**3- or 4-cyano benzyl-substituted analogs are more potent than V2043.** (A) Structures of V2043, **9j**, **9k**, and **9l**. (B, C) Average dose–response inhibition of authentic SARS-CoV-2 isolate WA1 by the indicated compounds in Calu-3 and Huh7.5 cells. Relative infection was determined by immunofluorescence followed by automated counting. Data points and curves represent the mean ± SEM derived from at least three independent experiments performed in duplicate. LogEC_50_ values were compared by one-way ANOVA and then compared to V2043 using Dunnett’s correction for multiple comparisons, *P < 0.05, **P < 0.01.

Finally, compounds **9j** and **9k**, which contain a 4-CN or 3-CN substitution on the 2-O-benzyl, respectively, displayed similar activity and were the most potent compounds tested (Figure 7A–C and Table 1). In comparison, a 2-CN substitution on the 2-O-benzyl compound **9l** did not improve antiviral activity. Compared to V2043, the EC_50_ values of **9j** [0.043 μM Calu-3 and 0.024 μM Huh7.5] and **9k** [0.034 μM Calu-3 and 0.024 μM Huh7.5] were 3–5.5-fold lower. Collectively these data demonstrate that select modifications of 1-*O*-alkyl-2-*O*-substituted glyceryl RVn analogs can increase antiviral potency compared to the original lead compound, V2043.

The original lead compound, V2043, was designed to provide oral bioavailability and stability in plasma and to bypass the first phosphorylation of RVn.^24^ Kinase bypass occurs upon intracellular cleavage of V2043 by sphingomyelin phosphodiesterase I, generating RVn monophosphate and the glycerolipid. RDV also bypasses the first phosphorylation but is unstable in plasma and is not orally bioavailable. The antiviral activities of RDV and V2043 are generally similar based on EC_50_ values (see Table 5), but newer compounds which have favorable 2-*O*-benzyl substitutions, **9j**, **9k**, and **10g**, are respectively 5.3-, 6.7-, and 3.3-fold more active than RDV in Calu-3 cells and 11.5-, 9.1-, and 5.6-fold more active than 116-N1, the parent nucleoside of VV116.^15^

Tables 1–4 present key antiviral and cytotoxicity data calculated from Figures 2–7, showing results as the effective concentrations at 50% and 90% (EC_50_ and EC_90_) and the cytotoxic concentration at 50% (CC_50_) in micromoles/liter as mean ± standard deviation, and the selectivity index values (SI = CC_50_/EC_50_) are also reported. Tables 1–4 report the antiviral parameters in Calu-3 cells, Huh7.5 cells, Vero-TMPRESS2 cells, and human pluripotent stem cell-derived lung cells, respectively. Table 5 reports a comparison of antiviral parameters of the most active RVn prodrugs with remdesivir and RVn (GS-441524).

**Table 1:** Antiviral Activity and Cytotoxicity in Calu-3 Cells^a^

Species/Variant	Compound	R_1_	R_2_	EC_50_, μM	EC_90_, μM	CC_50_, μM	SI
WA1	V2043	octadecyl	(*R*)-Bn	0.136 ± 0.056	0.323 ± 0.157	58.30	428
	**9b**	octadecyl	(*S*)-Bn	0.160 ± 0.024	0.625 ± 0.195	93.40	583
	**9c**	octadecyl	(*R,S*)-Bn	0.172 ± 0.078	1.071 ± 1.045	92.10	535
	**9d**	octadecyl	3-F-Bn	0.224 ± 0.129	0.506 ± 0.204	94.70	422
	**9e**	octadecyl	3-CF_3_-Bn	0.160 ± 0.093	0.316 ± 0.083	66.90	418
	**9f**	octadecyl	4-CF_3_-Bn	0.177 ± 0.012	0.408 ± 0.181	90.20	509
	**9g**	octadecyl	3-F-4-MeO-Bn	0.077 ± 0.027	0.145 ± 0.054	50.50	656
	**9h**	octadecyl	octyl	0.391 ± 0.039	2.50 ± 1.236	>100	>255
	**9i**	octadecyl	ethylcyclohexyl	0.144 ± 0.020	1.828 ± 1.045	98.70	685
	**9j**	octadecyl	4-CN-Bn	0.043 ± 0.013	0.082 ± 0.022	60.20	1400
	**9k**	octadecyl	3-CN-Bn	0.034 ± 0.007	0.065 ± 0.036	61.55	1810
	**9l**	octadecyl	2-CN-Bn	0.091 ± 0.025	0.186 ± 0.047	82.8	909
	**10a**	oleyl	(*R*)-Bn	0.193 ± 0.058	0.324 ± 0.127	79.20	410
	**10g**	oleyl	3-F-4-MeO-Bn	0.070 ± 0.003	0.149 ± 0.117	58.70	839
	**11a**	hexadecyl	(*R*)-Bn	0.095 ± 0.012	0.716 ± 0.270	88.70	934
	**11g**	hexadecyl	3-F-4-MeO-Bn	0.051 ± 0.018	0.240 ± 0.131	75.70	1484
	**12a**	tetradecyl	(*R*)-Bn	0.213 ± 0.097	0.575 ± 0.251	>100	>469

B.1.1.7 (Alpha)	V2043	octadecyl	(*R*)-Bn	0.146 ± 0.095	0.517 ± 0.169	58.27	399
B.1.351 (Beta)				0.146 ± 0.104	0.645 ± 0.459		399
P.1 (Gamma)				0.167 ± 0.084	0.362 ± 0.042		349
B.1.617.2 (Delta)				0.085 ± 0.024	0.484 ± 0.457		686
BA.1 (Omicron)				0.044 ± 0.012	0.359 ± 0.447		1324

a EC_50_, 50% effective inhibition concentration; EC_90_, 90% effective inhibition concentration; CC_50_, 50% cytotoxic concentration; SI, selectivity index = CC_50_/EC_50_. Assay type, immunofluorescence (IF). Mean values ± standard deviation values were derived from a minimum of at least 3 independent experiments performed in biological duplicate. CC_50_ values were determined by CellTiter-Glo (maximum concentration 100 mM). Assays were conducted at 44 hours post-infection (hpi). EC_50_, EC_90_, and CC_50_ values are in micromolar units and were calculated using Graphpad Prism 9 software.

**Table 2:** Effect of Compounds on SARS-CoV-2 (WA-1)-Infected Huh7.5 Cells^a^

Compound	EC_50_, μM	EC_90_, μM	CC_50_, μM	SI
V2043	0.140 ± 0.063	0.424 ± 0.267	55.8	399
**9c**	0.175 ± 0.120	0.519 ± 0.256	64.6	369
**9d**	0.191 ± 0.077	0.488 ± 0.199	75.0	392
**9g**	0.060 ± 0.037	0.212 ± 0.086	70.1	1168
**9h**	0.564 ± 0.252	3.364 ± 2.858	>100	>177
**9i**	0.092 ± 0.026	1.31 ± 0.31	69.8	758
**9j**	0.024 ± 0.002	0.070 ± 0.010	47.2	1966
**9k**	0.024 ± 0.006	0.077 ± 0.017	49.6	2066
**9l**	0.102 ± 0.017	0.281 ± 0.030	>100	>980
**10a**	0.109 ± 0.055	0.224 ± 0.100	>100	>917
**10g**	0.054 ± 0.016	0.095 ± 0.027	>100	>1851
**11a**	0.131 ± 0.066	0.268 ± 0.221	>100	>763
**11g**	0.050 ± 0.022	0.103 ± 0.047	59.0	1182
**12a**	0.157 ± 0.40	0.479 ± 0.019	>100	>636

a EC_50_, 50% effective inhibition concentration; EC_90_, 90% effective inhibition concentration; CC_50_, 50% cytotoxic concentration; SI, selectivity index = CC_50_/EC_50_. Assay type, immunofluorescence (IF). Mean values ± standard deviation values were derived from a minimum of at least 3 independent experiments performed in biological duplicate. CC_50_ values were determined by CellTiter-Glo (maximum concentration 100 mM). Assays were conducted at 48 hpi. EC_50_, EC_90_, and CC_50_ values are in micromolar units and were calculated using GraphPad Prism 9 software.

**Table 3:** Anti-SARS-CoV-2 (WA-1) Activity and Cytotoxicity in Vero-TMPRSS2 Cells^a^

Compound	EC_50_, μM	EC_90_, μM	CC_50_, μM	SI
V2043	0.216 ± 0.038	0.403 ± 0.128	97.9	453
**9c**	0.238 ± 0.074	0.613 ± 0.084	>100	>420
**9d**	0.271 ± 0.022	0.629 ± 0.061	>100	>369
**9g**	0.178 ± 0.057	0.294 ± 0.062	>100	>561
**9h**	0.775 ± 0.245	1.79 ± 0.14	>100	>129
**9i**	0.296 ± 0.010	0.582 ± 0.014	>100	>337
**10a**	0.293 ± 0.020	0.570 ± 0.054	>100	>341
**10g**	0.300 ± 0.038	0.554 ± 0.054	>100	>333
**11a**	0.402 ± 0.084	0.798 ± 0.154	>100	>248
**11g**	0.406 ± 0.087	0.858 ± 0.249	>100	>246
**12a**	0.909 ± 0.027	1.63 ± 0.12	>100	>110

a EC_50_, 50% effective inhibition concentration; EC_90_, 90% effective inhibition concentration; CC_50_, 50% cytotoxic concentration; SI, selectivity index = CC_50_/EC_50_. Assay type, IF, immunofluorescence (IF). Mean values ± standard deviation values were derived from a minimum of at least 3 independent experiments performed in biological duplicate. CC_50_ values were determined by CellTiter-Glo (maximum concentration 100 mM). Assays were conducted at 38 hpi for Vero-TMPRSS2 cells. EC_50_, EC_90_, and CC_50_ values are in micromolar units and were calculated using GraphPad Prism 9 software.

**Table 4:** Anti-SARS-CoV-2 (WA-1) Activity and Cytotoxicity in Human Pluripotent Stem Cell-Derived Lung Cells^a^

Compound	EC_50_, μM	EC_90_, μM	CC_50_, μM	SI
V2043	0.382 ± 0.205	0.736 ± 0.267	>20^IF^	>52
**9g**	0.167 ± 0.055	0.348 ± 0.121	>20^IF^	>119
**10a**	0.267 ± 0.118	0.555 ± 0.250	>20^IF^	>74
**10g**	0.082 ± 0.013	0.175 ± 0.016	>20^IF^	>243
**11g**	0.130 ± 0.056	0.267 ± 0.019	>20^IF^	>153

a EC_50_, 50% effective inhibition concentration; EC_90_, 90% effective inhibition concentration; CC_50_, 50% cytotoxic concentration; SI, selectivity index = CC_50_/EC_50_. Assay type = immunofluorescence (IF). Mean values ± standard deviation values were derived from independent experiments performed in biologic duplicate in lung cells derived from ALDA 31616 and H9 PSCs. CC_50_ values were determined by comparing relative number of nuclei present in compound vs vehicle-treated wells (maximum concentration 20 mM). Assays were conducted at 24 hpi. EC_50_, EC_90_, and CC_50_ values are in micromolar units and were calculated using GraphPad Prism 9 software.

**Table 5:** Antiviral Activity of RDV and RVn versus V2043 and Three Lead Compounds in Calu-3 Cells^a^

Compound	EC_50_, μM	EC_90_, μM	CC_50_, μM	SI
RDV^b^	0.23	0.31	>100	>434
RVn^b^	0.15	0.18	>100	>666
V2043	0.136 ± 0.056	0.323 ± 0.157	58.30	428
**9j**	0.043 ± 0.013	0.082 ± 0.022	60.20	1400
**9k**	0.034 ± 0.007	0.065 ± 0.036	61.55	1810
**10g**	0.070 ± 0.003	0.149 ± 0.117	58.70	839

a EC_50_, 50% effective inhibition concentration; EC_90_, 90% effective inhibition concentration; CC_50_, 50% cytotoxic concentration; SI, selectivity index = CC_50_/EC_50._ The CC_50_ curves have been placed in the Supporting Information (Figure S1).

b Data from Schooley et al.^24^ compared with data from Table 1.

The antiviral activity of V2043 is similar in Calu-3 and Huh7.5 cells (Tables 1–4). The rank order of activity of V2043 and the new analogs in the various cell lines is also similar except that compound **10g** appears less active in Vero-TMPRSS2 cells. In Calu-3 and Huh7.5 cells, CC_50_ values ranged from 58 to >100 and from 47 to >100. Values >100 and >20 were noted in Vero-TMPRSS2 and human pluripotent stem cell-derived lung cells. In general, selectivity indexes of 369 to 2066 were noted in Calu-3 and Huh7.5 cells. The absence of actual CC_50_ endpoints in Vero-TMPRSS2 and human pluripotent stem cells precluded calculation of meaningful selectivity indexes.

The antiviral activity values of V2043 and compounds **9j**, **9k**, and **10g** are greater than that of remdesivir based on EC_50_ as shown in Table 5. Compounds **9j**, **9k**, and **10g** are several-fold more active than RVn.

## Conclusions

We previously synthesized and evaluated 1-*O*-octadecyl-2-*O*-benzyl-*sn*-glyceryl-3-*phospho*-RVn (V2043) as a potential oral treatment for SARS-CoV-2. To further explore the structure/activity relationships in this class of molecules, we synthesized a series of compounds having substitutions at the *sn*-1 position of the glyceryl moiety. Long *sn*-1 alkyl ether groups from 14 to 18 carbons and an 18-carbon alkyl chain with a single unsaturation generally gave similar antiviral activity when the 2 position was occupied by an unsubstituted benzyl. To explore substituents at the *sn*-2 glyceryl hydroxyl position, we synthesized several prodrugs having short alkyl groups or substituted benzyls. Lower alkyl ether groups at the *sn*-2 position such as octyl or ethylcyclohexyl had little effect on antiviral activity compared to an unsubstituted benzyl. Substitutions at *sn*-2 of 3-F-benzyl, 3-CF_3_-benzyl, or 4-CF_3_-benzyl did not increase or reduce antiviral activity. However, marked increases in antiviral activity were noted with a 3-fluoro-4-methoxybenzyl substituent at *sn*-2 providing 2–3-fold better potency (**10g**). The largest increases in antiviral activity were noted with the 3- or 4-cyanobenzyl (**9j**, **9k**) compared to the unmodified benzyl of V2043. Compounds **9j**, **9k**, and **10g** are substantially more active against SARS-CoV-2 than RDV^24^ or VV116^15^*in vitro* and are expected to be orally active and stable in plasma.^24^

To preliminarily explore the potential *in vivo* effect of this class of compounds, we studied V2043 in a mouse model. After a 12h delay a dose of 60 mg/kg daily reduced lung viral load by 1.5 log_10_ units versus vehicle. Five days after infection both the 60 mg/kg daily and 30 mg/kg twice-daily regimens reduced lung viral load below the limit of detection, a reduction of >2.14 log_10_ units.

Further studies are underway to determine if the molecular modifications reported in this article provide improved efficacy in animal models of SARS-CoV-2 infection. Notably, this class of oral phospholipid-modified prodrugs of RVn also inhibits respiratory syncytial virus (RSV) and emerging RNA viruses that cause significant disease, including Ebola and Nipah viruses.^32^ Our findings warrant further development of these antivirals to combat SARS-CoV-2 and prepare for future outbreaks caused by other pandemic viruses.

## Experimental Section

### Chemistry General Procedures

**Figure 8:**
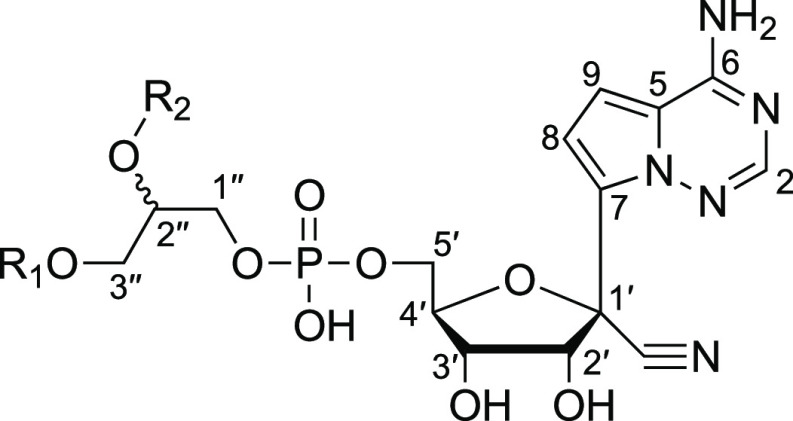
Numbering system used to assign NMR signals.

All reagents were of commercial quality and used without further purification unless indicated otherwise. Chromatographic purification was done using the flash method with silica gel 60 (EMD Chemicals, Inc., 230–400 mesh). ^1^H, ^13^C, and ^31^P nuclear magnetic resonance (NMR) spectra were recorded on a JEOL ECZ 400 MHz spectrometer and are reported in units of ppm relative to internal tetramethylsilane at 0.00 ppm. For the numbering system used to assign NMR spectra, see Figure 8. Mass spectrometry (MS) and high-performance liquid chromatography (HPLC) analyses were performed at the Molecular Mass Spectrometry Facility (MMSF) in the Department of Chemistry at the University of California, San Diego (UCSD). Electrospray ionization mass spectra (ESI-MS) were recorded on a Thermo LCQdeca mass spectrometer, and high-resolution mass spectra (HRMS) were obtained on an Agilent 6230 Accurate-Mass time-of-flight mass spectrometry system. Purity of UCSD-prepared target compounds was characterized by using an Agilent 1260 HPLC system. The analytical column was Phenomenex Synergi Polar-RP (4.6 × 150 mm, 4 μm). Mobile phase A was water with 0.1% formic acid, and mobile phase B was methanol/acetonitrile 1:1. At a flow rate of 1.0 mL/min, gradient elution was as follows: 5% B–95% B (0–10 min); 95% B (10–15 min); 95% B–5% B (15–16 min); 5% B (16–20 min). The post-run time was 1 min. Compounds were detected by ultraviolet light (UV) absorption wavelength at 274.0 nm. All compounds were determined to be >95% purity by HPLC analysis except for **9g**, which was 94.7% by HPLC. Homogeneity of compounds was also assessed by thin-layer chromatography (TLC) using Analtech silica gel-GF (250 μm) plates and the solvent system CHCl_3_/MeOH/conc. NH_4_OH/H_2_O (70:30:3:3 v/v). TLC results were visualized with UV light, iodine staining, phosphomolybdic acid spray, and charring at 400 °C.

### Compounds

Compounds **10a** and **10g** were synthesized at J-Star Research, Inc. (South Plainfield, NJ). Compounds **9d**, **9g**, **9h**, **9i**, **11a**, and **11g** were synthesized at Nanosyn, Inc. (Santa Clara, CA).

### General Method A – Tritylation

#### (*R*)-1-((4-Methoxyphenyl)diphenylmethoxy)-3-(octadecyloxy)propan-2-ol (**1-MMTr**)

Compound **1-MMTr** was prepared as described by Schott et al.^29^ except that the starting glycerol was 1-*O*-octadecyl-*sn*-glycerol. 4-Dimethylaminopyridine (DMAP, 116 mg, 0.952 mmol) was added to a solution of 1-*O*-octadecyl-*sn*-glycerol (**1**, 5.0 g, 14.5 mmol, Bachem Americas, Inc., Torrance, CA) and 4-monomethoxytrityl chloride (4.11 g, 14.76 mmol) in dry pyridine (60 mL). After the mixture was stirred at ambient temperature overnight, it was concentrated *in vacuo*, and the residue was dissolved in CH_2_Cl_2_ (50 mL) and extracted with H_2_O (2 × 25 mL). The organic layer was concentrated, and the residue was adsorbed onto silica gel and purified by flash column chromatography (gradient: 0 to 30% EtOAc in hexanes) to obtain **1-MMTr** (8.2 g, 92%) as a colorless oil. ^1^H NMR (400 MHz, CDCl_3_) δ = 6.70–7.48 (m, 14H, *aryl*), 3.91 (m, 1H, *H2″*), 3.75 (s, 3H, aryl-O-C*H*_3_), 3.49 (m, 2H, *H1″*), 3.44 (t, 2H, *J* = 6.8 Hz, O-C*H*_2_-(CH_2_)_16_-CH_3_), 3.17 (dd, 2H *J* = 5.5 Hz, 1.7 Hz, *H3″*), 1.26 (br s, 30H, -(C*H*_2_)_15_-), 0.87 (t, 3H, *J* = 6.6 Hz, -C*H*_3_).

### General Method B – Alkylation

#### (*R*)-((2-((3-Fluoro-4-methoxybenzyl)oxy)-3-(octadecyloxy)propoxy)(4-methoxyphenyl)methylene)dibenzene (**1g-MMTr**)

Sodium hydride (NaH, 97 mg, 4.0 mmol) was added to a cooled (0 °C) solution of **1-MMTr** (1 g, 1.62 mmol) and tetrabutylammonium iodide (180 mg, 0.49 mmol) in dry THF (25 mL), and the mixture was stirred vigorously for 20 min. 3-Fluoro-4-methoxybenzyl bromide (532 mg, 2.43 mmol) was then added, and the mixture was allowed to warm to ambient temperature and stirred overnight. The reaction was then quenched with water (0.5 mL), diluted with EtOAc (75 mL), washed with H_2_O (2 × 25 mL), and dried over anhydrous MgSO_4_. After evaporation, the residue was adsorbed onto silica gel and purified by flash column chromatography (gradient: 0 to 50% EtOAc- in hexanes) to yield **1g-MMTr** (880 mg, 72%) as a white solid. ^1^H NMR (400 MHz, CDCl_3_) δ 7.50–7.40 (m, 2H), 7.36–7.14 (m, 5H), 7.11 (dd, *J* = 12.0, 2.0 Hz, 2H), 6.95–6.76 (m, 5H), 4.57 (d, *J* = 2.6 Hz, 2H), 3.87 (s, 3H), 3.78 (s, 3H), 3.58–3.52 (m, 2H), 3.40 (t, *J* = 6.7 Hz, 1H), 3.20 (d, *J* = 5.1 Hz, 1H), 1.53 (h, *J* = 6.6 Hz, 2H), 1.25 (br s, 30H), 0.88 (m, 3H). ESI-MS *m*/*z* 777.65 [M+Na]^+^.

### General Method C – Detritylation

#### (*S*)-2-((3-Fluoro-4-methoxybenzyl)oxy)-3-(octadecyloxy)propan-1-ol (**1g**)

To a solution of **1g-MMTr** (880 mg, 1.16 mmol) in 1:1 CHCl_3_/MeOH (30 mL) was added *p*-toluenesulfonic acid (p-TsOH, 35 mg, 0.14 mmol), and the mixture was stirred at room temperature until deprotection was complete according to TLC analysis (approx. 3h). Saturated aq. NaHCO_3_ (200 mg) was added, and the solvent was evaporated under vacuum. The residue was adsorbed onto silica gel and purified by flash column chromatography (gradient: 0 to 10% EtOAc in hexanes) to afford compound **1g** as a clear oil (490 mg, 87%). ^1^H NMR (400 MHz, CDCl_3_) δ 7.10 (dd, *J* = 11.9, 2.1 Hz, 1H), 7.07–7.00 (m, 1H), 6.91 (t, *J* = 8.4 Hz, 1H), 4.62 (d, *J* = 11.7 Hz, 1H), 4.54 (d, *J* = 11.7 Hz, 1H), 3.87 (s, 3H), 3.78–3.69 (m, 1H), 3.69–3.59 (m, 2H), 3.59–3.48 (m, 2H), 3.43 (td, *J* = 6.7, 1.5 Hz, 2H), 1.61–1.49 (m, 2H), 1.24 (br s, 30H), 0.87 (m, 3H). ESI-MS *m*/*z* 481.4 [M+H]^+^.

### General Method D1 – Phosphorylation with Bis(2,2,2-trichloroethyl) phosphorochloridate (TCE-POCl)

#### (*R*)-2-((3-Fluoro-4-methoxybenzyl)oxy)-3-(octadecyloxy)propyl dihydrogen phosphate (**5g**)

Alcohol **1g** was phosphorylated with TCE-POCl and deprotected with zinc powder as described by Kates et al.^30^ To a solution of **1g** (483 mg, 1.0 mmol) and 1-methylimidazole (104 mg, 1.27 mmol) in dry pyridine (10 mL) was added a solution of bis(2,2,2-trichloroethyl) phosphorochloridate (482 mg, 1.27 mmol) in pyridine (3 mL) dropwise at ambient temperature. The reaction mixture was stirred overnight, then solvent was evaporated and co-evaporated with toluene (3 × 20 mL). The residue was dissolved in CH_2_Cl_2_, absorbed onto silica gel, and purified by flash column chromatography (gradient: 0 to 20% EtOAc in hexanes) to yield (*R*)-2-((3-fluoro-4-methoxybenzyl)oxy)-3-(octadecyloxy)propyl bis(2,2,2-trichloroethyl) phosphate (**5g-TCE**, 690 mg, 81%) as a colorless oil. ^1^H NMR (400 MHz, CDCl_3_) δ 7.12 (dd, *J* = 11.9, 2.1 Hz, 1H), 7.06 (ddd, *J* = 8.3, 2.1, 1.2 Hz, 1H), 6.92 (t, *J* = 8.4 Hz, 1H), 4.64–4.55 (m, 6H), 4.41 (ddd, *J* = 10.9, 7.3, 3.7 Hz, 1H), 4.26 (ddd, *J* = 10.9, 8.4, 5.5 Hz, 1H), 3.88 (s, 3H), 3.84–3.76 (m, 1H), 3.59–3.48 (m, 2H), 3.43 (t, *J* = 6.7 Hz, 2H), 1.55 (p, *J* = 6.1 Hz, 2H), 1.25 (s, 30H), 0.80 (t, 3H). ^13^C NMR (101 MHz, CDCl_3_) δ 152.42 (d, *J* = 246.2 Hz), 147.45 (d, *J* = 10.7 Hz), 130.99 (d, *J* = 5.9 Hz), 123.91 (d, *J* = 3.9 Hz), 115.96 (d, *J* = 18.4 Hz), 113.23, 94.75 (d, *J* = 11.2 Hz), 76.27 (d, *J* = 6.8 Hz), 72.04, 71.58, 69.40, 68.66 (d, *J* = 6.3 Hz), 56.40, 32.06, 30.29–28.90 (m), 26.23, 22.83, 14.27. ^31^P NMR δ −3.51. ESI-MS *m*/*z* 825.1 [M+H]^+^.

**5g-TCE** (690 mg, 0.84 mmol) was dissolved in a mixture of AcOH (glacial, 10 mL) and CHCl_3_ (8 mL) and cooled in an ice bath. Zinc powder (418 mg, 6.4 mmol) was added, and the mixture was stirred at 0 °C for 1h, then allowed to warm to ambient temperature while stirring was continued for 2h. The reaction mixture was filtered and the filtrate evaporated under vacuum. The residue was dissolved in a mixture of 20% MeOH/CH_2_Cl_2_ (30 mL) and extracted with 1 M aq. HCl (3 × 5 mL). The organic layer was concentrated and co-evaporated with EtOH (3 × 5 mL). The waxy residue was dissolved in 1,4-dioxane and lyophilized to provide phosphate **5g** (470 mg, 99%) as a white solid. ESI-MS *m*/*z* 563.5 [M+H]^+^.

### General Method D2 – Phosphorylation with POCl_3_

#### (*S*)-2-(Benzyloxy)-3-(octadecyloxy)propyl dihydrogen phosphate (**5b**)

Phosphates were also prepared as described by Ruiz et al.^33^ using POCl_3_. A solution of 3-*O*-octadecyl-2-*O*-benzyl-*sn*-glycerol (**1b**, 720 mg, 1.65 mmol) and triethylamine (0.60 mL, mmol) in CH_2_Cl_2_ (20 mL) was added to a cooled (0 °C) solution of POCl_3_ (0.3 mL 1.7 equiv) in CH_2_Cl_2_ (30 mL). After stirring 2–3h the solution was poured into ice/water and stirred vigorously for an additional 1h, then the organic layer was separated and concentrated to afford crude (*S*)-2-(benzyloxy)-3-(octadecyloxy)propyl dihydrogen phosphate (**5b**, 807 mg, 95%). ESI-MS *m*/*z* [M–H]^−^ 513.48. Phosphates were used without further purification in coupling steps.

### General Method E – Coupling

#### ((3a*R*,4*R*,6*R*,6a*R*)-6-(4-Aminopyrrolo[2,1-*f*][1,2,4]triazin-7-yl)-6-cyano-2,2-dimethyltetrahydrofuro[3,4-*d*][1,3]dioxol-4-yl)methyl ((*R*)-2-((3-fluoro-4-methoxybenzyl)oxy)-3-(octadecyloxy)propyl) hydrogen phosphate (**9g-acetonide**)

Diisopropylcarbodiimide (DIC, 204 mg, 1.62 mmol) was added to a solution of **5g** (303 mg, 0.54 mmol), GS-441524 acetonide (ACME Bioscience, 268 mg, 0.81 mmol), and 1-methylimidazole (NMI, 133 mg, 1.62 mmol) in dry pyridine (30 mL), and the reaction mixture was warmed to 35 °C and stirred overnight. Water (3 mL) was added, and the mixture was concentrated *in vacuo* and then co-evaporated with toluene (3 × 20 mL). The residue was taken up in CH_2_Cl_2_, adsorbed on silica gel, and purified by flash column chromatography (gradient: 0 to 10% MeOH in CH_2_Cl_2_) to provide **9g-acetonide** (137 mg, 29%) as an off-white solid. ESI-MS *m*/*z* 876.6 [M+H]^+^.

### General Method F – Deprotection

#### ((2*R*,3*S*,4*R*,5*R*)-5-(4-Aminopyrrolo[2,1-*f*][1,2,4]triazin-7-yl)-5-cyano-3,4-dihydroxytetrahydrofuran-2-yl)methyl ((*R*)-2-((3-fluoro-4-methoxybenzyl)oxy)-3-(octadecyloxy)propyl) hydrogen phosphate (**9g**)

Compound **9g-acetonide** (210 mg, 0.24 mmol) was added to formic acid (10 mL) at ambient temperature and stirred for 3h. The solution was concentrated *in vacuo* and co-evaporated with ethanol (2 × 50 mL), and the residue was dissolved in CH_2_Cl_2_, adsorbed onto silica gel, and purified by flash column chromatography (gradient: 0 to 20% MeOH in CH_2_Cl_2_) to produce **9g** (55 mg, 27%) as a white solid. ^1^H NMR (500 MHz, DMSO-*d*_6_) δ 8.43 (d, *J* = 6.7 Hz, 2H), 7.99–7.73 (m, 2H), 7.04 (d, *J* = 5.5 Hz, 1H), 6.87 (q, *J* = 3.7, 3.7, 3.2 Hz, 1H), 6.82 (dd, *J* = 7.3, 4.3 Hz, 1H), 6.17–5.74 (m, 1H), 4.59 (t, *J* = 4.9, 4.9 Hz, 1H), 4.53–4.41 (m, 1H), 4.11 (q, *J* = 4.9, 4.9, 4.9 Hz, 1H), 3.93 (q, *J* = 5.4, 5.4, 5.4 Hz, 1H), 3.84 (dq, *J* = 11.3, 6.5, 5.1, 5.1 Hz, 1H), 3.80–3.70 (m, 3H), 3.61 (ddd, *J* = 27.7, 10.9, 5.2 Hz, 3H), 3.30 (dd, *J* = 6.6, 3.0 Hz, 3H), 3.21 (dq, *J* = 9.8, 5.1, 5.1, 4.9 Hz, 1H), 1.43 (p, *J* = 6.6, 6.6, 6.5, 6.5 Hz, 2H), 1.27–1.16 (br s, 30H), 0.83 (t, *J* = 6.8 Hz, 3H). ESI-MS *m*/*z* [M+H]^+^ 836.5.

#### (*R*)-2-(Benzyloxy)-3-(octadecyloxy)propan-1-ol, **1b**

Synthesized by alkylation of (*S*)-(2,2-dimethyl-1,3-dioxolan-4-yl)methanol with octadecylmethanesulfonate according to the method of Kates et al.^30^ ESI-MS *m*/*z* [M+H]^+^ 435.53, [M+Na]^+^ 457.58.

#### (*S*)-2-(Benzyloxy)-3-(octadecyloxy)propyl dihydrogen phosphate, **5b**

Following General Method D2, a solution of alcohol **1b** (720 mg, 1.65 mmol) and triethylamine (416 mg, 4.1 mmol) was added to POCl_3_ (428 mg, 2.8 mmol) in CH_2_Cl_2_ (20 mL). Phosphate **5b** was isolated (800 mg, 94%) and used without further purification. ESI-MS *m*/*z* [M–H]^−^ 513.48.

#### ((3a*R*,4*R*,6*R*,6a*R*)-6-(4-Aminopyrrolo[2,1-*f*][1,2,4]triazin-7-yl)-6-cyano-2,2-dimethyltetrahydrofuro[3,4-*d*][1,3]dioxol-4-yl)methyl ((*S*)-2-(benzyloxy)-3-(octadecyloxy)propyl) hydrogen phosphate, **9b-acetonide**

Following General Method E, **5b** (800 mg, 1.55 mmol) was coupled to **RVn-acetonide** (1.55 mmol) using DIC (400 mg, 3.2 mmol) and NMI (390 mg, 4.75 mmol) in pyridine (20 mL). Compound **9b-acetonide** was isolated as an off-white solid (440 mg, 34%). ESI-MS *m*/*z* [M–H]^−^ 826.64.

#### ((2*R*,3*S*,4*R*,5*R*)-5-(4-Aminopyrrolo[2,1-*f*][1,2,4]triazin-7-yl)-5-cyano-3,4-dihydroxytetrahydrofuran-2-yl)methyl ((*S*)-2-(benzyloxy)-3-(octadecyloxy)propyl) hydrogen phosphate, **9b**

Following General Method F, **9b-acetonide** (440 mg, 0.53 mmol) was treated with formic acid (20 mL) to yield **9b** as an off-white solid (290 mg, 69%). ^1^H NMR (400 MHz, CD_3_OD + CDCl_3_) δ 7.85 (s, 1H, *H2*), 7.34–7.12 (m, 5H, -CH_2_-*aryl*), 6.95 (d, *J* = 4.6 Hz, 1H, *H9*), 6.87 (d, *J* = 4.6 Hz, 1H, *H8*), 4.80 (d, *J* = 5.4 Hz, 1H, *H2′*), 4.59 (d, *J* = 11.8 Hz, 1H, -C*H*_2_-aryl), 4.53 (d, *J* = 11.8 Hz, 1H, -C*H*_2_-aryl), 4.35 (m, 1H, *H4′*), 4.19 (t, *J* = 5.7 Hz, 1H, *H3′*), 4.12 (m, 1H, *H5′*), 4.05 (m, 1H, *H5′*), 3.87 (hept, *J* = 5.5 Hz, 2H, *H3″*), 3.70–3.63 (m, 1H, *H2″*), 3.49–3.37 (m, 2H, *H1″*), 3.37 (td, *J* = 6.6, 1.6 Hz, 2H, -OC*H*_2_CH_2_(CH_2_)_15_-), 1.49 (p, *J* = 6.6 Hz, 2H, -OCH_2_C*H*_2_(CH_2_)_15_-), 1.26 (br s, 30H, -OCH_2_CH_2_(C*H*_2_)_15_), 0.89 (t, 3H, -C*H*_3_). ^13^C NMR (101 MHz, CD_3_OD + CDCl_3_) δ 156.46 (*C6*), 147.50 (*C2*), 139.13 (-CH_2_-*aryl-C1*), 128.57 (-CH_2_-*aryl-C2 + C6*), 128.27 (-CH_2_-*aryl-C3 + C5*), 127.88 (-CH_2_-*aryl-C4*), 125.04 (*C7*), 117.15 (*C5*), 117.06 (-*C*N), 111.70 (*C9*), 102.02 (*C8*), 84.25 (d, *J* = 8.2 Hz, *C4′*), 80.23 (*C1′*), 77.96 (d, *J* = 8.4 Hz, *C3′*), 75.09 (*C2′*), 72.46 (*C2″*), 71.95 (-*C*H_2_-aryl), 71.03 (*C1″*), 70.93 (O-*C*H_2_CH_2_(CH_2_)_14_-), 65.53 (d, *J* = 5.3 Hz, *C3″*), 65.19 (*C5′*), 32.40 (-OCH_2_*C*H_2_(CH_2_)_14_-), 30.76–29.10 (m, -OCH_2_CH_2_(*C*H_2_)_14_-), 26.57 (-*C*H_2_CH_2_CH_3_), 23.07 (-CH_2_*C*H_2_CH_3_), 13.84 (-*C*H_3_). HRMS (ESI) *m*/*z* [M–H]^−^ calcd for C_40_H_61_N_5_O_9_P 786.4212, found 786.4204. HPLC purity 97.1%.

#### (*R,S*)-2-(Benzyloxy)-3-(octadecyloxy)propyl dihydrogen phosphate, **5c**

Following General Method D2, (*R,S*)-2-(benzyloxy)-3-(octadecyloxy)propan-1-ol (**1c**, purchased from Santa Cruz Biotechnology, Dallas, TX) was phosphorylated using POCl_3_. ESI-MS *m*/*z* [M–H]^−^ 513.45.

#### ((3a*R*,4*R*,6*R*,6a*R*)-6-(4-Aminopyrrolo[2,1-*f*][1,2,4]triazin-7-yl)-6-cyano-2,2-dimethyltetrahydrofuro[3,4-*d*][1,3]dioxol-4-yl)methyl ((*S*)-2-(benzyloxy)-3-(octadecyloxy)propyl) hydrogen phosphate, **9c-acetonide**

Following General Method E, **5c** (600 mg, 1.07 mmol) was coupled to GS-441524-acetonide (350 mg, 1.07 mmol) using DIC (270 mg, 2.14 mmol), NMI (260 mg, 3.21 mmol) in pyridine to yield **9c-acetonide** (370 mg, 42%). ESI-MS *m*/*z* [M+H]^+^ 828.41, [M+Na]^+^ 850.46.

#### ((2R,3S,4R,5R)-5-(4-aminopyrrolo[2,1-*f*][1,2,4]triazin-7-yl)-5-cyano-3,4-dihydroxytetrahydrofuran-2-yl)methyl ((*R,S*)-2-(benzyloxy)-3-(octadecyloxy)propyl) hydrogen phosphate, **9c**

Following General Method F, **9c-acetonide** (360 mg, 0.43 mmol) was added to formic acid (12 mL) and stirred 4h. Compound **9c** (310 mg, 91%) was isolated as a white solid. ^1^H NMR (400 MHz, CD_3_OD + CDCl_3_) δ 7.85 (s, 1H, *H2*), 7.36–7.15 (m, 5H, -CH_2_-*aryl*), 6.96 (dd, *J* = 4.6, 2.8 Hz, 1H, *H9*), 6.87 (dd, *J* = 4.7, 1.6 Hz, 1H, *H8*), 4.80 (dd, *J* = 8.8, 5.4 Hz, 1H, *H2′*), 4.63 (dd, *J* = 11.9, 2.5 Hz, 1H, -C*H*_2_-aryl), 4.57 (dd, *J* = 11.8, 7.4 Hz, 1H, C*H*_2_-aryl), 4.34 (q, *J* = 4.5 Hz, 1H, *H4′*), 4.22 (td, *J* = 5.5, 1.6 Hz, 1H, *H3′*), 4.14 (dtd, *J* = 11.1, 5.4, 3.5 Hz, 1H, *H5′*), 4.06 (dt, *J* = 11.5, 5.0 Hz, 1H, *H5′*), 3.89 (m, 2H, *H3″*), 3.70 (ddt, *J* = 6.2, 4.2, 2.5 Hz, 1H, *H2″*), 3.52–3.40 (m, 2H, *H1″*), 3.37 (m, 2H, -OC*H*_2_CH_2_(CH_2_)_15_-), 1.50 (p, *J* = 6.6 Hz, 2H, -OCH_2_C*H*_2_(CH_2_)_15_-), 1.27 (br s, 30H, -OCH_2_CH_2_(C*H*_2_)_15_-), 0.89 (t, 3H, -C*H*_3_). ^13^C NMR (101 MHz, CD_3_OD + CDCl_3_) δ 156.47 (*C6*), 147.49 (*C2*), 139.16 (-CH_2_-*aryl-C1*), 128.54 (-CH_2_-*aryl-C2 + C6*), 128.25 (-CH_2_-*aryl-C3 + C5*), 127.84 (-CH_2_-*aryl-C4*), 125.10 (*C7*), 117.12 (*C*5, *C*N), 111.67 (*C8*), 102.01 (*C9*), 84.42 (*C4′*), 80.10 (*C1′*), 79.99 (*C3′*), 77.95, 75.18 (*C2′*), 72.40 (*C2″*), 71.91 (*C*H_2_-aryl), 71.91 (*C1″*), 70.98 (O-*C*H_2_CH_2_(CH_2_)_14_-), 65.43 (*C3″*), 65.05 (*C5′*), 32.39 (-OCH_2_*C*H_2_(CH_2_)_14_-), 30.72–29.05 (m, -OCH_2_CH_2_(*C*H_2_)_14_-), 26.55 (-*C*H_2_CH_2_CH_3_), 23.06 (-CH_2_*C*H_2_CH_3_), 13.83 (-*C*H_3_). HRMS (ESI) *m*/*z* [M–H]^−^ calcd for C_40_H_61_N_5_O_9_P 786.4212, found 786.4206. HPLC purity 99.1%.

#### ((2*R*,3*S*,4*R*,5*R*)-5-(4-Aminopyrrolo[2,1-*f*][1,2,4]triazin-7-yl)-5-cyano-3,4-dihydroxytetrahydrofuran-2-yl)methyl ((*R*)-2-((3-fluorobenzyl)oxy)-3-(octadecyloxy)propyl) hydrogen phosphate, **9d**

Synthesized at Nanosyn (Santa Clara, CA). ^1^H NMR (400 MHz, CD_3_OD + CDCl_3_) δ 7.85 (s, 1H, H2), 7.31–7.21 (m, 1H, -CH_2_-*aryl-H5*), 7.14–7.04 (m, 1H, -CH_2_-*aryl-H4+H6*), 6.97 (d, *J* = 4.6 Hz, 1H, H9), 6.93 (tt, *J* = 7.8, 1.4 Hz, 1H, -CH_2_-*aryl-H2*), 6.87 (d, *J* = 4.6 Hz, 1H, H8), 4.81 (d, *J* = 5.3 Hz, 1H, H2′), 4.64 (d, *J* = 12.5 Hz, 1H, -C*H*_2_-aryl), 4.57 (d, *J* = 12.5 Hz, 1H, -C*H*_2_-aryl), 4.38–4.30 (m, 1H, H4′), 4.24 (t, *J* = 5.4 Hz, 1H, H3′), 4.13 (ddd, *J* = 11.5, 5.1, 3.5 Hz, 1H, H5′), 4.04 (dt, *J* = 11.5, 4.7 Hz, 1H, H5′), 3.86 (dq, *J* = 14.9, 5.4 Hz, 2H, H3″), 3.68 (qd, *J* = 5.4, 3.8 Hz, 1H, H2″), 3.46 (qd, *J* = 10.6, 5.0 Hz, 2H, H1″), 3.37 (tt, *J* = 5.4, 2.7 Hz, 2H, -OC*H*_2_CH_2_(CH_2_)_15_-), 1.51 (p, *J* = 6.4 Hz, 2H, -OCH_2_C*H*_2_(CH_2_)_15_-), 1.27 (br s, 30H, -OCH_2_CH_2_(C*H*_2_)_15_-), 0.89 (t, 3H, -CH_3_). ^13^C NMR (101 MHz, CD_3_OD + CDCl_3_) δ 164.29 (d, *J* = 244.1 Hz, -CH_2_-aryl-*C3*), 157.23 (*C6*), 148.23 (*C2*), 143.23 (-CH_2_-aryl-*C1*), 130.91 (d, *J* = 8.1 Hz, -CH_2_-aryl-*C3*), 125.97 (-CH_2_-aryl-*C6*), 124.19 (-CH_2_-aryl-*C4*), 117.85 (d, *J* = 10.6 Hz, -CH_2_-aryl-*C2*), 115.29 (*C7*), 115.08 (-*C*N), 114.87 (*C*5), 112.42 (*C*9), 102.71 (*C*8), 85.32 (d, *J* = 8.8 Hz, *C4′*), 80.68 (*C1′*), 79.05 (*C3′*), 76.02 (*C2′*), 72.61 (-*C*H_2_-aryl), 72.17 (-O*C*H_2_CH_2_(CH_2_)_14_-), 71.86 (d, *J* = 9.6 Hz, *C1″*), 66.07 (*C3″*), 65.64 (*C5′*), 33.13 (-OCH_2_*C*H_2_(CH_2_)_13_-), 31.69–29.82 (m, -OCH_2_CH_2_(*C*H_2_)_13_-), 27.30 (-*C*H_2_CH_2_CH_3_), 23.79 (-CH_2_*C*H_2_CH_3_), 14.50 (-*C*H_3_). HRMS (ESI) *m*/*z* [M–H]^−^ calcd for C_40_H_60_FN_5_O_9_P 804.4118, found 804.4126. HPLC 98.7%.

#### (*S*)-3-(Octadecyloxy)-2-((3-(trifluoromethyl)benzyl)oxy)propan-1-ol, **1e**

Following Method C, **1e-MMTr** (1.58 g, 2.07 mmol) was deprotected with p-TsOH in MeOH/CHCl_3_ to yield **1e** (540 mg, 52%). ESI-MS *m*/*z* [M+Na]^+^ 525.47.

#### (*R*)-3-(Octadecyloxy)-2-((3-(trifluoromethyl)benzyl)oxy)propyl dihydrogen phosphate, **5e**

Following General Method D2, a solution of alcohol **1e** (540 mg, 1.07 mmol) and triethylamine (270 mg, 2.67 mmol) was added to POCl_3_ (280 mg, 1.83 mmol) in CH_2_Cl_2_ (20 mL). Phosphate **5e** was isolated (670 mg, 95%) and used without further purification. ESI-MS *m*/*z* [M–H]^−^ 581.49.

#### ((3a*R*,4*R*,6*R*,6a*R*)-6-(4-Aminopyrrolo[2,1-*f*][1,2,4]triazin-7-yl)-6-cyano-2,2-dimethyltetrahydrofuro[3,4-*d*][1,3]dioxol-4-yl)methyl ((*R*)-3-(octadecyloxy)-2-((3-(trifluoromethyl)benzyl)oxy)propyl) hydrogen phosphate, **9e-acetonide**

Following General Method E, phosphate **5e** (670 mg, 1.15 mmol) was coupled to GS-441524-acetonide (380 mg, 1.15 mmol) using DIC (300 mg, 2.3 mmol) and NMI (200 mg, 2.3 mmol) in pyridine to yield **9e-acetonide** (540 mg, 52%). ESI-MS *m*/*z* [M–H]^−^ 894.66.

#### ((2*R*,3*S*,4*R*,5*R*)-5-(4-Aminopyrrolo[2,1-*f*][1,2,4]triazin-7-yl)-5-cyano-3,4-dihydroxytetrahydrofuran-2-yl)methyl ((*R*)-3-(octadecyloxy)-2-((3-(trifluoromethyl)benzyl)oxy)propyl) hydrogen phosphate, **9e**

Following General Method F, **9e-acetonide** (540 mg, 0.60 mmol) was added to formic acid (12 mL) and stirred 4h. Compound **9e** (250 mg, 49%) was isolated as an off-white solid. ^1^H NMR (400 MHz, CD_3_OD + CDCl_3_) δ 7.88 (s, 1H, *H2*), 7.62 (s, 1H, -CH_2_-aryl-*H2*), 7.57 (d, *J* = 7.5 Hz, 1H, -CH_2_-aryl-*H4*), 7.51 (d, *J* = 7.7 Hz, 1H, -CH_2_-aryl-*H5*), 7.46 (t, *J* = 7.6 Hz, 1H, -CH_2_-aryl-*H6*), 6.97 (d, *J* = 4.5 Hz, 1H, *H9*), 6.87 (d, *J* = 4.5 Hz, 1H, *H8*), 4.81 (d, *J* = 5.3 Hz, 1H, *H2′*), 4.72 (d, *J* = 12.4 Hz, 1H, -C*H*_2_-aryl), 4.65 (d, *J* = 12.4 Hz, 1H, -C*H*_2_-aryl), 4.35 (q, *J* = 4.4 Hz, 1H, *H4′*), 4.23 (t, *J* = 5.5 Hz, 1H, *H3′*), 4.15 (d, *J* = 11.5 Hz, 1H, H5′), 4.07 (d, *J* = 11.2 Hz, 1H, H5′), 3.90 (m, 2H, *H3″*), 3.71 (q, *J* = 5.0 Hz, 1H, *H2″*), 3.48–3.40 (m, 2H, *H1″*), 3.38 (tt, *J* = 5.5, 2.7 Hz, 2H, -OC*H*_2_CH_2_(CH_2_)_15_-), 1.50 (p, *J* = 6.6 Hz, 2H, -OCH_2_C*H*_2_(CH_2_)_15_-), 1.26 (br s, 30H, -OCH_2_CH_2_(C*H*_2_)_15_), 0.89 (t, 3H, -CH_3_). ^13^C NMR (101 MHz, CD_3_OD + CDCl_3_) δ 156.55 (*C6*), 147.43 (*C2*), 140.88 (-CH_2_-aryl-*C1*), 131.50 (-CH_2_-aryl-*C5*), 130.80 (q, *J* = 32.1 Hz, (-CH_2_-aryl-*C3*), 129.29 (-CH_2_-aryl-*C6*), 126.37 (-CH_2_-aryl-*C2*), 125.09 (-CH_2_-aryl-*C4*), 124.72 (q, *J* = 129 Hz, -*C*F_3_), 124.38 (*C7*), 123.67 (*C5*), 117.12 (-*C*N), 111.74 (*C8*), 102.09 (*C9*), 84.40 (*C4′*), 80.16 (*C1′*), 78.50 (*C2′*), 75.15 (*C2″*), 71.97 (-*C*H_2_-aryl), 71.49 (-O*C*H_2_CH_2_(CH_2_)_14_-), 71.12 (*C1″*), 71.04 (*C3′*), 65.50 (*C3″*), 65.14 (*C5′*), 32.40 (-OCH_2_*C*H_2_(CH_2_)_14_-), 30.70–29.12 (m), -OCH_2_CH_2_(*C*H_2_)_14_-), 26.55, (-*C*H_2_CH_2_CH_3_), 23.07, (-CH_2_*C*H_2_CH_3_), 13.82 (-*C*H_3_). HRMS (ESI) *m*/*z* [M–H]^−^ calcd for C_41_H_60_F_3_N_5_O_9_P 854.4086, found 854.4092. HPLC purity 97.3%.

#### (*S*)-3-(Octadecyloxy)-2-((4-(trifluoromethyl)benzyl)oxy)propan-1-ol, **1f**

Following Method C, **1f-MMTr** (1.67 g, 2.19 mmol) was deprotected with p-TsOH in MeOH/CHCl_3_ (20 mL) to yield **1f** (1.0 g, 87%). ESI-MS *m*/*z* [M+H]^+^ 503.35, [M+Na]^+^ 525.42.

#### (*R*)-3-(Octadecyloxy)-2-((4-(trifluoromethyl)benzyl)oxy)propyl dihydrogen phosphate, **5f**

Following General Method D2, a solution of alcohol **1f** (1.0 g, 1.9 mmol) and triethylamine (480 mg, 4.75 mmol) was added to POCl_3_ (495 mg, 3.23 mmol) in CH_2_Cl_2_ (25 mL). Phosphate **5f** was isolated (1.11 g, 99%) and used without further purification. ESI-MS *m*/*z* [M–H]^−^ 581.47.

#### ((3a*R*,4*R*,6*R*,6a*R*)-6-(4-Aminopyrrolo[2,1-*f*][1,2,4]triazin-7-yl)-6-cyano-2,2-dimethyltetrahydrofuro[3,4-*d*][1,3]dioxol-4-yl)methyl ((*R*)-3-(octadecyloxy)-2-((4-(trifluoromethyl)benzyl)oxy)propyl) hydrogen phosphate, **9f-acetonide**

Following General Method E, phosphate **5f** (1.1 g, 1.88 mmol) was coupled to RVn-acetonide (630 mg, 1.88 mmol) using DIC (470 mg, 3.76 mmol) and NMI (310 mg, 3.76 mmol) in pyridine (50 mL) to yield **9f-acetonide** (470 mg, 28%). ESI-MS *m*/*z* [M–H]^−^ 894.53.

#### ((2*R*,3*S*,4*R*,5*R*)-5-(4-Aminopyrrolo[2,1-*f*][1,2,4]triazin-7-yl)-5-cyano-3,4-dihydroxytetrahydrofuran-2-yl)methyl ((*R*)-3-(octadecyloxy)-2-((4-(trifluoromethyl)benzyl)oxy)propyl) hydrogen phosphate, **9f**

Following General Method F, **9f-acetonide** (450 mg, 0.50 mmol) was added to formic acid (20 mL) and stirred 4h. Compound **9f** (290 mg, 68%) was isolated as an off-white solid. ^1^H NMR (400 MHz, CD_3_OD + CDCl_3_) δ 7.81 (s, 1H, *H2*), 7.55 (d, *J* = 8.2 Hz, 2H, -CH_2_-aryl-*H3+ H5*), 7.49 (d, *J* = 8.2 Hz, 2H, -CH_2_-aryl-*H2 + H6*), 6.96 (d, *J* = 4.6 Hz, 1H, *H9*), 6.86 (d, *J* = 4.6 Hz, 1H, *H8*), 4.80 (d, *J* = 5.4 Hz, 1H, *H2′*), 4.72 (d, *J* = 12.8 Hz, 1H, -C*H*_2_-aryl), 4.66 (d, *J* = 12.8 Hz, 1H, -C*H*_2_-aryl), 4.36 (q, *J* = 4.5 Hz, 1H, *H4′*), 4.23 (t, *J* = 5.5 Hz, 1H, *H3′*), 4.14 (ddd, *J* = 11.6, 5.3, 3.5 Hz, 1H, *H5′*), 4.06 (dt, *J* = 11.5, 4.9 Hz, 1H, *H5′*), 3.89 (dq, *J* = 15.8, 5.5 Hz, 2H, *H3″*), 3.75–3.66 (m, 1H, *H2″*), 3.48 (qd, *J* = 10.6, 5.0 Hz, 2H, *H1″*), 3.38 (td, *J* = 6.6, 2.4 Hz, 2H, -OC*H*_2_CH_2_(CH_2_)_15_-), 1.50 (p, *J* = 6.6 Hz, 2H, -OCH_2_C*H*_2_(CH_2_)_15_-), 1.35–1.18 (m, 30H, -OCH_2_CH_2_(C*H*_2_)_15_), 0.89 (t, 3H, -C*H*_3_). ^13^C NMR (101 MHz, CD_3_OD + CDCl_3_) δ 156.32 (*C6*), 147.35 (*C2*), 143.79 (-CH_2_-aryl-*C1*), 129.67 (d, (-CH_2_-aryl-*C4*), 127.99 (-CH_2_-aryl-*C3 + C5*), 125.23 (-CH_2_-aryl-*C2 + C6*), 125.09 (q, *J* = 143 Hz, -CF_3_), 125.25 (*C7*), 124.92 (*C5*), 117.00 (-*C*N), 111.57 (*C*8), 101.94 (*C*9), 84.28 (*C4′*), 79.90 (*C1′*), 78.52 (*C2′*), 75.08 (*C2″*), 71.86 (-*C*H_2_-aryl), 71.35 (-O*C*H_2_CH_2_(CH_2_)_14_-), 71.03 (*C1″*), 70.86 (*C3′*), 65.40 (*C3″*), 64.93 (*C5′*), 32.26 (-OCH_2_*C*H_2_(CH_2_)_14_-), 30.50–29.32 (m, -OCH_2_CH_2_(*C*H_2_)_14_-), 26.45 (-*C*H_2_CH_2_CH_3_), 22.95 (-CH_2_*C*H_2_CH_3_), 13.80 (-*C*H_3_). HRMS (ESI) *m*/*z* [M–H]^−^ calcd for C_41_H_60_F_3_N_5_O_9_P 854.44086, found 854.4091. HPLC purity 99.2%.

#### ((2*R*,3*S*,4*R*,5*R*)-5-(4-Aminopyrrolo[2,1-*f*][1,2,4]triazin-7-yl)-5-cyano-3,4-dihydroxytetrahydrofuran-2-yl)methyl ((*R*)-3-(octadecyloxy)-2-(octyloxy)propyl) hydrogen phosphate, **9h**

Synthesized at Nanosyn, Santa Clara, CA. ^1^H NMR (400 MHz, CD_3_OD + CDCl_3_) δ 7.87 (s, 1H, *H2*), 6.98 (d, *J* = 4.6 Hz, 1H, *H9*), 6.89 (d, *J* = 4.6 Hz, 1H, *H8*), 4.82 (d, *J* = 5.3 Hz, 1H, *H2′*), 4.38–4.31 (m, 1H, *H4′*), 4.24 (t, *J* = 5.4 Hz, 1H, *H3′*), 4.14 (ddd, *J* = 11.5, 5.2, 3.7 Hz, 1H, *H5′*), 4.05 (dt, *J* = 11.5, 4.7 Hz, 1H, H5′), 3.80 (t, *J* = 5.5 Hz, 2H, *H3″*), 3.59–3.44 (m, 1H, *H2″*), 3.40 (qd, *J* = 6.5, 2.9 Hz, 4H, -OCH_2_C*H*_2_(CH_2_)_5_- + -OCH_2_C*H*_2_(CH_2_)_15_-), 1.57–1.42 (m, 4H, -OCH_2_C*H*_2_(CH_2_)_5_- + -OCH_2_C*H*_2_(CH_2_)_15_-), 1.37–1.17 (m, 40H, -OCH_2_CH_2_(C*H*_2_)_15_ + -OCH_2_CH_2_(C*H*_2_)_5_), 0.90 (t, 3H, -C*H*_3_), 0.88 (t, 3H, -C*H*_3_). ^13^C NMR (101 MHz, CD_3_OD + CDCl_3_) δ 156.50 (*C6*), 147.48 (*C2*), 125.26 (*C7*), 117.14 (-*C*N), 117.06 (*C5*), 111.72, (*C8*), 102.02 (*C9*), 84.52 (*C4′*), 80.00 (*C1′*), 78.59 (*C2′*), 75.32 (*C2″*), 71.90 (-O*C*H_2_CH_2_(CH_2_)_5_CH_3_), 71.13 (-O*C*H_2_CH_2_(CH_2_)_15_CH_3_), 71.05 (*C1″*), 70.82 (*C3′*), 65.19 (*C3″*), 64.86 (*C5′*), 32.40 (-OCH_2_*C*H_2_(CH_2_)_5_CH_3_), 32.37 (-OCH_2_*C*H_2_(CH_2_)_15_CH_3_), 30.10–29.81 (m, -(*C*H_2_)_5_ + (*C*H_2_)_15_), 26.58 (-(CH_2_)_5_*C*H_2_CH_2_*C*H_3_), 26.55 (-(CH_2_)_15_*C*H_2_CH_2_*C*H_3_), 23.07 (-(CH_2_)_6_*C*H_2_*C*H_3_ + -(CH_2_)_16_*C*H_2_*C*H_3_), 13.85 (-(CH_2_)_7_*C*H_3_), 13.83 (-(CH_2_)_17_*C*H_3_). HRMS (ESI) *m*/*z* [M–H]^−^ calcd for C_41_H_71_N_5_O_9_P 808.4995, found 808.5004. HPLC purity 99.7%.

#### ((2*R*,3*S*,4*R*,5*R*)-5-(4-Aminopyrrolo[2,1-*f*][1,2,4]triazin-7-yl)-5-cyano-3,4-dihydroxytetrahydrofuran-2-yl)methyl ((*R*)-2-(2-cyclohexylethoxy)-3-(octadecyloxy)propyl) hydrogen phosphate, **9i**

Synthesized at Nanosyn, Santa Clara, CA. ^1^H NMR (400 MHz, CD_3_OD + CDCl_3_) δ 7.86 (s, 1H, *H2*), 6.96 (d, *J* = 4.6 Hz, 1H, *H9*), 6.87 (d, *J* = 4.6 Hz, 1H, *H8*), 4.79 (d, *J* = 5.3 Hz, 1H, H2′), 4.36 (q, *J* = 4.4 Hz, 1H, *H4′*), 4.25 (t, *J* = 5.4 Hz, 1H, *H3′*), 4.12 (ddd, *J* = 11.5, 5.4, 3.8 Hz, 1H, *H5′*), 4.05 (ddd, *J* = 11.5, 5.6, 4.3 Hz, 1H, *H5′*), 3.81 (t, *J* = 5.4 Hz, 2H, *H3″*), 3.59–3.46 (m, 3H, *H2″* + *H1″*), 3.40 (ddt, *J* = 9.2, 6.2, 2.8 Hz, 4H, -OC*H*_2_CH_2_-), 1.70–1.56 (m, 4H, -OCH_2_*CH*_2_-), 1.52 (t, *J* = 7.0 Hz, 2H, -OCH_2_*CH*_2_-), 1.38 (qd, *J* = 6.5, 3.3 Hz, 2H, *alkyls*), 1.34–1.16 (m, 37H, *alkyls*), 0.84 (t, 3H, -C*H*_3_). ^13^C NMR (101 MHz, CD_3_OD + CDCl_3_) δ 156.43 (*C6*), 147.45 (C2), 125.09 (*C7*), 117.11 (-*C*N), 117.07 (*C5*), 111.69 (*C8*), 102.12 (*C9*), 84.47 (*C4′*), 79.94 (*C1′*), 78.55 (*C2′*), 75.31 (*C2″*), 72.03 (-O*C*H_2_CH_2_(CH_2_)_13_-), 71.10 (*C1″*), 70.97 (*C3′*), 68.77 (*C3″*), 65.20 (*C5′*), 64.86 (-O*C*H_2_-C_6_H_10_), 37.89 (-OCH_2_-C_6_H_10_-*C1*), 34.95, 33.87, 33.72, 32.39, 30.10–29.80 (m, alkyls), 27.00 + 26.73 + 26.58 (cycloalkyls), 23.08 (-CH_2_*C*H_2_CH_3_), 14.00 (-*C*H_3_). HRMS (ESI) *m*/*z* [M–H]^−^ calcd for C_41_H_69_N_5_O_9_P 806.4838, found 806.4847.

#### (*R*)-4-(((1-((4-Methoxyphenyl)diphenylmethoxy)-3-(octadecyloxy)propan-2-yl)oxy)methyl)benzonitrile, **1j-MMTr**

Following General Method B, sodium hydride (440 mg, 11 mmol) was added to a cooled (0 °C) solution of **1-MMTr** (3.39 g, 5.5 mmol) and tetrabutylammonium iodide (600 mg, 1.65 mmol) in dry THF (30 mL). After stirring vigorously for 20 min, 4-(bromomethyl)benzonitrile (1.61 g, 8.2 mmol) was added, and the mixture was allowed to warm to ambient temperature and stirred overnight. The reaction was then quenched with ice (25 mL), diluted with ethyl ether (75 mL), washed with H_2_O (2 × 25 mL), and dried over anhydrous MgSO_4_. The residue was adsorbed onto silica gel and purified by flash column chromatography (gradient: 0 to 50% EtOAc in hexanes) to yield **1j-MMTr** (610 mg, 15%) as a white solid. ESI-MS *m*/*z* [M+Na]^+^ 754.54.

#### (*S*)-4-(((1-Hydroxy-3-(octadecyloxy)propan-2-yl)oxy)methyl)benzonitrile, **1j**

Following General Method C, to a solution of **1j-MMTr** (400 mg, 0.54 mmol) in 1:1 CH_2_Cl_2_/MeOH (30 mL) was added *p*-toluenesulfonate monohydrate (5 mg, 0.03 mmol), and the mixture was stirred at room temperature until deprotection was complete (approx. 3h) according to TLC analysis. Saturated aq. NaHCO_3_ (200 mg) was added, and the solvent was evaporated under vacuum. The residue was adsorbed onto silica gel and purified by flash column chromatography (gradient: 0 to 10% EtOAc in hexanes) to afford compound **1j** as a clear oil (80 mg, 32%). ESI-MS *m*/*z* [M+Na]^+^ 482.55.

#### (*R*)-2-((4-Cyanobenzyl)oxy)-3-(octadecyloxy)propyl dihydrogen phosphate, **5j**

Following General Method D2, a solution of alcohol **1j** (80 mg, 0.17 mmol) and triethylamine (0.08 mg, 0.8 mmol) was added to a cooled (0 °C) solution of POCl_3_ (30 mg, 0.20 mmol) in CH_2_Cl_2_ (3 mL) and stirred for 3h. The mixture was added to acetone/ice water, stirred 1h, then extracted with CH_2_Cl_2_. Phosphate **5j** was isolated (80 mg, 87%) and used without further purification. ESI-MS *m*/*z* [M–H]^−^ 538.47.

#### ((3a*R*,4*R*,6*R*,6a*R*)-6-(4-Aminopyrrolo[2,1-*f*][1,2,4]triazin-7-yl)-6-cyano-2,2-dimethyltetrahydrofuro[3,4-*d*][1,3]dioxol-4-yl)methyl ((*R*)-2-((4-cyanobenzyl)oxy)-3-(octadecyloxy)propyl) hydrogen phosphate, **9j-acetonide**

Following General Method E, phosphate **5j** (200 mg, 0.37 mmol) was coupled to RVn-acetonide (132 mg, 0.40 mmol) using DIC (100 mg, 0.80 mmol) and NMI (98 mg, 1.2 mmol) in pyridine (10 mL) to yield **9j-acetonide** (60 mg, 19%). ESI-MS *m*/*z* [M–H]^−^ 851.55.

#### ((2*R*,3*S*,4*R*,5*R*)-5-(4-Aminopyrrolo[2,1-*f*][1,2,4]triazin-7-yl)-5-cyano-3,4-dihydroxytetrahydrofuran-2-yl)methyl ((*R*)-2-((4-cyanobenzyl)oxy)-3-(octadecyloxy)propyl) hydrogen phosphate, **9j**

Following General Method F, **9j-acetonide** (60 mg, 0.07 mmol) was added to formic acid (1 mL) and stirred 6h. Compound **9j** (20 mg, 35%) was isolated as an off-white solid. ^1^H NMR (400 MHz, CD_3_OD + CDCl_3_) δ 7.76 (s, 1H, *H2*), 7.60 (d, *J* = 8.0 Hz, 2H, -CH_2_-aryl-*H3+ H5*), 7.49 (d, *J* = 7.9 Hz, 2H, -CH_2_-aryl-*H2+ H6*), 6.96 (d, *J* = 4.4 Hz, 1H, *H9*), 6.86 (d, *J* = 4.5 Hz, 1H, *H8*), 4.80 (d, *J* = 5.3 Hz, 1H, *H2′*), 4.77–4.63 (m, 2H, -C*H*_2_-aryl), 4.37 (d, *J* = 4.7 Hz, 1H, *H4′*), 4.25 (t, *J* = 5.4 Hz, 1H, *H3′*), 4.11 (d, *J* = 18.7 Hz, 2H, *H5′*), 3.90 (s, 2H, *H3″*), 3.80–3.68 (m, 1H, *H2″*), 3.49 (qd, *J* = 10.6, 4.9 Hz, 2H, *H1″*), 3.39 (tt, *J* = 6.0, 3.0 Hz, 2H, -OC*H*_2_CH_2_(CH_2_)_15_-), 1.52 (p, *J* = 6.9 Hz, 2H, -OCH_2_C*H*_2_(CH_2_)_15_-), 1.26 (br s, *J* = 5.7 Hz, 30H, -OCH_2_CH_2_(C*H*_2_)_15_-), 0.89 (t, *J* = 6.8 Hz, 3H). ^13^C NMR (101 MHz, CD_3_OD + CDCl_3_) δ 156.63 (*C6*), 145.57 (*C2*), 132.62, (-CH_2_-aryl-*C1*), 128.47 (-CH_2_-aryl-*C3 + C5*), 125.14 (-CH_2_-aryl-*C2 + C6*), 119.38 (-*C*N), 117.29 (*C7*), 111.95 (*C5*), 111.28 (-*C*N′), 84.60 (*C4′*), 80.18 (*C1′*), 75.38 (*C2′*), 72.24 (*C2″*), 71.57 (-*C*H_2_-aryl), 71.40 (-O*C*H_2_CH_2_(CH_2_)_14_-), 71.12 (*C1″*), 65.75 (*C3″*), 65.20 (*C5′*), 32.57 (-OCH_2_*C*H_2_(CH_2_)_14_-), 30.89–29.57 (m) (m, -OCH_2_CH_2_(*C*H_2_)_14_-), 26.75 (-*C*H_2_CH_2_CH_3_), 23.26 (-CH_2_*C*H_2_CH_3_), 14.21 (-*C*H_3_). HRMS (ESI) *m*/*z* [M–H]^−^ calcd for C_41_H_60_N_6_O_9_P 811.4165, found 811.4178. HPLC purity 98.6%.

#### (*R*)-3-(((1-(Octadecyloxy)-3-(trityloxy)propan-2-yl)oxy)methyl)benzonitrile, **1k-Tr**

Prepared following General Method B except that the starting material was (*R*)-1-(octadecyloxy)-3-(trityloxy)propan-2-ol (**1-Tr**).^28^ Sodium hydride (60 mg, 2.5 mmol) was added to a cooled (0 °C) solution of **1-Tr** (540 mg, 0.92 mmol) and tetrabutylammonium iodide (180 mg, 0.5 mmol) in dry THF (30 mL). The mixture was stirred vigorously for 20 min before 3-(bromomethyl)benzonitrile (480 mg, 2.6 mmol) was added, and then the mixture was allowed to warm to ambient temperature and stirred overnight. The reaction was then quenched with ice (25 mL), diluted with ethyl ether (75 mL), washed with H_2_O (2 × 25 mL), and dried over anhydrous MgSO_4_. The residue was adsorbed onto silica gel and purified by flash column chromatography (gradient: 0 to 50% EtOAc in hexanes) to yield **1k-Tr** (600 mg, 93%) as a white solid. ESI-MS *m*/*z* [M+Na]^+^ 724.57.

#### (*S*)-3-(((1-Hydroxy-3-(octadecyloxy)propan-2-yl)oxy)methyl)benzonitrile, **1k**

Following General Method C, to a solution of **1k-Tr** (600 mg, 0.85 mmol) in 1:1 CH_2_Cl_2_/MeOH (30 mL) was added *p*-toluenesulfonate monohydrate (8 mg, 0.04 mmol), and the mixture was stirred at room temperature until deprotection was complete according to TLC analysis (approx. 3h). Saturated aq. NaHCO_3_ (200 mg) was added, and the solvent was evaporated under vacuum. The residue was adsorbed onto silica gel and purified by flash column chromatography (gradient: 0 to 10% EtOAc in hexanes) to afford compound **1k** as a clear oil (380 mg, 97%). ESI-MS *m*/*z* [M+H]^+^ 460.57, [M+Na]^+^ 482.52.

#### (*R*)-2-((3-Cyanobenzyl)oxy)-3-(octadecyloxy)propyl dihydrogen phosphate, **5k**

Following General Method D2, a solution of alcohol **1k** (360 mg, 0.78 mmol) and triethylamine (340 mg, 3.0 mmol) was added to a cooled (0 °C) solution of POCl_3_ (140 mg, 2.0 mmol) in CH_2_Cl_2_ (5 mL) and stirred for 3h. The mixture was added to acetone/ice water, stirred 1h, then extracted with CH_2_Cl_2_. Evaporation of the organic phase gave phosphate **5k** (328 mg, 78%), which was used without further purification. ESI-MS *m*/*z* [M–H]^−^ 538.46.

#### ((3a*R*,4*R*,6*R*,6a*R*)-6-(4-Aminopyrrolo[2,1-*f*][1,2,4]triazin-7-yl)-6-cyano-2,2-dimethyltetrahydrofuro[3,4-*d*][1,3]dioxol-4-yl)methyl ((*R*)-2-((3-cyanobenzyl)oxy)-3-(octadecyloxy)propyl) hydrogen phosphate, **9k-acetonide**

Following General Method E, phosphate **5k** (850 mg, 1.57 mmol) was coupled to RVn-acetonide (600 mg, 1.8 mmol) using DIC (450 mg, 3.6 mmol) and NMI (440 mg, 5.4 mmol) in pyridine (30 mL). Reaction at 35 °C overnight followed by chromatographic purification yielded **9k-acetonide** (360 mg, 26%). ESI-MS *m*/*z* [M+Na]^+^ 875.49.

#### ((2*R*,3*S*,4*R*,5*R*)-5-(4-Aminopyrrolo[2,1-*f*][1,2,4]triazin-7-yl)-5-cyano-3,4-dihydroxytetrahydrofuran-2-yl)methyl ((*R*)-2-((3-cyanobenzyl)oxy)-3-(octadecyloxy)propyl) hydrogen phosphate, **9k**

Following General Method F, **9k-acetonide** (340 mg, 0.39 mmol) was added to formic acid (7 mL) and stirred 3h. Compound **9k** (290 mg, 90%) was isolated as an off-white solid. ^1^H NMR (400 MHz, CD_3_OD + CDCl_3_) δ 7.71 (s, 1H, *H2*), 7.70 (s, 1H, -CH_2_-aryl-*H2*), 7.62 (d, *J* = 7.8 Hz, 1H, -CH_2_-aryl-*H4*), 7.56 (d, *J* = 7.8 Hz, 1H, -CH_2_-aryl-*H5*), 7.44 (t, *J* = 7.6 Hz, 1H, -CH_2_-aryl-*H6*), 6.96 (d, *J* = 4.5 Hz, 1H, *H9*), 6.86 (d, *J* = 4.5 Hz, 1H, *H8*), 4.72 (d, *J* = 5.3 Hz, 1H, *H2′*), 4.72 (d, *J* = 12.4 Hz, 1H, -C*H*_2_-aryl), 4.65 (d, *J* = 12.4 Hz, 1H, -C*H*_2_-aryl), 4.39 (q, *J* = 4.4 Hz, 1H, *H4′*), 4.27 (t, *J* = 5.5 Hz, 1H, *H3′*), 4.10 (d, *J* = 11.5 Hz, 1H, H5′), 3.89 (d, *J* = 11.2 Hz, 1H, H5′), 3.78 (m, 2H, *H3″*), 3.71 (q, *J* = 5.0 Hz, 1H, *H2″*),), 3.51–3.40 (m, 2H, *H1″*), 3.41 (tt, *J* = 5.5, 2.7 Hz, 2H, -OC*H*_2_CH_2_(CH_2_)_15_-), 1.54 (p, *J* = 6.6 Hz, 2H, -OCH_2_C*H*_2_(CH_2_)_15_-), 1.26 (br s, 30H, -OCH_2_CH_2_(C*H*_2_)_15_), 0.89 (t, 3H, -C*H*_3_). ^13^C NMR (101 MHz, CD_3_OD + CDCl_3_) δ 156.64 (*C6*), 147.68 (*C2*), 141.50 (-CH_2_-aryl-*C1*), 132.77 (-CH_2_-aryl-*C2*), 131.73(-CH_2_-aryl-*C4*), 131.57 (-CH_2_-aryl-*C6*), 129.88 (-CH_2_-aryl-*C5*), 125.25 (C7), 119.50 (C5), 117.41 (-CN), 112.62 (-CN′), 111.98 (C8), 102.47 (C9), 84.91 (*C4′*), 80.07 (*C1′*), 75.57 (*C2″*), 72.40 (-*C*H_2_-aryl), 71.58 (-O*C*H_2_CH_2_(CH_2_)_14_-), 71.44 (C1″), 71.23 (C3′), 65.73 (C3″), 65.21 (*C5′*), 32.67 (-OCH_2_*C*H_2_(CH_2_)_14_-), 31.02–29.33 (m, -OCH_2_CH_2_(*C*H_2_)_14_-), 26.85 (-*C*H_2_CH_2_CH_3_), 23.37 (-CH_2_*C*H_2_CH_3_), 14.39 (-*C*H_3_). HRMS (ESI) *m*/*z* [M–H]^−^ calcd for C_41_H_60_N_6_O_9_P 811.4165, found 811.4178. HPLC purity 98.6%.

#### (*R*)-2-(((1-(Octadecyloxy)-3-(trityloxy)propan-2-yl)oxy)methyl)benzonitrile, **1l-Tr**

Prepared following General Method B except that the starting material was (*R*)-1-(octadecyloxy)-3-(trityloxy)propan-2-ol (**1-Tr**).^28^ Sodium hydride (60 mg, 2.5 mmol) was added to a cooled (0 °C) solution of **1-Tr** (587 mg, 1.0 mmol) and tetrabutylammonium iodide (180 mg, 0.5 mmol) in dry THF (30 mL). The mixture was stirred vigorously for 20 min before 2-(bromomethyl)benzonitrile (455 mg, 2.5 mmol) was added, and then the solution was allowed to warm to ambient temperature overnight. The reaction was then quenched with ice (25 mL), diluted with ethyl ether (75 mL), washed with H_2_O (2 × 25 mL), and dried over anhydrous MgSO_4_. The residue was adsorbed onto silica gel and purified by flash column chromatography (gradient: 0 to 50% EtOAc in hexanes) to yield **1l-Tr** (580 mg, 83%) as a white solid. ESI-MS *m*/*z* [M+Na]^+^ 724.62.

#### (*S*)-2-(((1-Hydroxy-3-(octadecyloxy)propan-2-yl)oxy)methyl)benzonitrile, **1l**

Following General Method C, to a solution of **1l-Tr** (580 mg, 0.83 mmol) in 1:1 CH_2_Cl_2_/MeOH (15 mL) was added *p*-toluenesulfonate monohydrate (8 mg, 0.04 mmol), and the mixture was stirred at room temperature until deprotection was complete (approx. 3h) according to TLC analysis. Saturated aq. NaHCO_3_ (200 mg) was added, and the solvent was evaporated under vacuum. The residue was adsorbed onto silica gel and purified by flash column chromatography (gradient: 0 to 10% EtOAc in hexanes) to afford compound **1l** as a clear oil (380 mg, 97%). ESI-MS *m*/*z* [M+H]^+^ 460.39, [M+Na]^+^ 482.56.

#### (*R*)-2-((2-Cyanobenzyl)oxy)-3-(octadecyloxy)propyl dihydrogen phosphate, **5l**

Following General Method D2, a solution of alcohol **1l** (360 mg, 0.78 mmol) and triethylamine (340 mg, 3.0 mmol) was added to a cooled (0 °C) solution of POCl_3_ (140 mg, 2.0 mmol) in CH_2_Cl_2_ (5 mL) and stirred for 3h. The mixture was added to acetone/ice water, stirred 1h, then extracted with CH_2_Cl_2_. Evaporation of the organic phase gave phosphate **5l** (374 mg, 89%), which was used without further purification. ESI-MS *m*/*z* [M–H]^−^ 538.46.

#### ((3a*R*,4*R*,6*R*,6a*R*)-6-(4-Aminopyrrolo[2,1-*f*][1,2,4]triazin-7-yl)-6-cyano-2,2-dimethyltetrahydrofuro[3,4-*d*][1,3]dioxol-4-yl)methyl ((*R*)-2-((2-cyanobenzyl)oxy)-3-(octadecyloxy)propyl) hydrogen phosphate, **9l-acetonide**

Following General Method E, phosphate **5l** (460 mg, 0.85 mmol) was coupled to RVn-acetonide (280 mg, 0.85 mmol) using DIC (210 mg, 1.7 mmol) and NMI (140 mg, 1.7 mmol) in dry pyridine (15 mL). Reaction at 35 °C for 23h followed by chromatographic purification yielded **9l-acetonide** (190 mg, 22%). ESI-MS *m*/*z* [M+H]^+^ 851.47.

#### ((2*R*,3*S*,4*R*,5*R*)-5-(4-Aminopyrrolo[2,1-*f*][1,2,4]triazin-7-yl)-5-cyano-3,4-dihydroxytetrahydrofuran-2-yl)methyl ((*R*)-2-((2-cyanobenzyl)oxy)-3-(octadecyloxy)propyl) hydrogen phosphate, **9l**

Following General Method F, **9l-acetonide** (190 mg, 0.22 mmol) was added to formic acid (5 mL) and stirred 3h. Formic acid was evaporated, and the residue was purified by flash column chromatography to give compound **9l** as an off-white solid (120 mg, 67%). ^1^H NMR (400 MHz, CD_3_OD + CDCl_3_) δ 7.73 (s, 1H, H2), 7.65 (d, *J* = 7.6, Hz, 2H, -CH_2_-aryl-*H3* + *H5*), 7.59 (td, *J* = 7.6, 1.3 Hz, 1H, *H4*), 7.39 (td, *J* = 7.6, 1.3 Hz, 1H, *H6*), 6.97 (d, *J* = 4.6 Hz, 1H, *H9*), 6.86 (d, *J* = 4.5 Hz, 1H, *H8*), 4.81 (d, *J* = 5.7 Hz, 1H, *H2′*), 4.38 (q, *J* = 4.2 Hz, 1H, *H4′*), 4.28 (t, *J* = 5.3 Hz, 1H, *H3′*), 4.19–3.84 (m, 2H, *H5′*), 3.93 (tp, *J* = 10.7, 4.7 Hz, 2H, *H3″*), 3.85–3.70 (m, 1H, *H2″*), 3.54 (qd, *J* = 10.6, 5.0 Hz, 2H, *H1″*), 3.41 (td, *J* = 6.6, 2.7 Hz, 2H, -OC*H*_2_CH_2_(CH_2_)_15_-), 1.52 (p, *J* = 6.6 Hz, 2H, -OCH_2_C*H*_2_(CH_2_)_15_-), 1.26 (m, *J* = 7.2 Hz, 30H, -OCH_2_CH_2_(C*H*_2_)_15_-), 0.89 (t, 3H, -C*H*_3_). ^13^C NMR (101 MHz, CD_3_OD + CDCl_3_) δ 156.53 (*C6*), 147.49 (*C2*), 142.96 (-CH_2_-aryl-*C1*), 133.62, (-CH_2_-aryl-*C3*), 133.23, (-CH_2_-aryl-*C6*), 129.66, (-CH_2_-aryl-*C5*), 128.68, (-CH_2_-aryl-*C4*), 125.15, (*C7*), 117.85, (*C5*), 117.26, (-*C*N), 111.84, (-*C*N′), 111.58, (*C8*), 102.35, (*C9*), 84.75, (*C4′*), 79.89 (*C1′*), 78.99 (*C2′*), 75.45 (*C2″*), 72.21 (-*C*H_2_-aryl), 71.30 (O*C*H_2_CH_2_(CH_2_)_14_-), 71.09 (*C1″*), 70.45 (*C3′*),65.73 (*C3″*), 65.03 (*C5′*), 32.51 (-OCH_2_*C*H_2_(CH_2_)_14_-), 31.09–29.39 (m, -OCH_2_CH_2_(*C*H_2_)_14_-), 26.67, (-*C*H_2_CH_2_CH_3_), 23.21 (-CH_2_*C*H_2_CH_3_), 14.19 (-*C*H_3_). HRMS (ESI) *m*/*z* [M+H]^+^ calcd for C_41_H_62_N_6_O_9_P 813.4310, found 813.4302. HPLC purity 99.4%.

#### ((2*R*,3*S*,4*R*,5*R*)-5-(4-Aminopyrrolo[2,1-*f*][1,2,4]triazin-7-yl)-5-cyano-3,4-dihydroxytetrahydrofuran-2-yl)methyl ((*R*)-2-(benzyloxy)-7-(tetradec-5-en-1-yloxy)heptyl) hydrogen phosphate, **10a**

Synthesized at J-Star Research, South Plainfield, NJ. ^1^H NMR (400 MHz, CD_3_OD + CDCl_3_) δ 7.91 (s, 1H, *H2*), 7.37–7.15 (m, 5H, -CH_2_-*aryl*), 7.06 (d, *J* = 4.7 Hz, 1H, *H9*), 7.04 (d, *J* = 4.7 Hz, 1H, *H8*), 5.33 (td, *J* = 5.0, 1.1 Hz, 2H, -C*H*=C*H*-), 4.77 (d, *J* = 5.2 Hz, 1H, *H2′*), 4.64 (d, *J* = 11.9 Hz, 1H, -C*H*_2_-aryl), 4.59 (d, *J* = 11.9 Hz, 1H, -C*H*_2_-aryl), 4.34 (dt, *J* = 6.2, 2.6 Hz, 1H, H4′), 4.24 (t, *J* = 5.4 Hz, 1H, H3′), 4.16 (ddd, *J* = 11.6, 5.3, 3.4 Hz, 1H, H5′), 4.06 (dt, *J* = 11.5, 4.7 Hz, 1H, H5′), 3.98–3.83 (m, 2H, *H3″*), 3.71 (dt, *J* = 9.4, 4.6 Hz, 1H, *H2″*), 3.56–3.44 (m, 2H, *H1″*), 3.39 (td, *J* = 6.6, 1.8 Hz, 2H, -OC*H*_2_CH_2_CH_2_−), 2.01 (tt, *J* = 7.9, 2.9 Hz, 4H, -C*H*_2_CH=CHC*H*_2_-), 1.51 (p, *J* = 6.6 Hz, 2H, -OCH_2_C*H*_2_CH_2_-), 1.29 (br s, 22H, -(C*H*_2_)_6_CH_2_CH=CHCH_2_(C*H*_2_)_5_CH_3_), 0.89 (t, 3H, -C*H*_3_). ^13^C NMR (101 MHz, CD_3_OD + CDCl_3_) δ 153.73 (*C6*), 142.98 (*C2*), 139.25 (-CH_2_-aryl-*C1*), 130.18 (-*C*H=*C*H-), 130.14 (-*C*H=*C*H-), 128.52 (-CH_2_-aryl-*C3 + C5*), 128.15 (-CH_2_-aryl-*C2+C6*), 127.79 (-CH_2_-aryl-*C4*), 127.54 (*C7*), 116.88 (-*C*N), 115.90 (*C5*), 112.80 (*C8*), 105.24 (*C9*), 84.60 (*C4′*), 79.83 (*C1′*), 77.97 (*C2′*), 72.39 (*C2″*), 71.91 (-*C*H_2_-aryl), 71.07 (-O*C*H_2_CH_2_(CH_2_)_6_CH_2_CH=CH-), 70.85 (*C3′*), 65.54 (*C3″*), 64.79 (*C5′*), 32.39 (-OCH_2_*C*H_2_(CH_2_)_6_CH_2_CH=CH-), 30.68–29.05 (m, -(*C*H_2_)_5_CH_2_CH=CHCH_2_(*C*H_2_)_5_CH_3_), 27.46 (-*C*H_2_CH=CH*C*H_2_-), 26.57 (-OCH_2_CH_2_*C*H_2_(CH_2_)_5_CH_2_CH=CH-), 23.07 (-CH_2_*C*H_2_CH_3_), 13.85(-*C*H_3_). HRMS (ESI) *m*/*z* [M–H]^−^ calcd for C_40_H_59_N_5_O_9_P 784.4056, found 784.4067. HPLC purity >99%.

#### ((2*R*,3*S*,4*R*,5*R*)-5-(4-Aminopyrrolo[2,1-*f*][1,2,4]triazin-7-yl)-5-cyano-3,4-dihydroxytetrahydrofuran-2-yl)methyl ((*R*)-2-((3-fluoro-4-methoxybenzyl)oxy)-7-(tetradec-5-en-1-yloxy)heptyl) hydrogen phosphate, **10g**

Synthesized at J-Star Research, South Plainfield, NJ. ^1^H NMR (400 MHz, CD_3_OD + CDCl_3_) δ 7.92 (s, 1H, *H2*), 7.13–7.00 (m, 3H, -CH_2_-*aryl*), 6.95 (d, *J* = 8.4 Hz, 1H, *H9 + H8*), 5.32 (td, *J* = 4.5, 2.2 Hz, 2H, -C*H*=C*H*-), 4.77 (d, *J* = 5.2 Hz, 1H, *H2′*), 4.57 (d, *J* = 11.8 Hz, 1H, -C*H*_2_-aryl), 4.50 (d, *J* = 11.8 Hz, 1H, -C*H*_2_-aryl), 4.34 (dd, *J* = 5.6, 3.1 Hz, 1H, *H4′*), 4.24 (t, *J* = 5.4 Hz, 1H, *H3′*), 4.16 (ddd, *J* = 11.6, 5.2, 3.4 Hz, 1H, *H5′*), 4.06 (dt, *J* = 11.5, 4.7 Hz, 1H, *H5′*), 3.89 (dq, *J* = 14.2, 5.3 Hz, 2H, *H3″*), 3.82 (s, 3H, -OC*H*_3_), 3.74–3.66 (m, 1H, *H2″*), 3.56–3.42 (m, 2H, H1, *H1″*), 3.39 (td, *J* = 6.6, 1.3 Hz, 2H, OC*H*_2_CH_2_CH_2_-), 2.13–1.90 (m, 4H, -C*H*_2_CH=CHC*H*_2_-), 1.51 (p, *J* = 6.8 Hz, 2H, -OCH_2_C*H*_2_CH_2_-), 1.42–1.20 (m, 22H, -(C*H*_2_)_6_CH_2_CH=CHCH_2_(C*H*_2_)_5_CH_3_),), 0.89 (t, 3H, -C*H*_3_). ^13^C NMR (101 MHz, CD_3_OD + CDCl_3_) δ 154.67 (*C6*), 153.58 (d, *J* = 244.9 Hz, -CH_2_-aryl-*C3*), 148.55 (*C2*), 148.45 (-CH_2_-aryl-*C4*), 143.95 (CH_2_-aryl-*C1*), 133.22 (-*C*H=*C*H-), 131.01 (-*C*H=*C*H-), 128.36 (-CH_2_-aryl-*C2*), 125.00 (*C7*), 117.73 (-*C*N), 116.76 (*C5*), 116.71 (-CH_2_-aryl-*C6*), 116.52 (-CH_2_-aryl-*C5*), 113.65 (*C8*), 105.99 (*C9*), 85.47 (*C4′*), 85.39 (*C1′*), 80.69 (*C2′*), 78.76 (*C3′*), 78.68 (*C2″*), 72.76 (-*C*H_2_-aryl), 72.28 (*C1″*), 71.99 (-O*C*H_2_CH_2_(CH_2_)_6_CH_2_CH=CH-), 71.68 (*C3″*), 66.37 (*C5′*), 33.23 (-OCH_2_*C*H_2_(CH_2_)_6_CH_2_CH=CH-), 31.29–30.13 (m, -(*C*H_2_)_5_CH_2_CH=CHCH_2_(*C*H_2_)_5_CH_3_), 28.29 (-*C*H_2_CH=CH*C*H_2_-), 27.43 (-OCH_2_CH_2_*C*H_2_(CH_2_)_5_CH_2_CH=CH-), 23.92 (-CH_2_*C*H_2_CH_3_), 14.69 (-*C*H_3_). HRMS (ESI) *m*/*z* [M–H]^−^ calcd for C_41_H_60_N_5_O_10_P 832.4067, found 832.4073. HPLC purity >99%.

#### ((2*R*,3*S*,4*R*,5*R*)-5-(4-Aminopyrrolo[2,1-*f*][1,2,4]triazin-7-yl)-5-cyano-3,4-dihydroxytetrahydrofuran-2-yl)methyl ((*R*)-2-(benzyloxy)-3-(hexadecyloxy)propyl) hydrogen phosphate, **11a**

Synthesized at Nanosyn, Santa Clara, CA. ^1^H NMR (400 MHz, CD_3_OD + CDCl_3_) δ 7.85 (s, 1H, H2), 7.37–7.16 (m, 5H, -CH_2_-aryl), 6.97 (d, *J* = 4.6 Hz, 1H, H9), 6.87 (d, *J* = 4.6 Hz, 1H, H8), 4.79 (d, *J* = 5.3 Hz, 1H, H2′), 4.63 (d, *J* = 11.8 Hz, 1H, -CH_2_-aryl), 4.57 (d, *J* = 11.9 Hz, 1H, -CH_2_-aryl), 4.37–4.31 (m, 1H, H4′), 4.24 (t, *J* = 5.3 Hz, 1H, H3′), 4.12 (ddd, *J* = 11.5, 5.1, 3.6 Hz, 1H, H5′), 4.04 (ddd, *J* = 11.5, 5.2, 4.2 Hz, 1H, H5′), 3.86 (dq, *J* = 12.6, 5.3 Hz, 2H, H3″), 3.69 (qd, *J* = 5.4, 3.8 Hz, 1H, H2″), 3.55–3.41 (m, 2H, H1″), 3.37 (td, *J* = 6.6, 1.9 Hz, 2H, -OCH_2_CH_2_(CH_2_)_13_-), 1.50 (p, *J* = 6.6 Hz, 2H, -OCH_2_CH_2_(CH_2_)_13_-), 1.27 (br s, 26H, -OCH_2_CH_2_(CH_2_)_13_-), 0.90 (t, 3H, -CH_3_). ^13^C NMR (101 MHz, CD_3_OD + CDCl_3_) δ 156.49 (C6), 147.47 (C2), 139.29 (CH_2_-aryl-C1), 128.51 (CH_2_-aryl-C2 + C6), 128.20 (CH_2_-aryl-C3 + C5), 127.77 (CH_2_-aryl-C4), 125.29 (C7), 117.19 (-CN), 117.04 (C5), 111.69 (C8), 102.03 (C9), 84.65 (C4′), 79.86 (C1′), 77.91 (C2′), 75.37 (C2″), 72.36 (-CH_2_-aryl), 71.88 (-OCH_2_CH_2_(CH_2_)_12_-), 71.17 (C1″), 71.06 (C3′), 65.29 (C3″), 64.85 (C5′), 32.40 (-OCH_2_CH_2_(CH_2_)_11_-), 30.81–29.18 (m, -OCH_2_CH_2_(CH_2_)_11_-), 26.56 (-CH_2_CH_2_CH_3_), 23.07 (-CH_2_CH_2_CH_3_), 13.82 (-CH_3_). HRMS (ESI) *m*/*z* [M–H]^−^ calcd for C_41_H_69_N_5_O_10_P 758.3899, found 758.3907. HPLC purity 99.8%.

#### ((2*R*,3*S*,4*R*,5*R*)-5-(4-Aminopyrrolo[2,1-*f*][1,2,4]triazin-7-yl)-5-cyano-3,4-dihydroxytetrahydrofuran-2-yl)methyl ((*R*)-2-((3-fluoro-4-methoxybenzyl)oxy)-3-(hexadecyloxy)propyl) hydrogen phosphate, **11g**

Synthesized at Nanosyn, Santa Clara, CA. ^1^H NMR (400 MHz, CD_3_OD + CDCl_3_) δ 7.85 (s, 1H, *H2*), 7.09–6.97 (m, 4H, -CH_2_-*aryl-H2,4,5,6*), 6.96 (d, *J* = 4.6 Hz, 1H, *H9*), 6.86 (d, *J* = 4.6 Hz, 1H, *H8*), 4.80 (d, *J* = 5.3 Hz, 1H, *H2′*), 4.55 (d, *J* = 11.8 Hz, 1H, -C*H*_2_-aryl), 4.48 (d, *J* = 11.8 Hz, 1H, -C*H*_2_-aryl), 4.38–4.31 (m, 1H, *H4′*), 4.23 (t, *J* = 5.5 Hz, 1H, *H3′*), 4.14 (ddd, *J* = 11.5, 5.2, 3.5 Hz, 1H, *H5′*), 4.05 (dt, *J* = 11.5, 4.9 Hz, 1H, *H5′*), 3.92–3.84 (m, 1H, *H3″*), 3.83 (s, 2H, *H3″*), 3.71–3.62 (m, 1H, *H2″*), 3.45 (qd, *J* = 10.6, 5.1 Hz, 2H, *H1″*), 3.37 (td, *J* = 6.6, 1.3 Hz, 2H, -OC*H*_2_CH_2_(CH_2_)_13_-), 1.50 (p, *J* = 6.7 Hz, 2H, -OCH_2_C*H*_2_(CH_2_)_13_-), 1.39–1.18 (m, 26H, -OCH_2_CH_2_(C*H*_2_)_13_-), 0.89 (m, 3H, -C*H*_3_). ^13^C NMR (101 MHz, CD_3_OD + CDCl_3_) δ 156.49 (*C6*), 152.78 (d, *J* = 244.9 Hz, -CH_2_-aryl-*C3*), 147.76 (*C2*), 147.65 (-CH_2_-aryl-*C4*), 147.51 (-CH_2_-aryl-*C1*), 132.41 (-CH_2_-aryl-*C6*), 125.19 (*C7*), 124.20 (d, *J* = 3.6 Hz, -CH_2_-aryl-*C2*), 117.15 (-*C*N), 117.10 (*C5*), 113.55 (-CH_2_-aryl-*C5*), 111.71 (*C8*), 102.00 (*C9*), 84.50 (*C4′*), 80.08 (*C1′*), 77.88 (*C3′*), 77.80 (*C2′*), 71.91 (-*C*H_2_-aryl), 71.42 (-O*C*H_2_CH_2_(CH_2_)_11_-), 71.04 (*C1″*), 65.38 (*C3″*), 65.01 (*C5′*), 56.01 (-O*C*H_3_), 32.41 (-OCH_2_*C*H_2_(CH_2_)_11_-), 0.46–29.45 (m, -OCH_2_CH_2_(*C*H_2_)_11_-), 26.58 (-*C*H_2_CH_2_CH_3_), 23.08 (-CH_2_*C*H_2_CH_3_, 13.82. (-*C*H_3_). HRMS (ESI) *m*/*z* [M+H]^+^ calcd for C_39_H_60_FN_5_O_10_P 808.4056, found 808.4049. HPLC 99.8%.

#### (*S*)-2-(Benzyloxy)-3-(tetradecyloxy)propan-1-ol, **4a**

Following Method C, **4a-MMTr** (5.9 g, 9 mmol) was deprotected with p-TsOH in CHCl_3_/MeOH (50 mL) to yield **4a** (1.57 g, 46%). ESI-MS *m*/*z* [M+Na]^+^ 401.51.

#### (*R*)-2-(Benzyloxy)-3-(tetradecyloxy)propyl dihydrogen phosphate, **8a**

Following General Method D2, a solution of alcohol **4a** (600 mg, 1.58 mmol) and triethylamine (400 mg, 3.95 mmol) was added to a solution of POCl_3_ (411 mg, 2.67 mmol) in CH_2_Cl_2_ (20 mL). After hydrolysis, phosphate **8a** was isolated (666 mg, 92%). ESI-MS *m*/*z* [M–H]^−^ 457.42.

#### ((3a*R*,4*R*,6*R*,6a*R*)-6-(4-aminopyrrolo[2,1-*f*][1,2,4]triazin-7-yl)-6-cyano-2,2-dimethyltetrahydrofuro[3,4-*d*][1,3]dioxol-4-yl)methyl ((*R*)-2-(benzyloxy)-3-(tetradecyloxy)propyl) hydrogen phosphate, **12a-acetonide**

Following General Method E, phosphate **8a** (720 mg, 1.57 mmol) was coupled to RVn-acetonide (520 mg, 1.57 mmol) using DIC (400 mg, 3.14 mmol) and NMI (390 mg, 4.71 mmol) in pyridine (20 mL). Purification yielded **12a-acetonide** (410 mg, 34%). ESI-MS *m*/*z* [M–H]^−^ 770.50.

#### ((2*R*,3*S*,4*R*,5*R*)-5-(4-Aminopyrrolo[2,1-*f*][1,2,4]triazin-7-yl)-5-cyano-3,4-dihydroxytetrahydrofuran-2-yl)methyl ((*R*)-2-(benzyloxy)-3-(tetradecyloxy)propyl) hydrogen phosphate, **12a**

Following General Method F, **12a-acetonide** (410 mg, 0.53 mmol) was added to formic acid (20 mL) and stirred 4h. Compound **12a** (210 mg, 54%) was isolated as an off-white solid. ^1^H NMR (400 MHz, CD_3_OD + CDCl_3_) δ 7.81 (s, 1H, *H2*), 7.34–7.16 (m, 5H, -CH_2_-*aryl*), 6.95 (d, *J* = 4.6 Hz, 1H, *H9*), 6.86 (d, *J* = 4.6 Hz, 1H, *H8*), 4.77 (d, *J* = 5.3 Hz, 1H, *H2′*), 4.62 (d, *J* = 11.9 Hz, 1H, -C*H*_2_-aryl), 4.57 (d, *J* = 11.8 Hz, 1H, -C*H*_2_-aryl), 4.35 (q, *J* = 4.4 Hz, 1H, *H4′*), 4.22 (t, *J* = 5.5 Hz, 1H, *H3′*), 4.14 (dt, *J* = 11.3, 4.0 Hz, 1H, *H5′*), 4.06 (q, *J* = 5.4 Hz, 1H, *H5′*), 3.89 (dp, *J* = 10.8, 5.7 Hz, 2H, *H3″*), 3.74–3.63 (m, 1H, *H2″*), 3.47 (qd, *J* = 10.6, 5.0 Hz, 2H, *H1″*), 3.37 (td, *J* = 6.6, 1.4 Hz, 2H, OC*H*_2_CH_2_(CH_2_)_11_-), 1.50 (p, *J* = 6.7 Hz, 2H, -OCH_2_C*H*_2_(CH_2_)_11_-), 1.25 (br s, 22H, -OCH_2_CH_2_(C*H*_2_)_11_-), 0.88 (t, 3H). ^13^C NMR (101 MHz, CD_3_OD+CDCl_3_) δ 156.30 (*C6*), 147.31 (*C2*), 138.94 (-CH_2_-aryl-*C1*), 128.45 (-CH_2_-aryl-*C2 + C6*), 128.11 (-CH_2_-aryl-*C3 + C5*), 127.76 (-CH_2_-aryl-*C4*), 124.96 (*C7*), 116.97 (-*C*N, *C5*), 111.57 (*C8*), 101.99 (*C9*), 84.25 (*C4′*), 84.23 (*C1′*), 79.96 (*C3′*), 77.80 (*C2′*), 77.73 (C2″), 72.32 (-*C*H_2_-aryl), 71.85 (-O*C*H_2_CH_2_(CH_2_)_11_-), 70.88 (*C1″*), 65.34 (*C3″*), 64.89 (*C5′*), 32.27 (-OCH_2_*C*H_2_(CH_2_)_9_-), 30.41–29.23 (m, -OCH_2_CH_2_CH_2_(*C*H_2_)_9_-), 26.43 (-OCH_2_CH_2_*C*H_2_(CH_2_)_9_-), 22.95 (-CH_2_*C*H_2_CH_3_), 13.81 (-*C*H_3_). HRMS (ESI) *m*/*z* [M+H]^+^calcd for C_36_H_55_N_5_O_9_P 732.3732, found 732.3722. HPLC purity 99.0%.

### Cells

Vero-TMPRSS2 cells (Sekisui XenoTech) were grown in DMEM plus 10% FBS, 1× penicillin/streptomycin, and 1 mg/mL geneticin at 37 °C and 5% CO_2_. Calu-3 cells (ATCC #HTB-55) were grown in MEM plus 10% FBS, 1× penicillin/streptomycin, 1 mM sodium pyruvate, and l-glutamine or GlutaMAX at 37 °C, 5% CO2. Huh7.5 cells (Apath LLC) were grown in DMEM plus 10% FBS, 1× penicillin/streptomycin. For human embryonic stem cell and induced pluripotent stem cell-derived lung organoid generation, 3D and monolayer lung organoids were generated and adapted into monolayers for infection experiments as previously described.^34^ H9 embryonic stem cells (WiCell) and ALDA31616 (iPSCs) were cultured in Matrigel (Corning #354230)-coated plates in mTeSR medium (StemCellTech #85850). The human stem cells (SC) were passaged using ReLeSR (Stem Cell Tech #05872) and maintained in an undifferentiated state, in a 5% CO2 incubator at 37 °C. For lung organoid generation, human SCs were dissociated with accutase for 20 min and then seeded onto Matrigel-coated plates at a density of 5.3 × 10^4^ cells/cm^2^ in mTeSR and 10 μM ROCK inhibitor Y-27632 (R&D Systems) for 24h. Day 1 of differentiation was induced when cells reached 50–70% confluency using Definitive Endoderm (DE) induction medium (RPMI1640, 2% B27 supplement, 1% HEPES, 1% glutamax, 50 U/mL penicillin/streptomycin) supplemented with 100 ng/mL human activin A (R&D) and 5 μM CHIR99021 (Stemgent). 20 to 24h after initial DE induction, media was changed to DE media supplemented with 100 ng/mL human activin A on days 2 and 3. Anterior Foregut Endoderm (AFE) induction media was added on days 4–6, and consisted of serum-free basal medium (3 parts IMDM:1 part F12, 1% B27+ 0.5% N2 supplements, 50 U/mL penicillin/streptomycin, 0.25% BSA, 0.05 mg/mL l-ascorbic acid, 0.4 mM monothioglycerol) supplemented with 10 μM SB431542 (R&D) and 2 μM Dorsomorphin (StemGent). On day 7, AFE monolayer cells were passaged using accutase for 10 min and then 3.0 × 10^5^ cells embedded into 150 μL of cold, undiluted Matrigel droplets in a 12-well plate. The Matrigel was allowed to gel in the 37 °C incubator for 30 min. Then 1.5 mL of Lung Progenitor Cell (LPC) induction medium, supplemented with 10 μM ROCK inhibitor Y-27632 the day of passage, was added to submerge the 3D Matrigel droplets. LPCs were cultured for a total of 9–11 days, with every other day media changes. The media consisted of serum-free basal medium supplemented with 10 ng/mL human recombinant BMP4 (R&D), 0.1 μM all-trans retinoic acid (Sigma-Aldrich), and 3 μM CHIR99021. To generate 3D human proximal lung organoids, we used a modified published protocol.^35^ Three-dimensional LPCs were resuspended and then dissociated in Dispase for 30–45 min to remove the Matrigel. Cold PBS was added to depolymerize the Matrigel, organoids were transferred to conical tubes and then centrifuged at 400*g* for 5 min. The supernatant was carefully removed, and a second cold PBS wash was performed. The supernatant was carefully removed and the cell/Matrigel mixture was resuspended in 3 mL of TrypLE Express (Gibco # 12605010) for 20 min at 37 °C. The reaction was quenched and resuspended with 2% FBS in DMEM/F12 then centrifuged at 400*g* for 5 min. After centrifugation, the supernatant was removed and the cell pellet was resuspended in 1 mL of quenching media supplemented with 10 μM Rock inhibitor (Y-27632). Cells were counted, aliquoted, and then centrifuged. The cell pellets were resuspended in pure cold Matrigel and placed onto a 12-well, 0.4 μm pore size Transwell (Corning) culture insert at 5.0 × 10^4^ cells per 200 μL of Matrigel. The Matrigel was allowed to gel in the incubator for 30 min and proximal lung organoid media was added to the basolateral side of the transwell. Proximal lung organoid media consisted of serum-free basal media supplemented with 250 ng/mL FGF2, 100 ng/mL rhFGF10, 50 nM dexamethasone (Dex), 100 μM 8-bromoadenosine 3′,5′-cyclic monophosphate sodium salt (Br-cAMP), 100 μM 3-isobutyl-1-methylxanthine (IBMX), and 10 μM ROCK inhibitor (Y-27632). Proximal lung organoids were cultured for 2 weeks with the media changed every other day. For monolayer experiments, the 3D proximal organoids were dissociated into single cells per the LPC using the organoid passaging protocol above and seeded at 20,000 cells per well onto Matrigel-coated 96-well plates 3 days before infection in 100 μL of proximal medium.

### Viruses

SARS-CoV-2 variants WA1 (USA-WA1/2020, BEI NR-52281), B.1.351/Beta (hCoV-19/South Africa/KRISP-K005325/2020, BEI NR-54009), and P.1/Gamma (hCoV-19/Japan/TY7-503/2021, BEI NR-54982) were acquired from BEI. WA1 and B.1.351 were passaged once through primary human bronchial epithelial cells (NHBECs) differentiated at the air–liquid interface (ALI). B.1.1.7/Alpha isolate hCoV-19/USA/CA_UCSD_5574/2020 was isolated on ALI from a nasopharyngeal swab obtained by Dr. Louise Laurent under UCSD IRB #200477 with sequence deposited at GISAID (EPI_ISL_751801). BA.1.20/Omicron isolate hCoV-19/USA/CA-SEARCH-59467/2021 was isolated on Vero-TMPRSS2 cells from a nasopharyngeal swab obtained from the UC San Diego CALM and EXCITE laboratories under UC San Diego IRB #160524 with sequence deposited at GISAID (EPI_ISL_8186377). All viruses were expanded on Vero-TMPRSS2 cells. Supernatants were clarified and stored at −80 °C, and titers were determined by fluorescent focus assay on Vero-TMPRSS2 cells and by TCID50 on Calu-3 cells. Viral stocks were verified by whole-genome sequencing. *In vitro* work with SARS-CoV-2 was conducted in Biosafety Level-3 conditions at UC San Diego following the guidelines approved by the Institutional Biosafety Committee. For mouse experiments, a stock of SARS-CoV-2 MA10, a mouse-adapted virulent mutant generated from a recombinantly derived synthesized sequence of the Washington strain, were generated in the Baric lab. Virus was maintained at low passage (P2–P3) to prevent the accumulation of additional potentially confounding mutations.

### Antiviral Cell-Based Assays

Cells were seeded in black-walled 96-well plates as follows: 20,000 Huh7.5 cells per well the day before infection, 20,000 Calu-3 cells per well 2–3 days before infection, 12,000–15,000 Vero-TMPRSS2 cells per well the day before infection, and 20,000 iPSC-lung cells per well on Matrigel-coated plates 3 days before infection. Duplicate wells were pretreated with 3-fold serial dilutions of compounds or DMSO for 30–60 min before addition of SARS-CoV-2 WA1. Multiplicities of infection (MOIs) were 0.01 FFU/cell for Calu-3, Vero-TMPRSS2, and iPSC-lung cells and 0.1 for Huh7.5 cells. For SARS-CoV-2 variant experiments, Calu-3 cells were infected with approximately 350 TCID_50_ (MOI = 0.018) of WA1, B.1 (D614G), B.1.1.7 (Alpha), B.1.315 (Beta), P.1 (Gamma), B.1.617.2 (Delta), and with 2000 TCID_50_ (MOI = 0.1) of BA.1.20 (early Omicron). In all experiments, compounds and virus remained on cells for the duration of the assay, and cells were fixed with 4–4.5% formaldehyde for at least 30 min at rt at 48 hours post-infection (hpi) for Huh7.5 cells, 40–44 hpi for Calu-3 cells, 30–32 hpi for Vero-TMPRSS2 cells, and 24 hpi for iPSC-lung cells. Fixed cells were stained with antibody against nucleocapsid (GeneTex, Cat. #gtx135357) with Sytox Green nuclear counterstain and imaged in Incucyte S3 or SX5. Infected cells, total nuclei, and percent infected cells were detected by the Incucyte automated analysis software. Percent infected cells values were normalized to DMSO controls on each plate, and best-fit curves were calculated in GraphPad Prism 9.

### Cell Viability Assay

Cells were seeded as per SARS-CoV-2 infection studies in opaque walled 96-well cell culture plates and incubated overnight for Huh7.5 and Vero-TMPRSS2 cells and for an additional 24h for Calu-3 cells. Compounds or controls were added at the indicated concentrations. Cells were incubated for 48h for Huh7.5 cells, 40–44h for Calu-3 cells, or 30–32h for Vero-TMPRSS2 cells at 37 °C and 5% CO_2_, an equal volume of CellTiter-Glo reagent (Cat. #G7570, Promega) or CellTiter-Glo 2.0 reagent (Cat. #G9241, Promega) was added and mixed, and luminescence was recorded on a Veritas Microplate Luminometer (Turner BioSystems) according to manufacturer recommendations. Percent viability was calculated compared to DMSO controls, and CC_50_ values were calculated using Prism 9.

### SARS-CoV-2 Infection Model in Mice

This study was conducted under UNC IACUC approval (20-114). Young (∼10- to 12-week-old) BALB/c mice (100% female) were obtained from Envigo. Animals were acclimated for 7 days in the BSL-3 prior to any experimentation and were housed 4/cage with food and water provided *ad libitum*, with a 12/12 light/dark cycle. Animals were anesthetized i.p. with a combination of 50 mg/kg ketamine and 15 mg/kg xylazine in 50 μL. Animals were infected intranasally with 10^4^ PFU of sequence- and titer-verified SARS-CoV-2 MA10 in 50 μL of DMEM diluted in PBS to the inoculation dosage. V2043 was evaluated at two dosages (30 mg/kg and 60 mg/kg) and two dosing schedules (PO, QD and PO, BID). Formulations were administered to mice starting at +12 hpi or +24 hpi. Subsequent doses were administered QD or BID (see table below) at approximately the same times each day post-infection. Administration was PO for all experimental groups. Daily clinical evaluation and scoring, including body weight and disease score were conducted. Animals were carried to the experimental endpoints of 2 and 5 days post-infection. Fifty animals, 10 in each drug evaluation group and 10 in the mock control group, were evaluated in each treatment condition, with 5 animals intended for each experimental endpoint. No animals were sacrificed before the experimental time points. Euthanasia was performed by inhalational of isoflurane (drop method) and thoracotomy, with removal of vital organs (lungs). Lung tissue was taken for assessments of titer and histology. Statistical analysis of weight loss data was performed in GraphPad Prism 9. Statistical analyses varied by assay and are indicated with each experiment when applicable.

## Supporting Information Available

NMR spectroscopy data (^1^H, ^13^C, ^31^P), HRMS, and HPLC for all tested compounds, Figure S1 showing cytotoxicity in the various cell types, and Table S1 comparing V2043, GS-621763 and molnupiravir mouse model results (PDF)
Molecular formula strings (CSV)

## Abbreviations Used

SARS-CoV-2: severe acute respiratory syndrome coronavirus 2
TLC: thin-layer chromatography
