# Supplementary material for: 1-O-Octadecyl-2-O-benzyl-sn-glyceryl-3-phospho-GS-441524 (V2043). Evaluation of Oral V2043 in a Mouse Model of SARS-CoV-2 Infection and Synthesis and Antiviral Evaluation of Additional Phospholipid Esters with Enhanced Anti-SARS-CoV-2 Activity
Source: J Med Chem. 2023 Apr 11;66(8):5802–19. doi: 10.1021/acs.jmedchem.3c00046 (PMC10108740; doi:10.1021/acs.jmedchem.3c00046)
Supplement: Supplementary file 1 — jm3c00046_si_001.pdf [file jm3c00046_si_001.pdf]

SUPPORTING INFORMATION

1-*O*-Octadecyl-2-*O*-benzyl-*sn*-glyceryl-3-*phospho*-GS-441524 (V2043). Evaluation of Oral V2043 in a Mouse Model of SARS-CoV-2 Infection and Synthesis and Antiviral Evaluation of Additional Phospholipid Esters with Enhanced Anti-SARS-CoV-2 Activity

Aaron F. Carlin[1,2,#], James R. Beadle[1,#], Alex E. Clark[1,2], Kendra L. Gully[3], Fernando R. Moreira[3], Ralph S. Baric[3], Rachel L. Graham[3], Nadejda Valiaeva[1], Sandra L. Leibel[4], William Bray[4], Rachel E. McMillan[1,2], Jonathan E. Freshman[1,2], Aaron F. Garretson[1,2], Rachael N. McVicar[5], Tariq Rana[4], Xing-Quan Zhang[1], Joyce A. Murphy[1], Robert T. Schooley[1], Karl Y. Hostetler[1,*]

[1] Department of Medicine, University of California, San Diego, La Jolla, CA 92093 USA

[2] Department of Pathology, University of California, San Diego, La Jolla, CA 92093 USA

[3] Department of Epidemiology, Gillings School of Global Public Health, University of North Carolina at Chapel Hill, Chapel Hill, NC 27599 USA

[4] Department of Pediatrics, University of California, San Diego, La Jolla, CA 92093 USA
[5] Sanford Burnham Prebys Discovery Institute, La Jolla, CA 92093 USA

*Corresponding Author. Email: khostetler@health.ucsd.edu

Table of Contents

Compound 9b 3

Compound 9c 6

Compound 9d 9

Compound 9e 13

Compound 9f 16

Compound 9g 19

Compound 9h 21

Compound 9i 26

Compound 9j 30

Compound 9k 34

Compound 9l 37

Compound 10a 40

Compound 10g 44

Compound 11a 48

Compound 11g 52

Compound 12a 56

Figure S1. Relative cell viability in uninfected cells after treatment with V2043 and analogs 59

Table S1. Effect of V2043, GS-621763 or Molnupiravir given 12 or 24 hours postinfection on Lung Titers at Day 4 or 5 After SARS-CoV-2 Infection in Balb/c Mice 60

Compound 9b ((2*R*,3*S*,4*R*,5*R*)-5-(4-aminopyrrolo[2,1-*f*][1,2,4]triazin-7-yl)-5-cyano-3,4-dihydroxytetrahydrofuran-2-yl)methyl ((*S*)-2-(benzyloxy)-3-(octadecyloxy)propyl) hydrogen phosphate

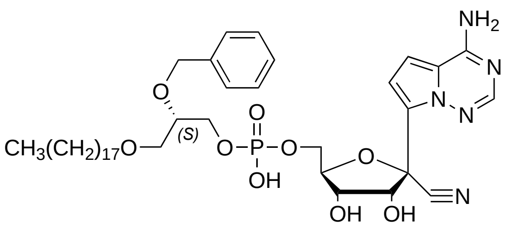

1. ^{1}H NMR

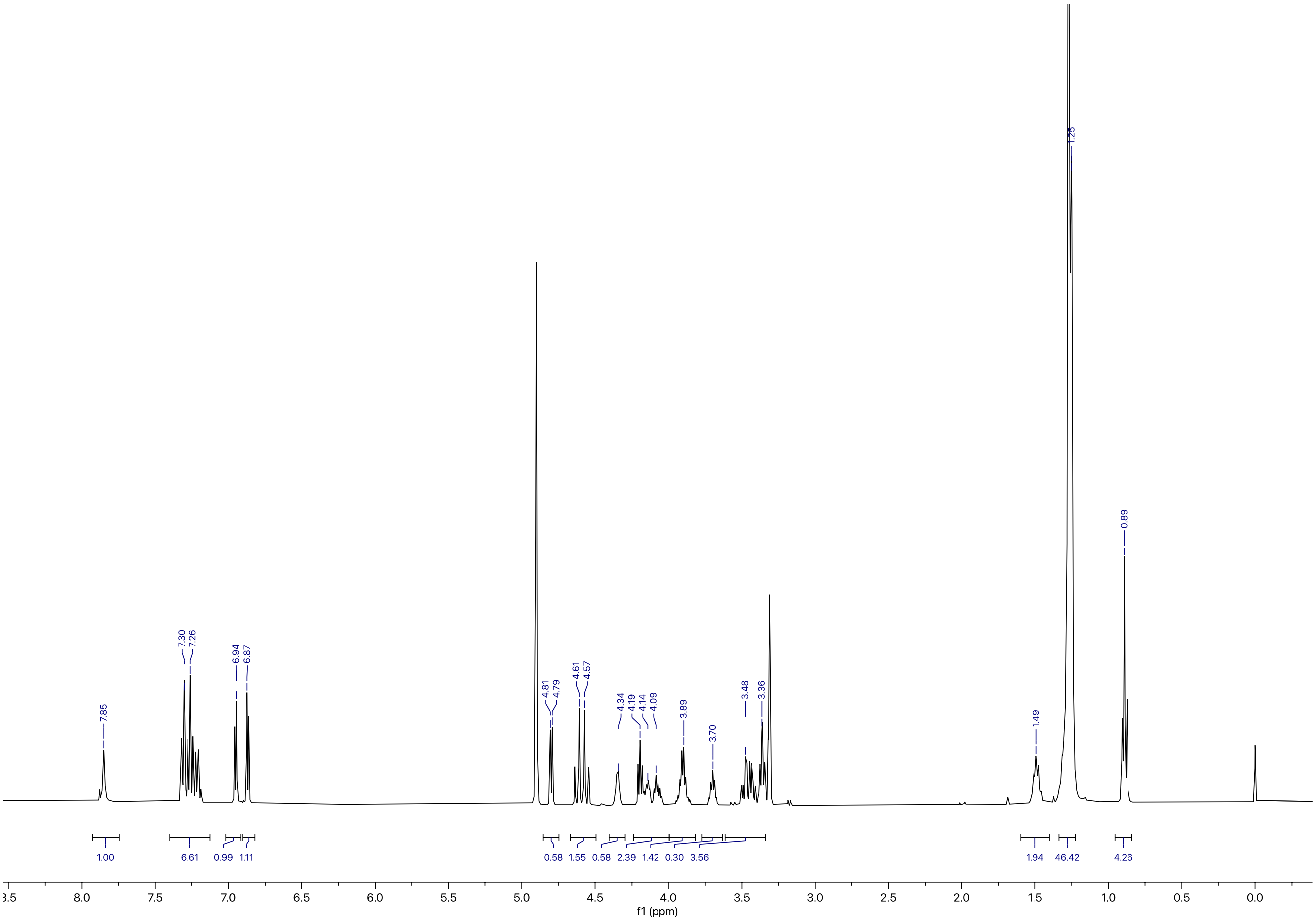

2. ^{13}C NMR

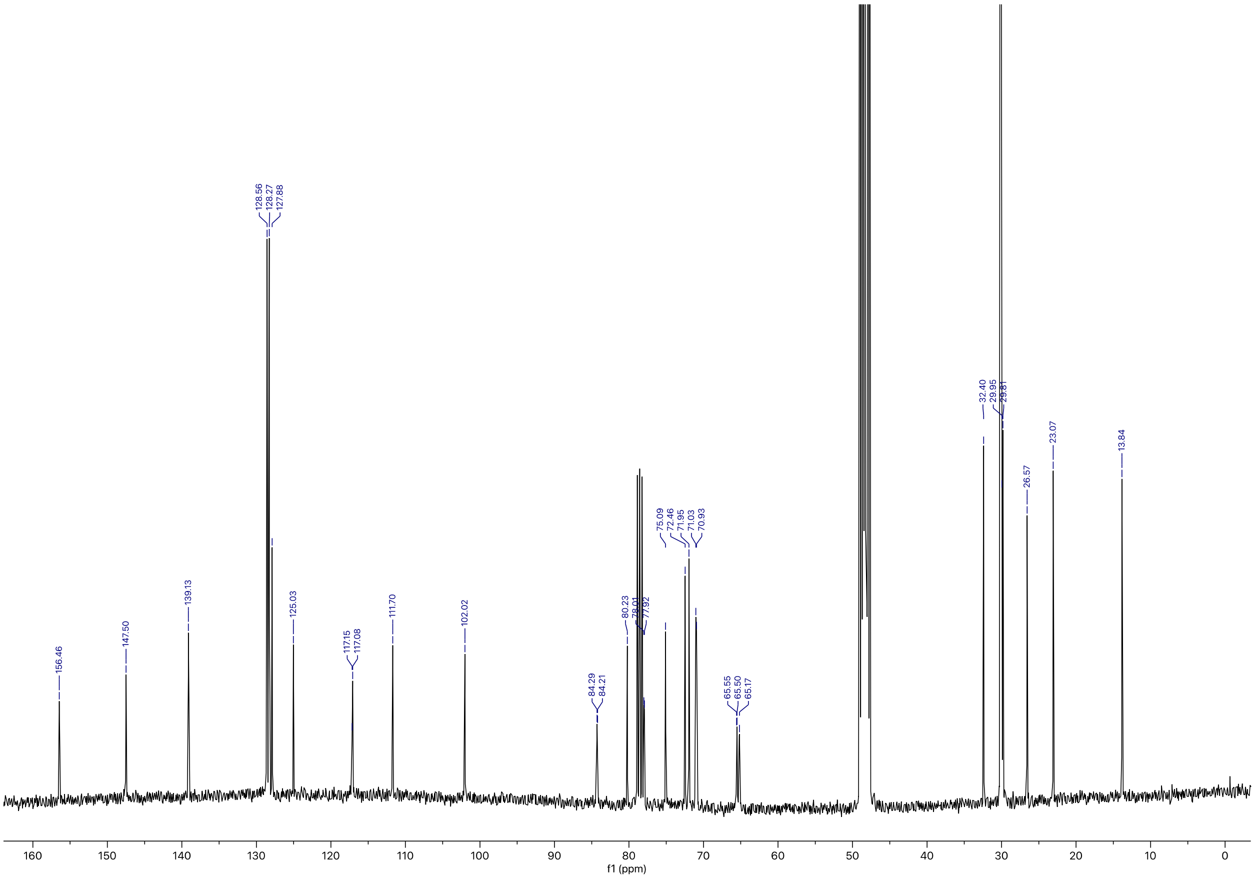

156.46
147.50
139.13
128.56
128.27
127.88
125.03
117.15
117.08
111.70
102.02
84.29
84.21
80.23
77.92
75.09
72.46
71.95
71.03
70.93
65.55
65.50
65.17
32.40
29.95
29.81
26.57
23.07
13.84
160
150
140
130
120
110
100
90
80
70
60
50
40
30
20
10
0
f1 (ppm)

3. HRMS

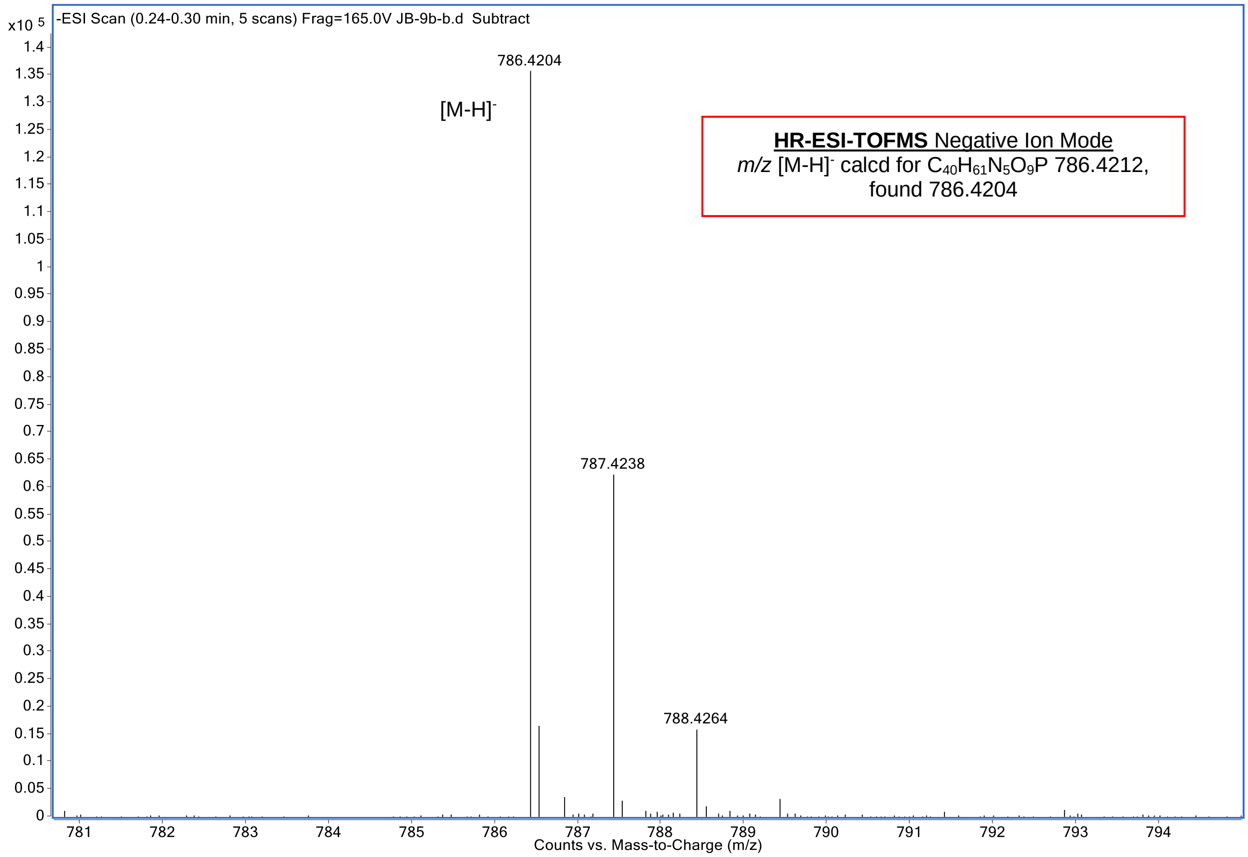

4. HPLC

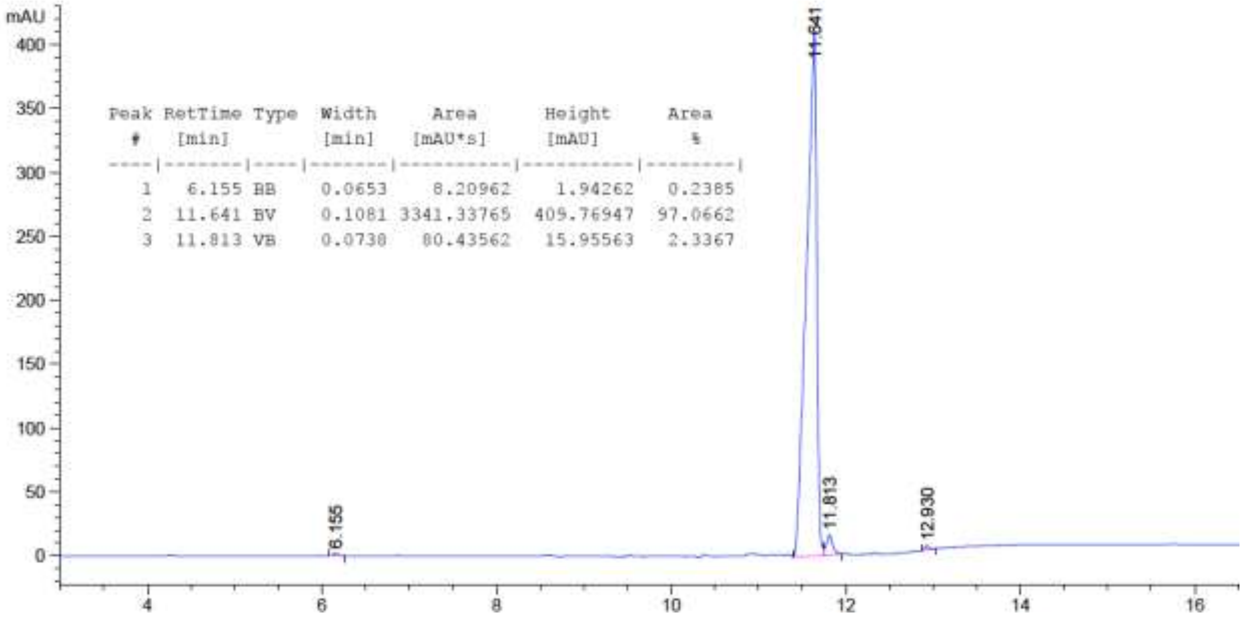

Peak #	RetTime [min]	Type	Width [min]	Area [mAU*s]	Height [mAU]	Area %
1	6.155	BB	0.0653	8.20962	1.94262	0.2385
2	11.641	BV	0.1081	3341.33765	409.76947	97.0662
3	11.813	VB	0.0738	80.43562	15.95563	2.3367

Compound 9c ((2*R*,3*S*,4*R*,5*R*)-5-(4-aminopyrrolo[2,1-*f*][1,2,4]triazin-7-yl)-5-cyano-3,4-dihydroxytetrahydrofuran-2-yl)methyl (2-(benzyloxy)-3-(octadecyloxy)propyl) hydrogen phosphate

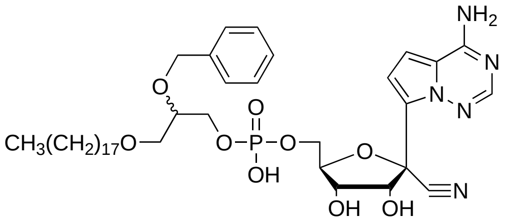

1. ^{1}H NMR

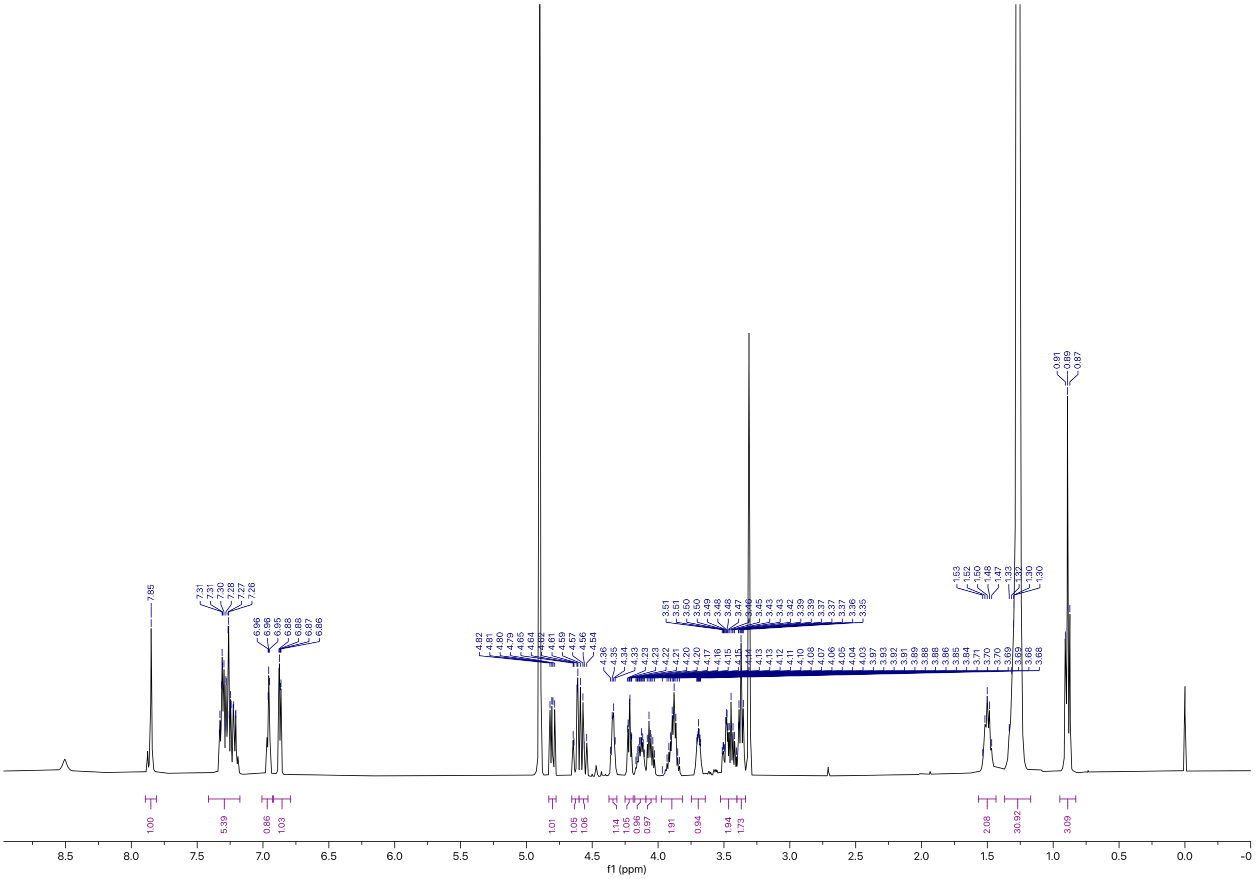

2. ^{13}C NMR

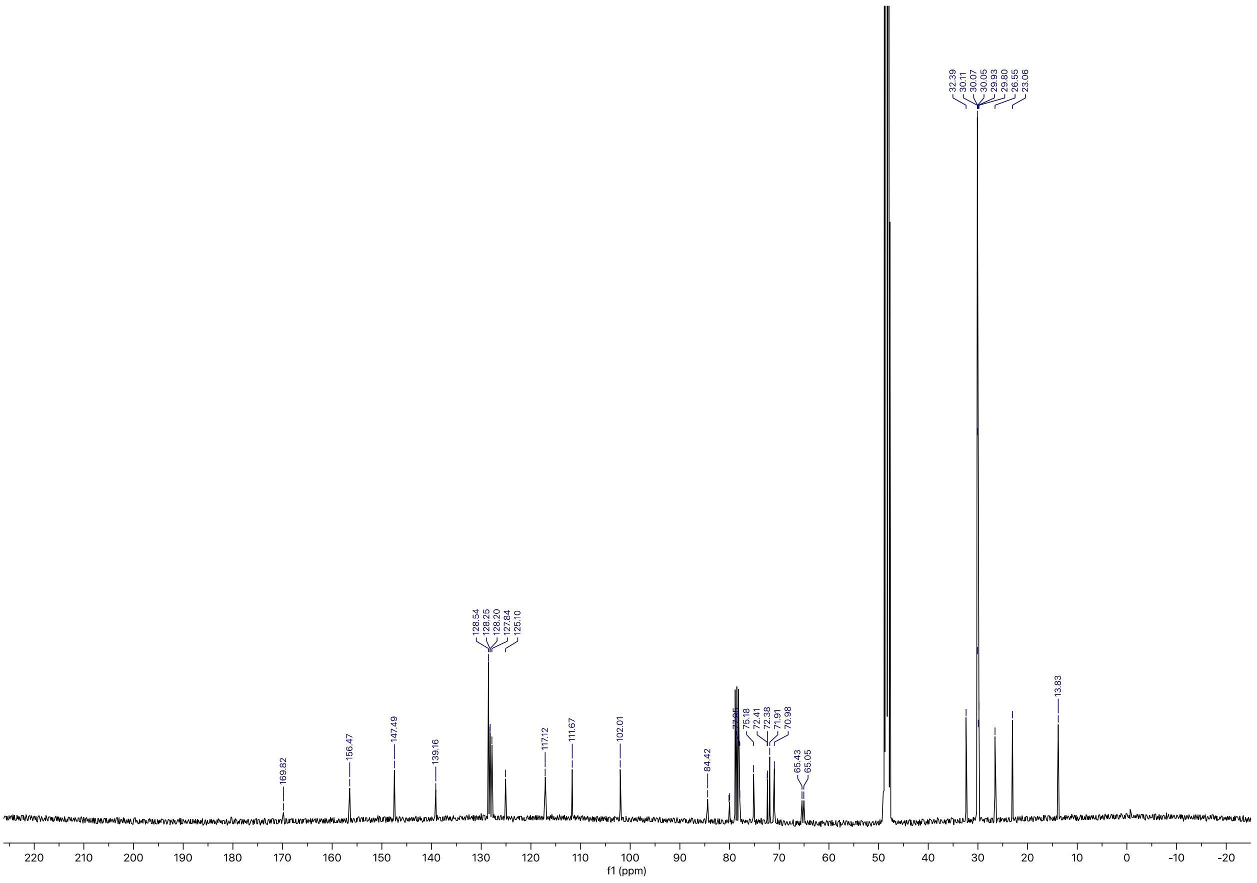

3. HRMS

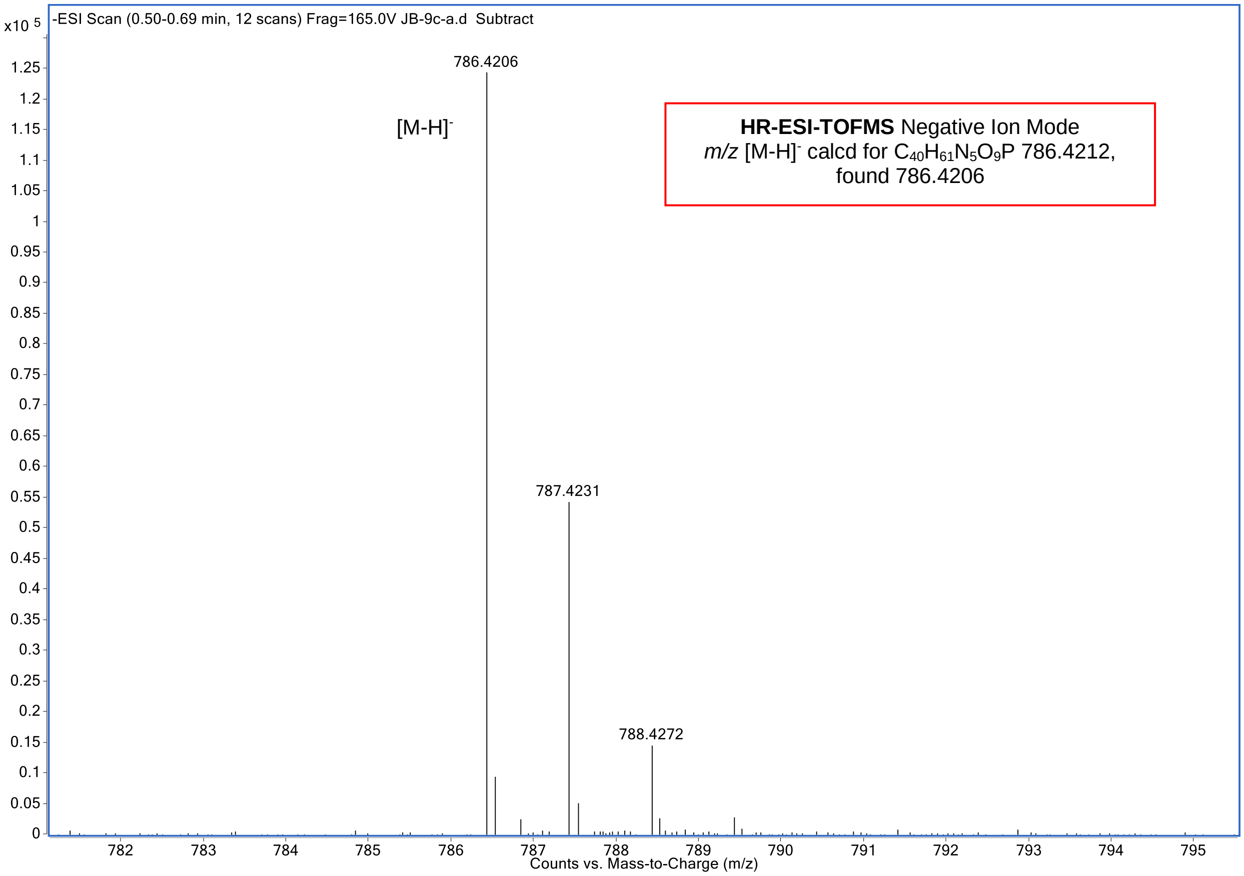

4. HPLC

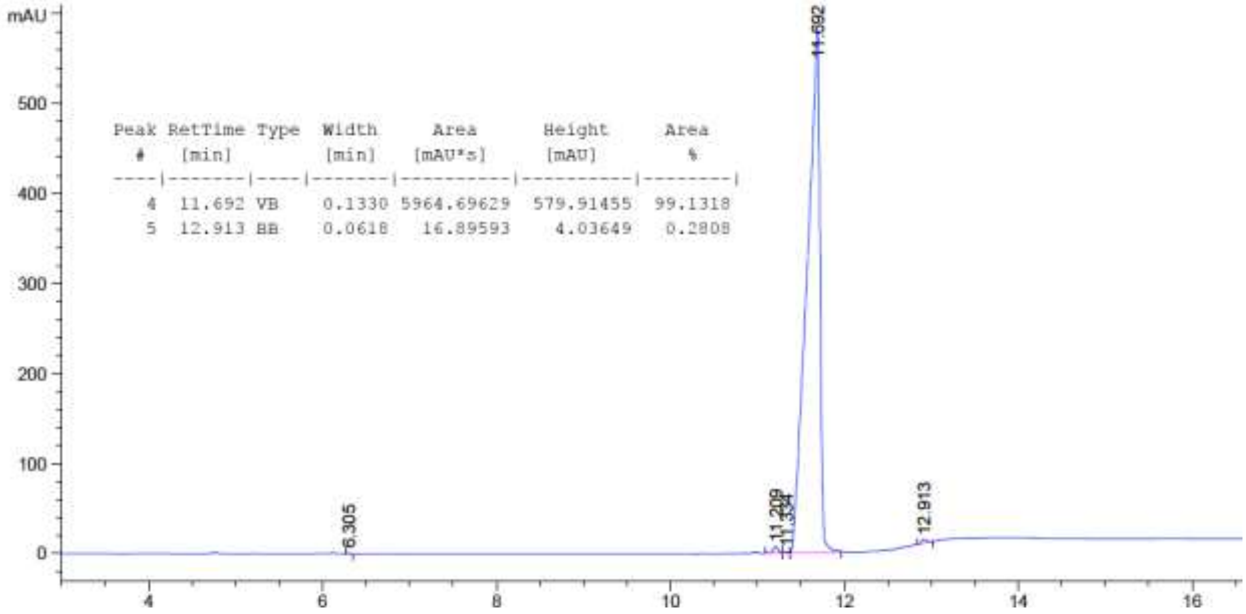

Peak #	RetTime [min]	Type	Width [min]	Area [mAU*s]	Height [mAU]	Area %
4	11.692	VB	0.1330	5964.69629	579.91455	99.1318
5	12.913	BB	0.0618	16.89593	4.03649	0.2808

Compound 9d ((2*R*,3*S*,4*R*,5*R*)-5-(4-aminopyrrolo[2,1-*f*][1,2,4]triazin-7-yl)-5-cyano-3,4-dihydroxytetrahydrofuran-2-yl)methyl ((*R*)-2-((3-fluorobenzyl)oxy)-3-(octadecyloxy)propyl) hydrogen phosphate

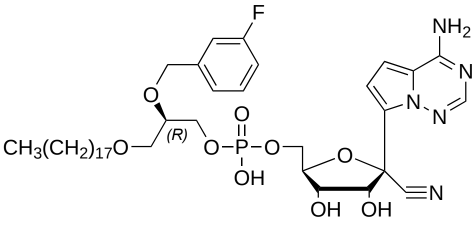

(Nanosyn)

1. ^{1}H NMR

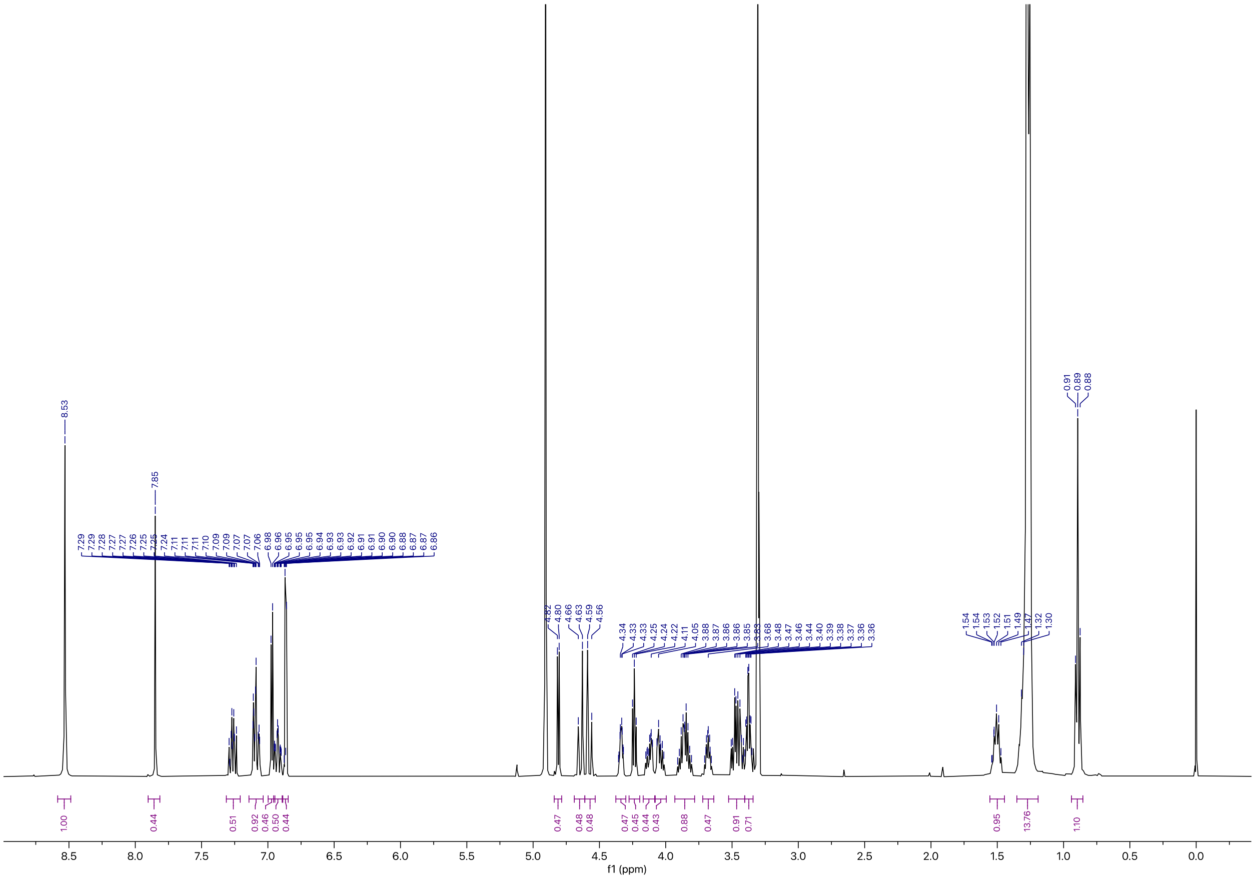

2. ^{13}C NMR

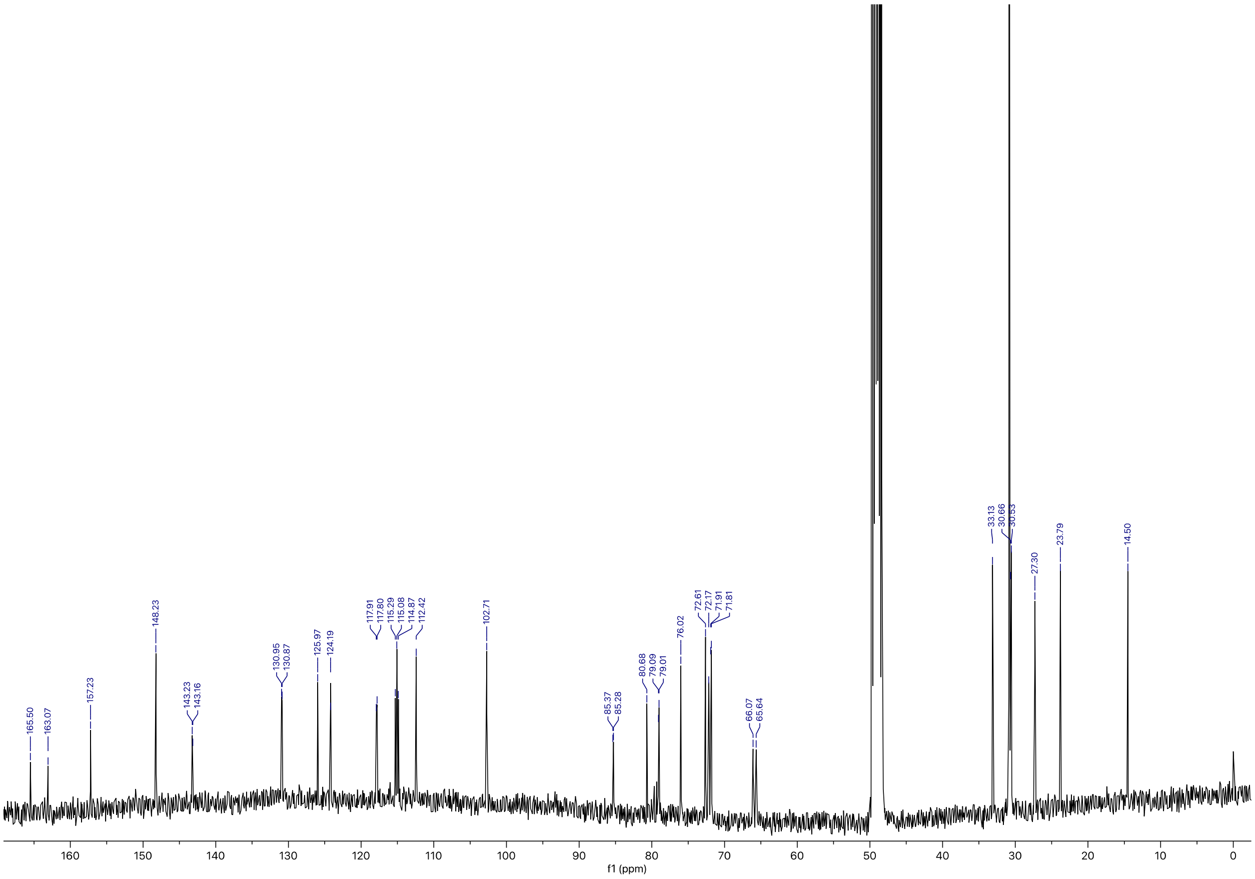

165.50
163.07
157.23
148.23
143.23
143.16
130.95
130.87
125.97
124.19
117.91
117.80
115.29
115.08
114.87
112.42
102.71
85.37
85.28
80.68
79.09
79.01
76.02
72.61
72.17
71.91
71.81
66.07
65.64
33.13
30.66
30.53
27.30
23.79
14.50
160
150
140
130
120
110
100
90
80
70
60
50
40
30
20
10
0
f1 (ppm)

3. HRMS

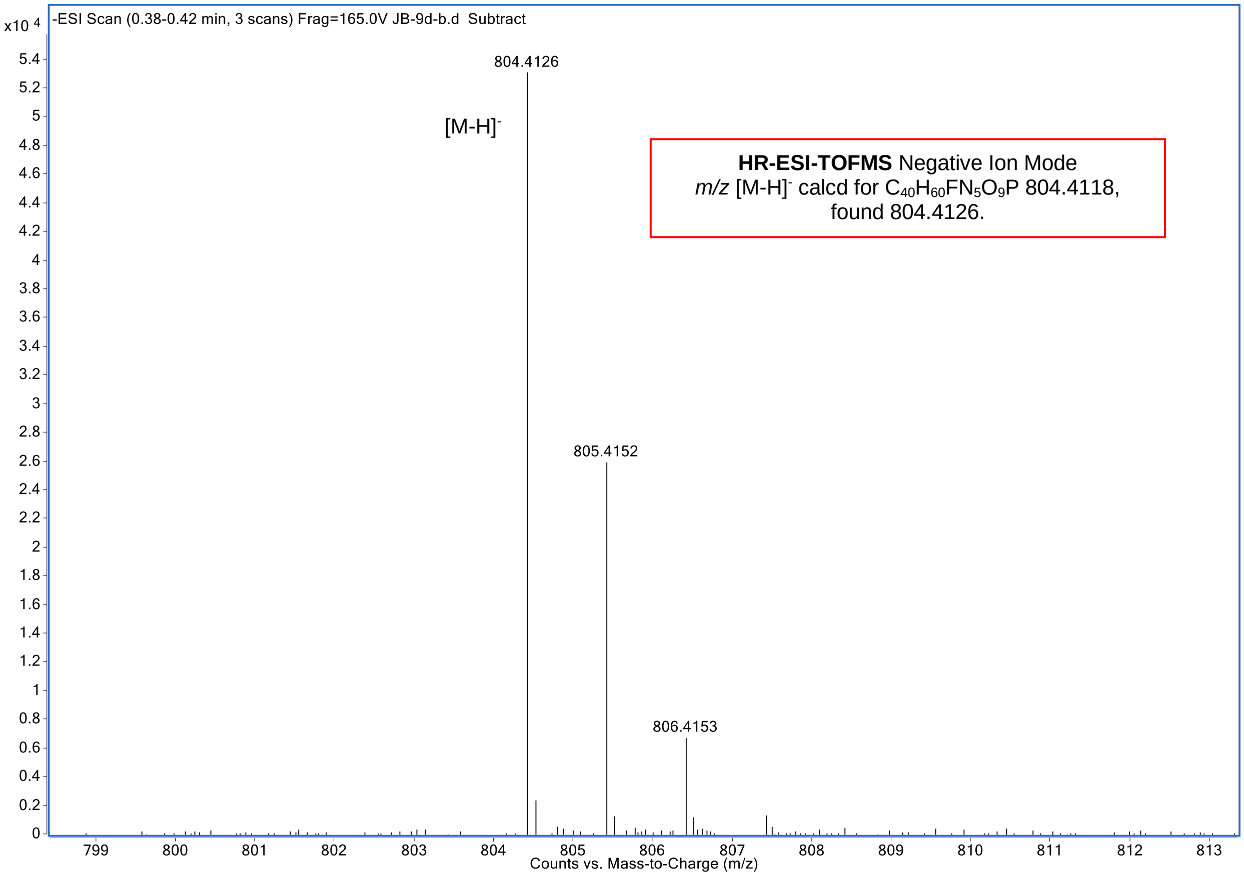

x10 4
-ESI Scan (0.38-0.42 min, 3 scans) Frag=165.0V JB-9d-b.d Subtract
804.4126
[M-H]-
HR-ESI-TOFMS Negative Ion Mode
m/z [M-H]- calcd for C40H60FN5O9P 804.4118,
found 804.4126.
805.4152
806.4153
5.4
5.2
5
4.8
4.6
4.4
4.2
4
3.8
3.6
3.4
3.2
3
2.8
2.6
2.4
2.2
2
1.8
1.6
1.4
1.2
1
0.8
0.6
0.4
0.2
0
799
800
801
802
803
804
805
806
807
808
809
810
811
812
813
Counts vs. Mass-to-Charge (m/z)

4. LCMS

ms filename	fmla Structure	Purity (%)	Weight (mg)	MW	NSID	Structure
093BP097	C40H61FN5O9P	98.7	41	805.419	NSN24772-093BP097_LCMS	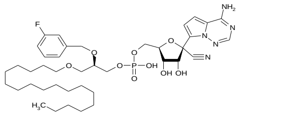

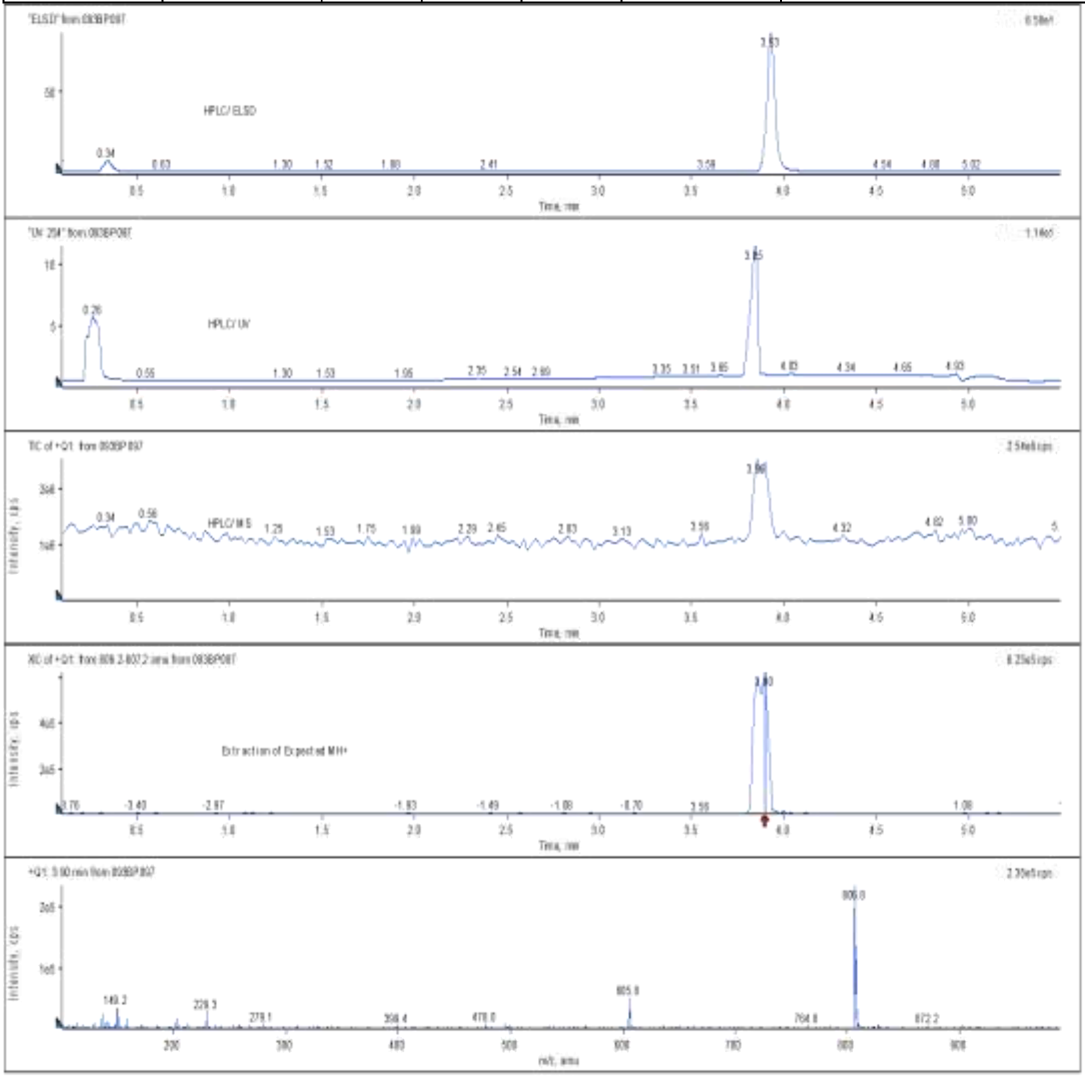

Compound 9e ((2*R*,3*S*,4*R*,5*R*)-5-(4-aminopyrrolo[2,1-*f*][1,2,4]triazin-7-yl)-5-cyano-3,4-dihydroxytetrahydrofuran-2-yl)methyl ((*R*)-3-(octadecyloxy)-2-((3-(trifluoromethyl)benzyl)oxy)propyl) hydrogen phosphate

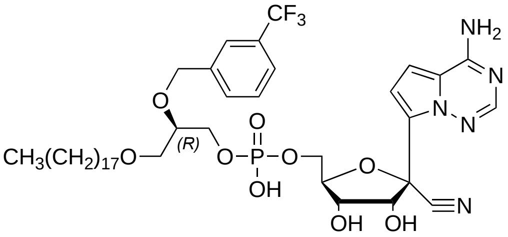

1. ^{1}H NMR

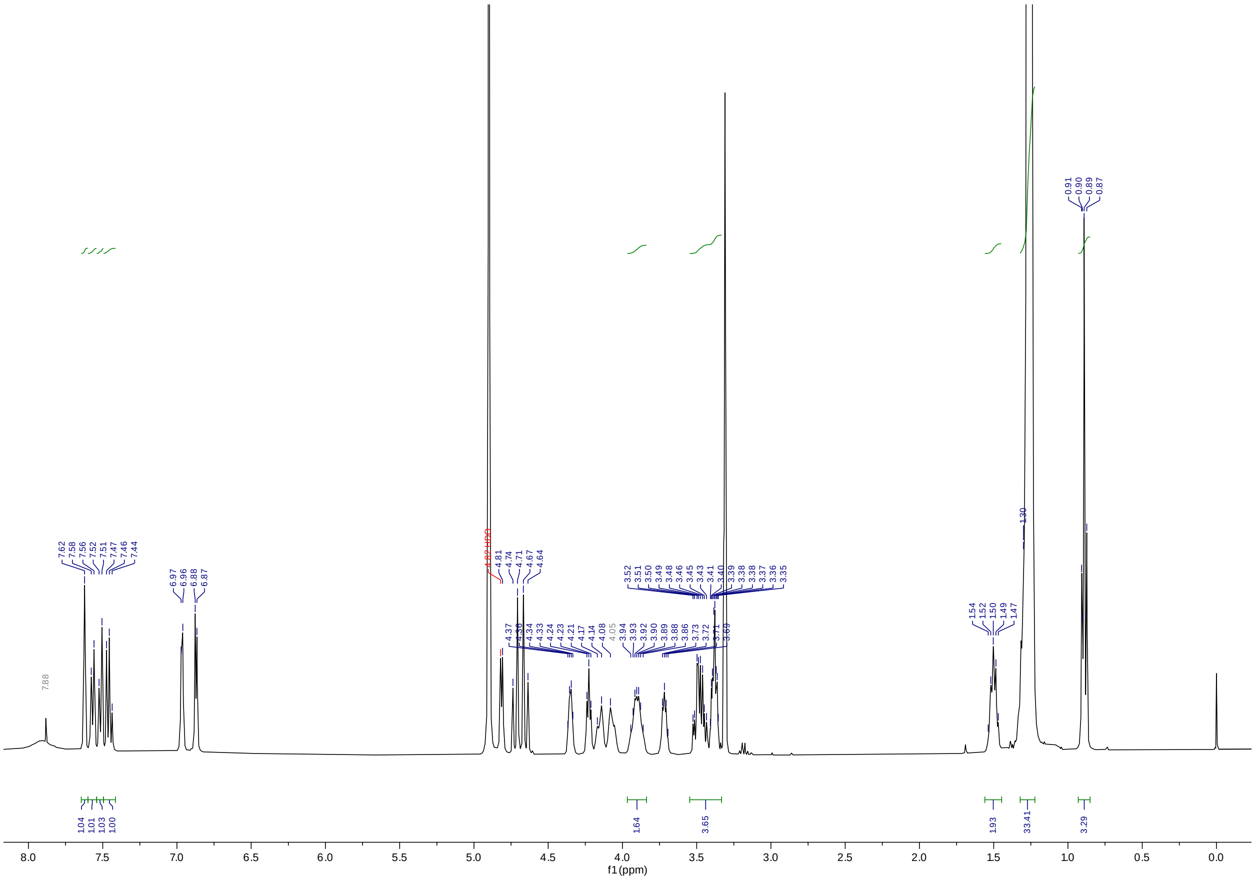

2. ^{13}C NMR

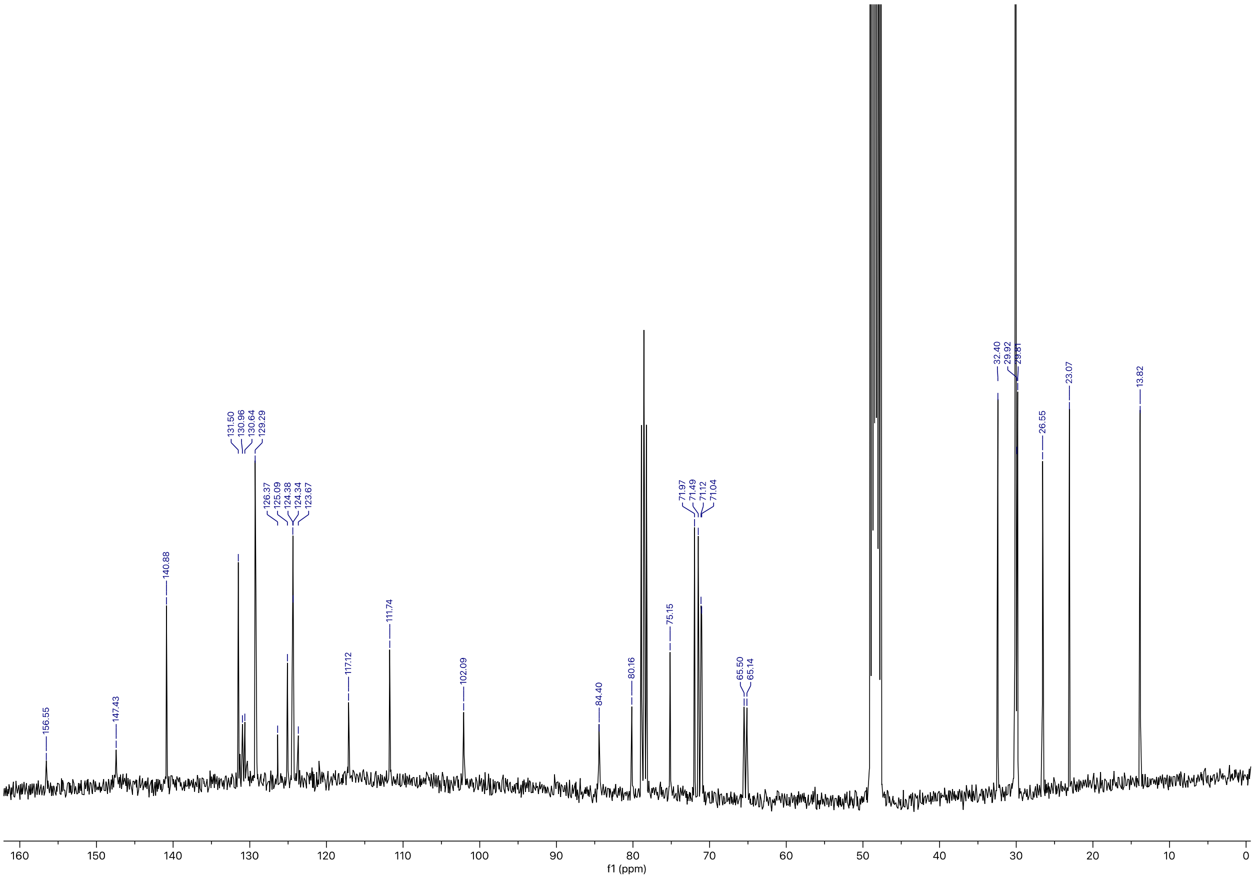

156.55
147.43
140.88
131.50
130.96
130.64
129.29
126.37
125.09
124.38
124.34
123.67
117.12
111.74
102.09
84.40
80.16
75.15
71.97
71.49
71.12
71.04
65.50
65.14
32.40
29.92
29.81
26.55
23.07
13.82
160
150
140
130
120
110
100
90
80
70
60
50
40
30
20
10
0
f1 (ppm)

3. HRMS

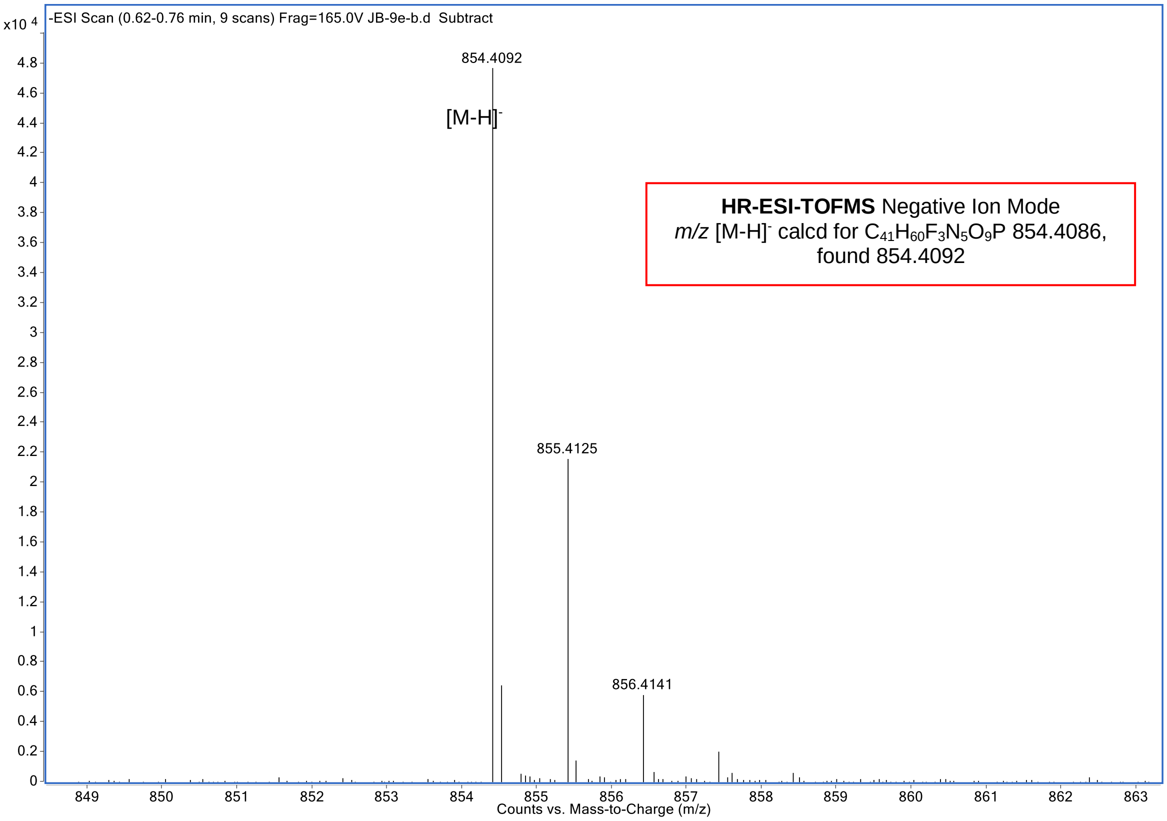

-ESI Scan (0.62-0.76 min, 9 scans) Frag=165.0V JB-9e-b.d Subtract
x10 4
854.4092
[M-H]-
855.4125
856.4141
HR-ESI-TOFMS Negative Ion Mode
m/z [M-H]- calcd for C41H60F3N5O9P 854.4086,
found 854.4092
Counts vs. Mass-to-Charge (m/z)

4. HPLC

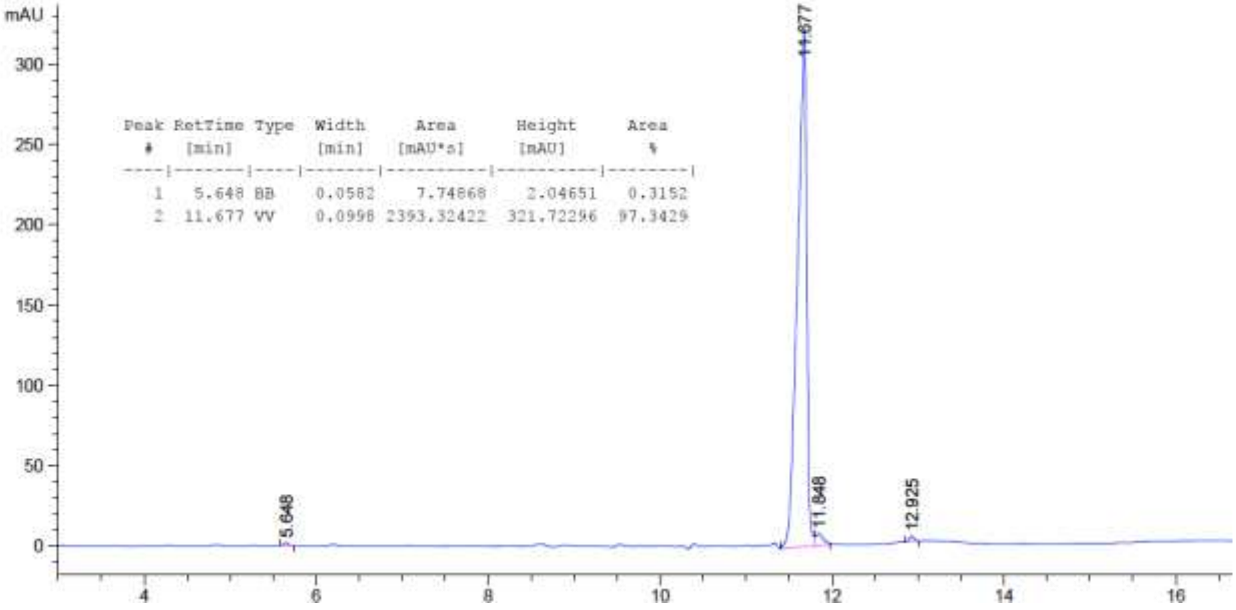

mAU
11.677
11.848
12.925
5.648
Peak # | RetTime [min] | Type | Width [min] | Area [mAU*s] | Height [mAU] | Area %
1 | 5.648 | BB | 0.0582 | 7.74868 | 2.04651 | 0.3152
2 | 11.677 | VV | 0.0998 | 2393.32422 | 321.72296 | 97.3429

Compound 9f ((2*R*,3*S*,4*R*,5*R*)-5-(4-aminopyrrolo[2,1-*f*][1,2,4]triazin-7-yl)-5-cyano-3,4-dihydroxytetrahydrofuran-2-yl)methyl ((*R*)-3-(octadecyloxy)-2-((4-(trifluoromethyl)benzyl)oxy)propyl) hydrogen phosphate

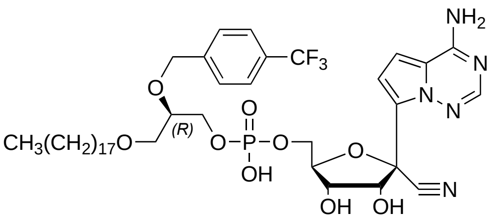

1. ^{1}H NMR

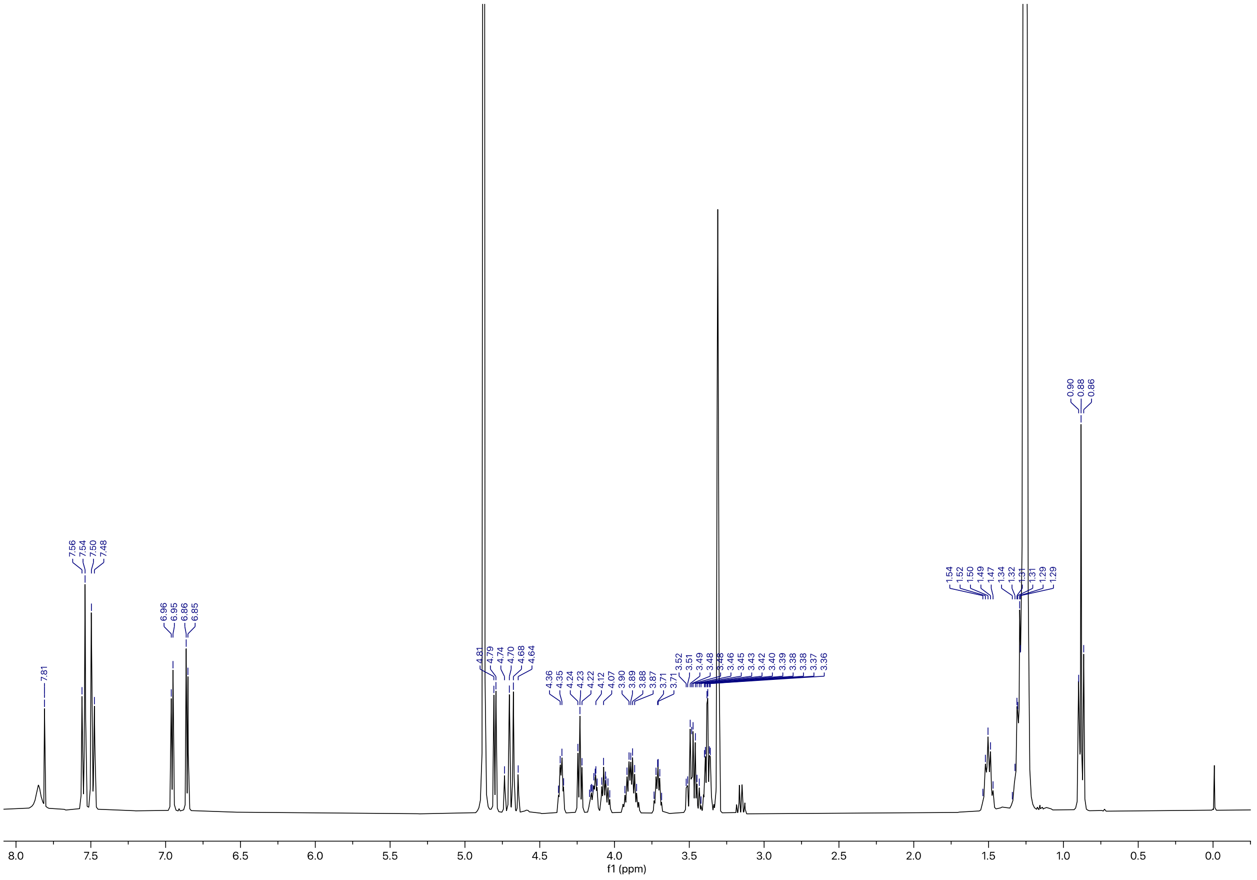

2. ^{13}C NMR

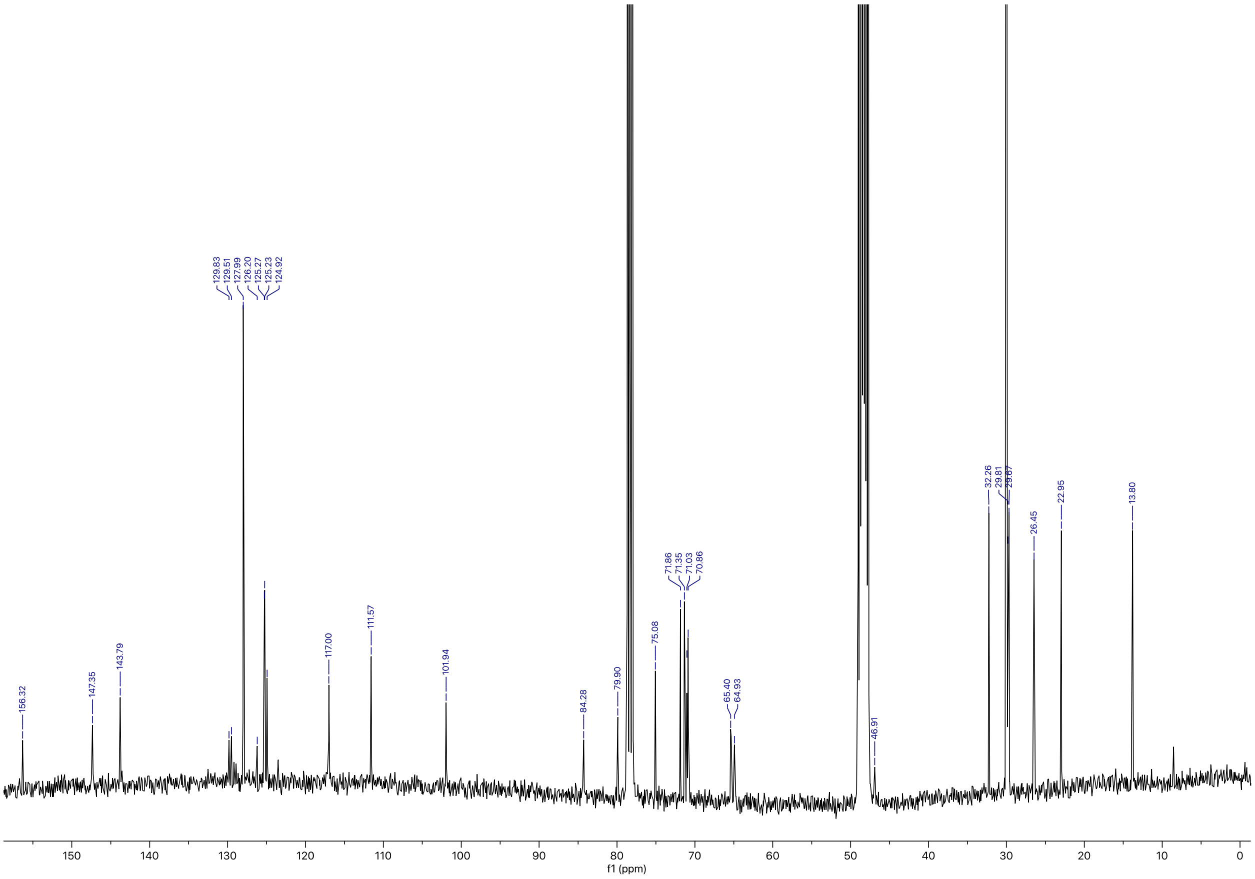

3. HRMS

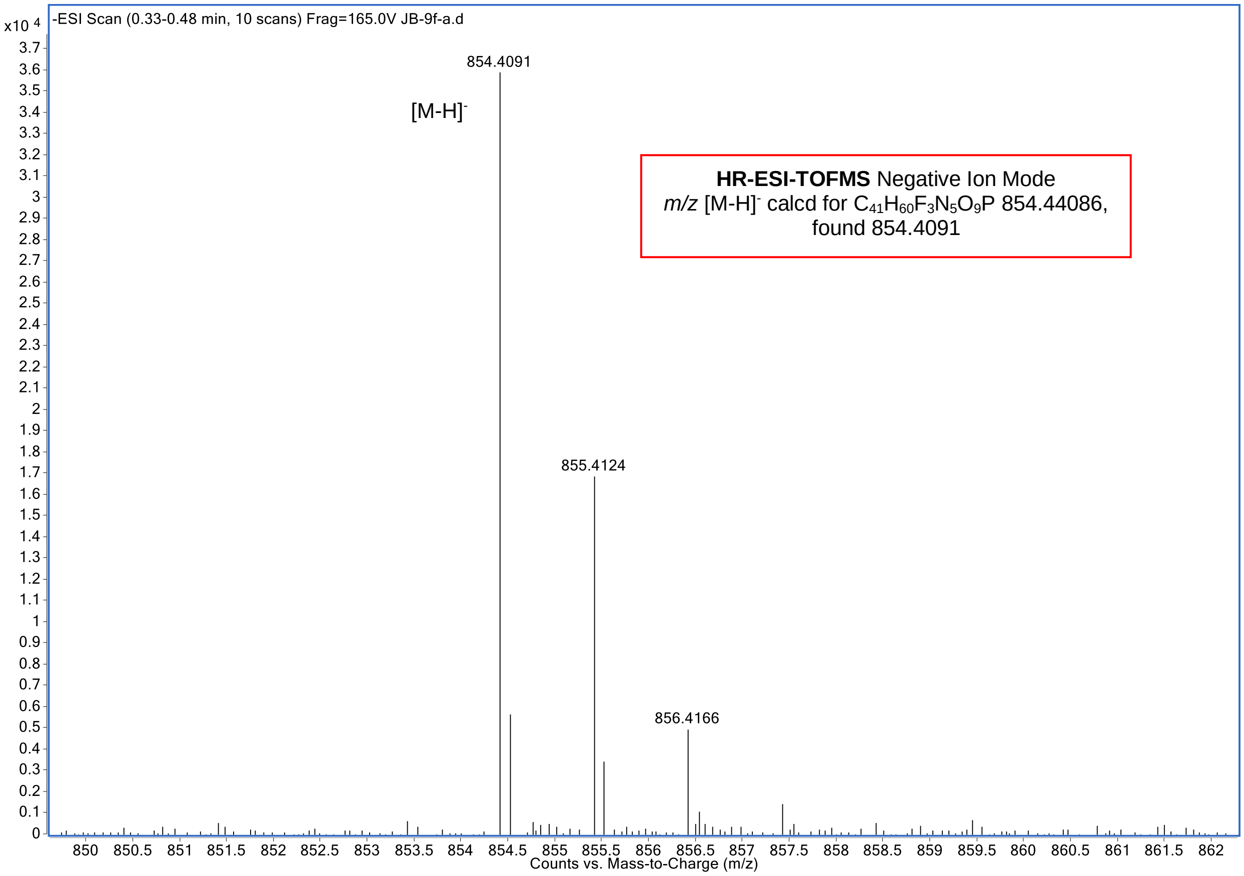

4. HPLC

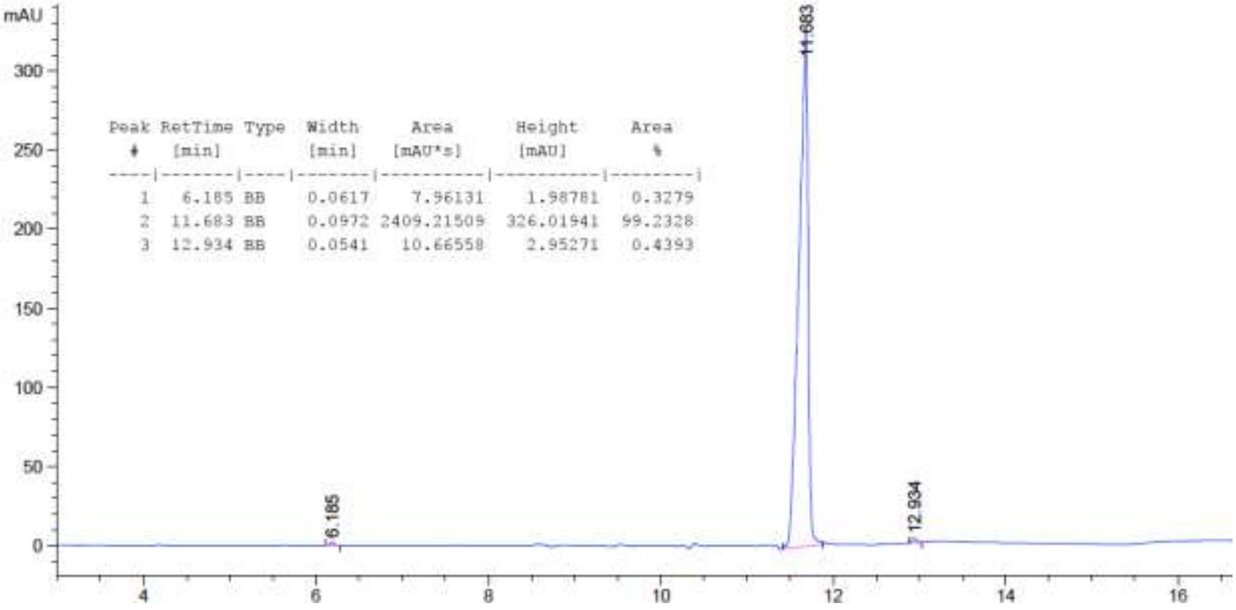

Peak #	RetTime [min]	Type	Width [min]	Area [mAU*s]	Height [mAU]	Area %
1	6.185	BB	0.0617	7.96131	1.98781	0.3279
2	11.683	BB	0.0972	2409.21509	326.01941	99.2328
3	12.934	BB	0.0541	10.66558	2.95271	0.4393

Compound 9g ((2*R*,3*S*,4*R*,5*R*)-5-(4-aminopyrrolo[2,1-*f*][1,2,4]triazin-7-yl)-5-cyano-3,4-dihydroxytetrahydrofuran-2-yl)methyl ((*R*)-2-((3-fluoro-4-methoxybenzyl)oxy)-3-(octadecyloxy)propyl) hydrogen phosphate

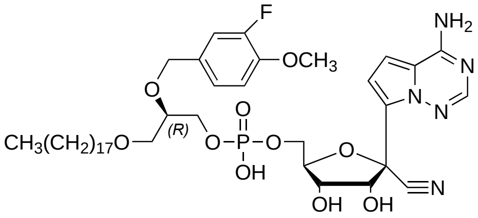

(Nanosyn)

1. ^{1}H NMR

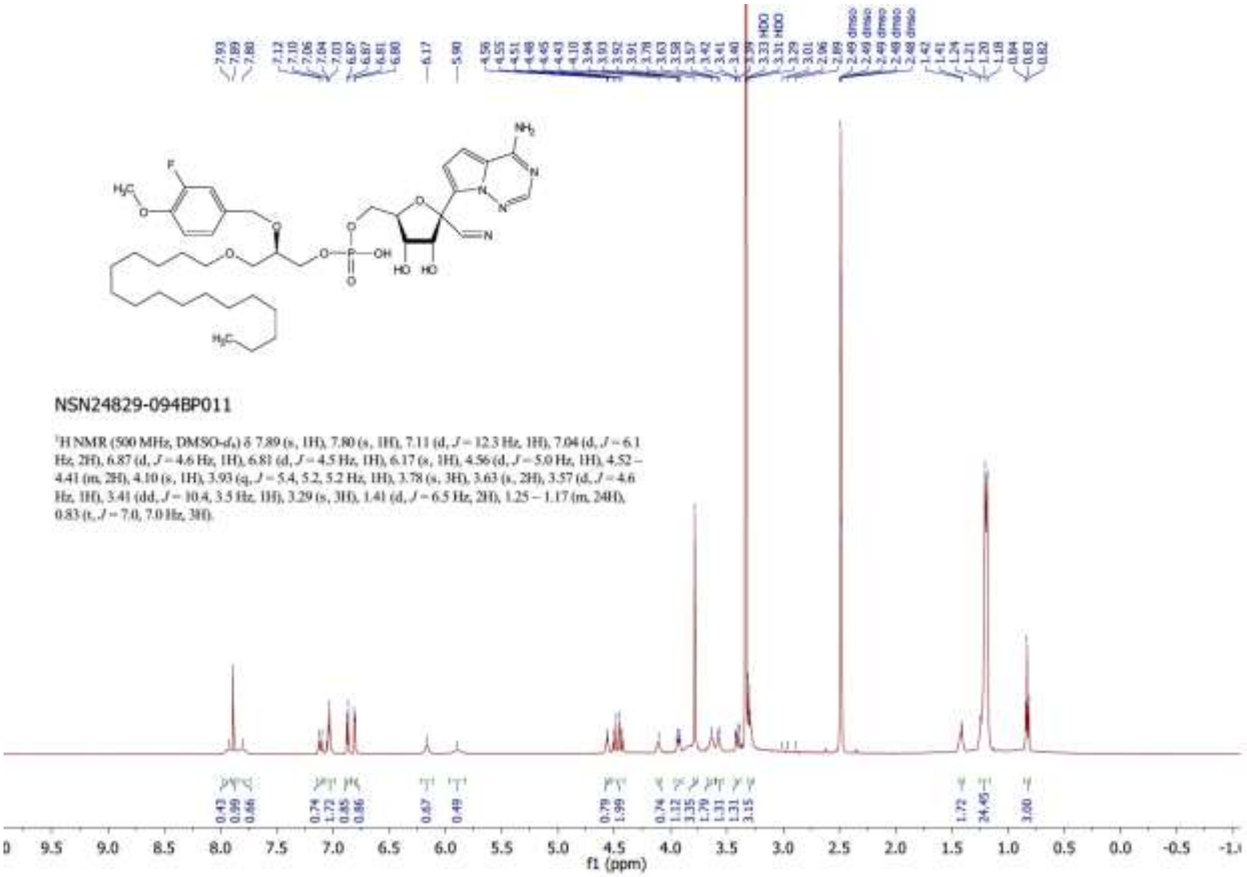

2. LCMS

ms filename	fmla Structure	Purity (%)	Weight (mg)	MW	NSID	Structure
093BP103	C41H63FN5O10P	94.7	15	835.43	NSN24773-093BP103_LCMS	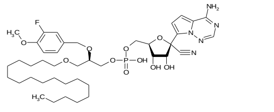

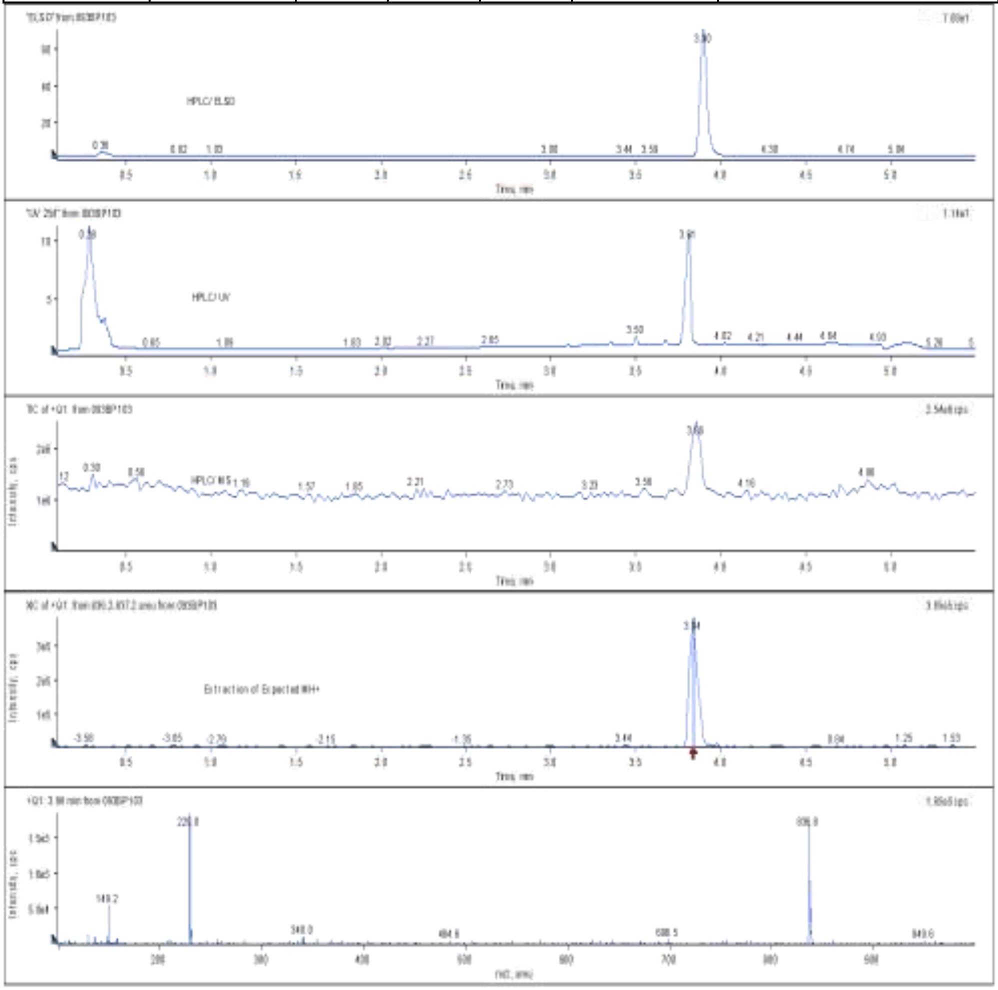

Compound 9h ((2*R*,3*S*,4*R*,5*R*)-5-(4-aminopyrrolo[2,1-*f*][1,2,4]triazin-7-yl)-5-cyano-3,4-dihydroxytetrahydrofuran-2-yl)methyl ((*R*)-3-(octadecyloxy)-2-(octyloxy)propyl) hydrogen phosphate

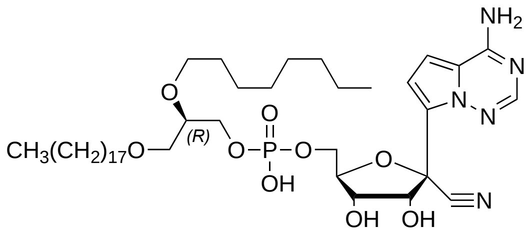

(Nanosyn)

1. ^{1}H NMR

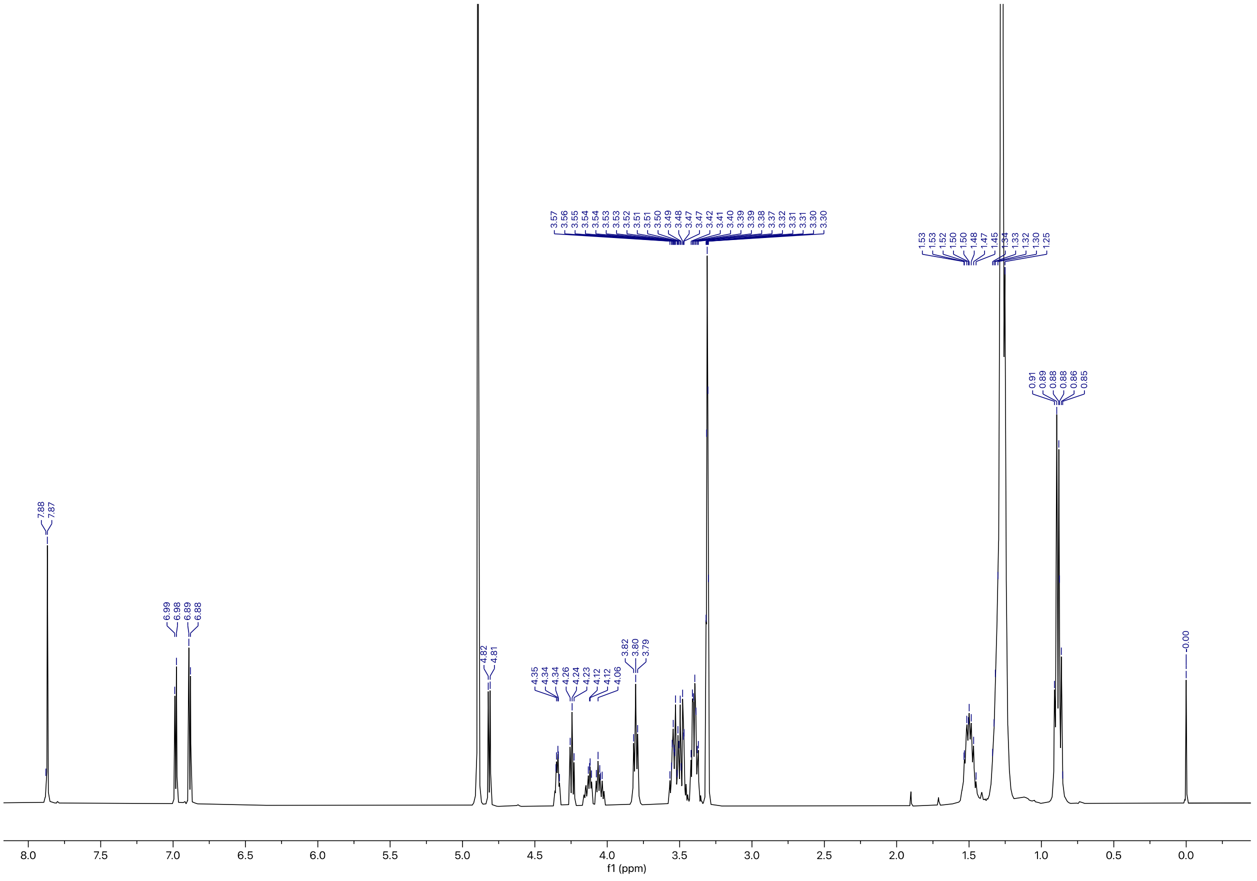

2. ^{13}C NMR

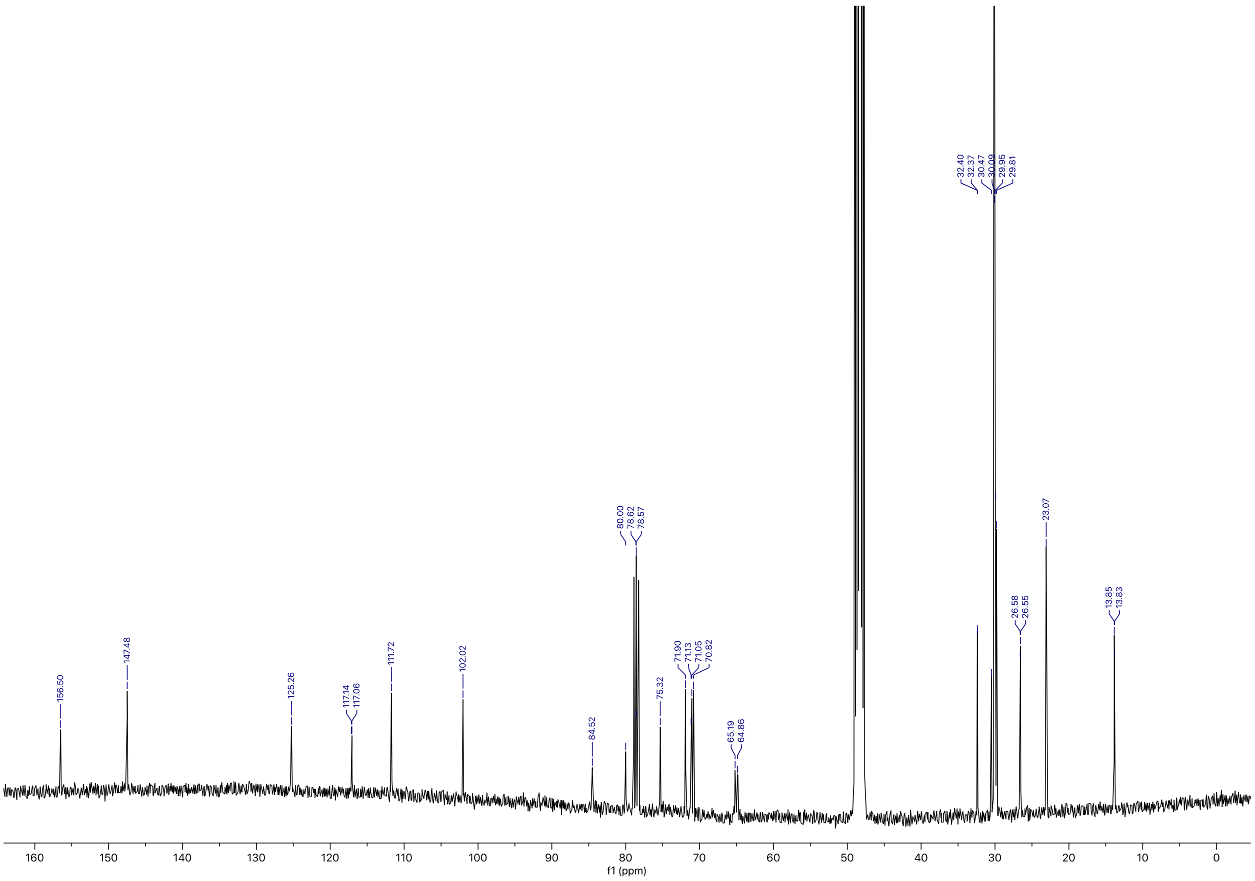

156.50
147.48
125.26
117.14
117.06
111.72
102.02
84.52
80.00
78.62
78.57
75.32
71.90
71.13
71.05
70.82
65.19
64.86
32.40
32.37
30.47
30.09
29.95
29.81
26.58
26.55
23.07
13.85
13.83
160
150
140
130
120
110
100
90
80
70
60
50
40
30
20
10
0
f1 (ppm)

3. HRMS

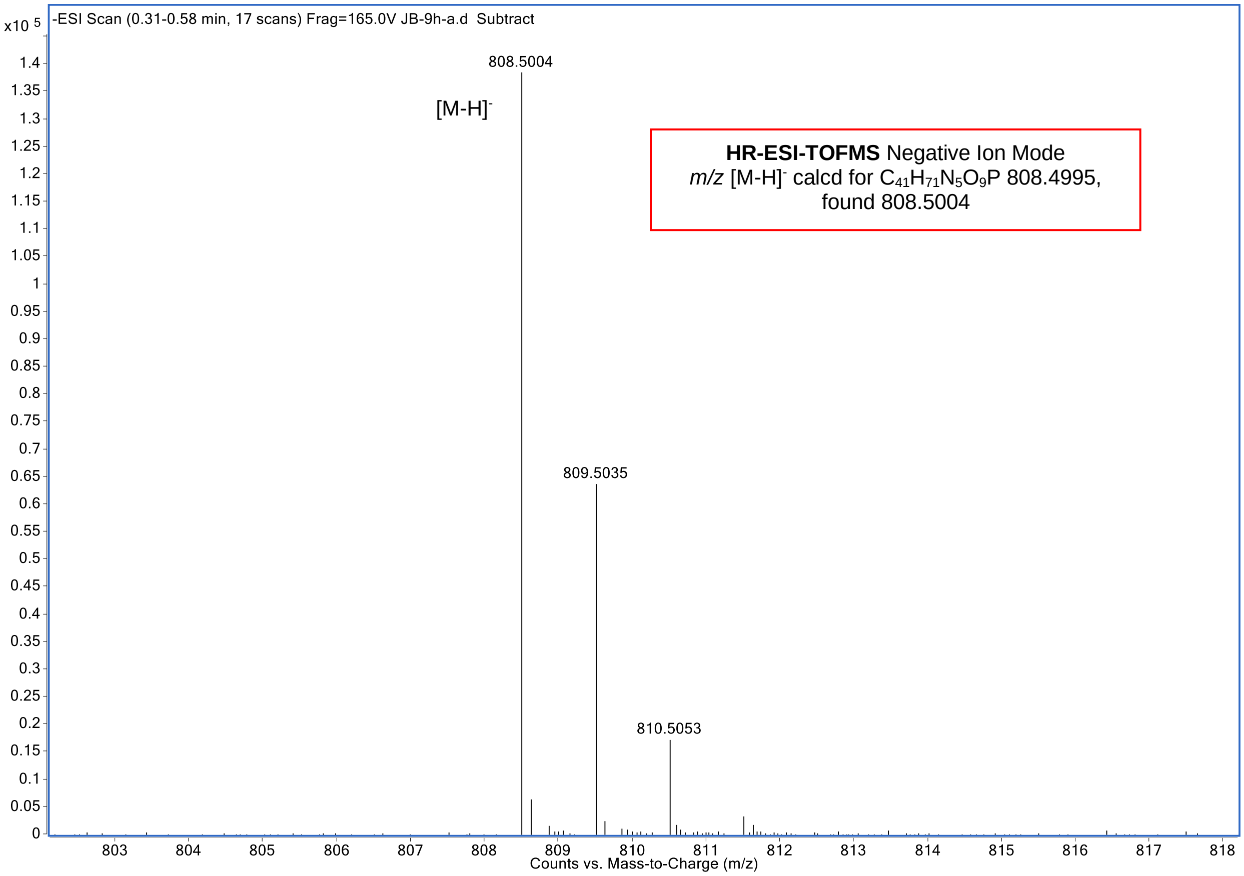
x10 5
-ESI Scan (0.31-0.58 min, 17 scans) Frag=165.0V JB-9h-a.d Subtract
808.5004
[M-H]-
HR-ESI-TOFMS Negative Ion Mode
m/z [M-H]- calcd for C41H71N5O9P 808.4995,
found 808.5004
809.5035
810.5053
Counts vs. Mass-to-Charge (m/z)

4. LCMS

ms filename	fmla Structure	Purity (%)	Weight (mg)	MW	NSID	Structure
094BP055	C41H72N5O9P	99.7	95.3	809.507	NSN24869-094BP055_LCMS	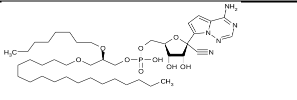

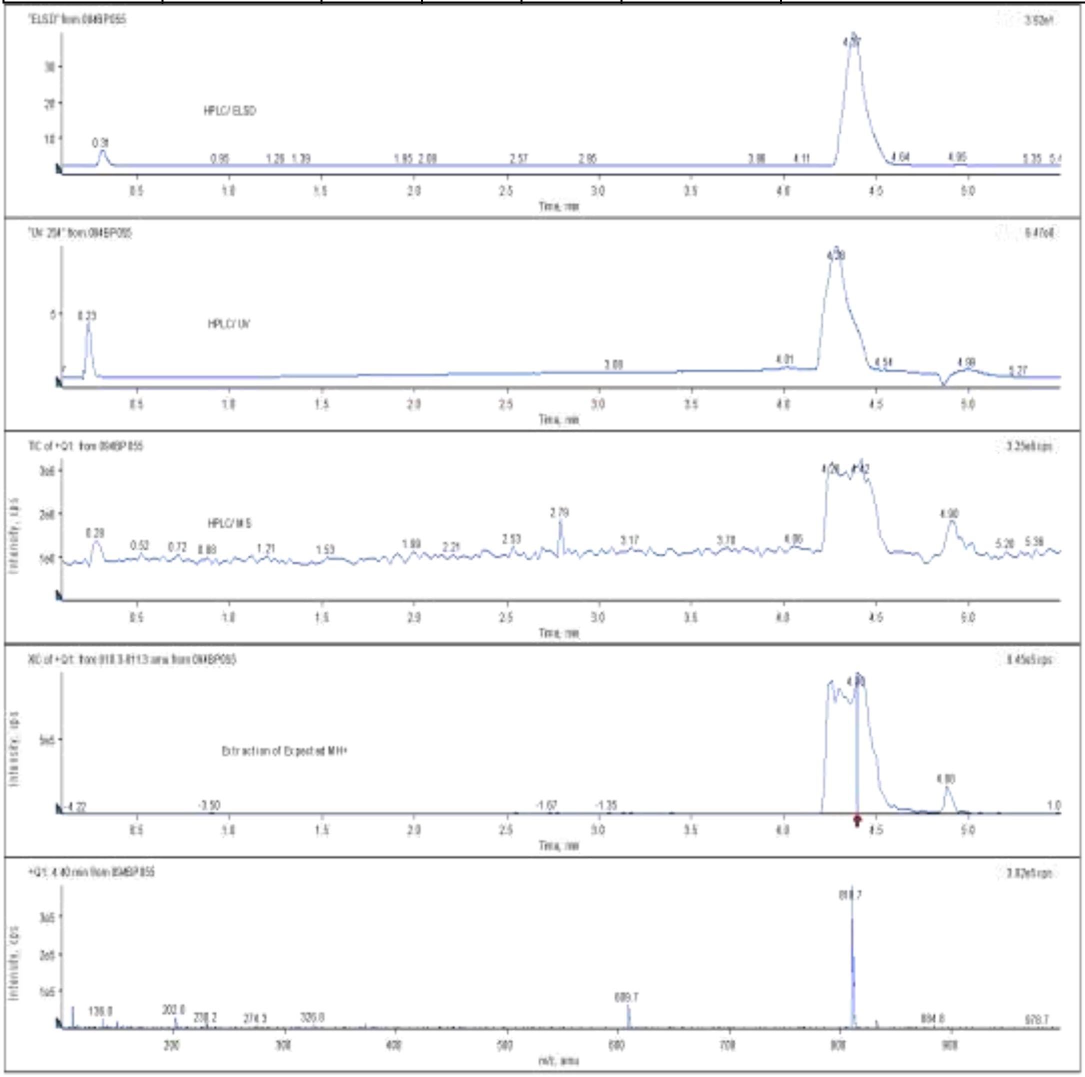

Compound 9i ((2*R*,3*S*,4*R*,5*R*)-5-(4-aminopyrrolo[2,1-*f*][1,2,4]triazin-7-yl)-5-cyano-3,4-dihydroxytetrahydrofuran-2-yl)methyl ((*R*)-2-(2-cyclohexylethoxy)-3-(octadecyloxy) propyl) hydrogen phosphate

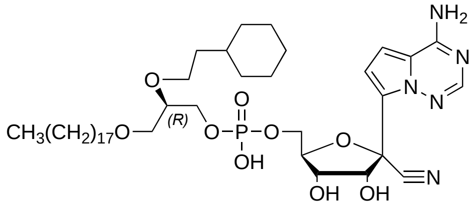

(Nanosyn)

1. ^{1}H NMR

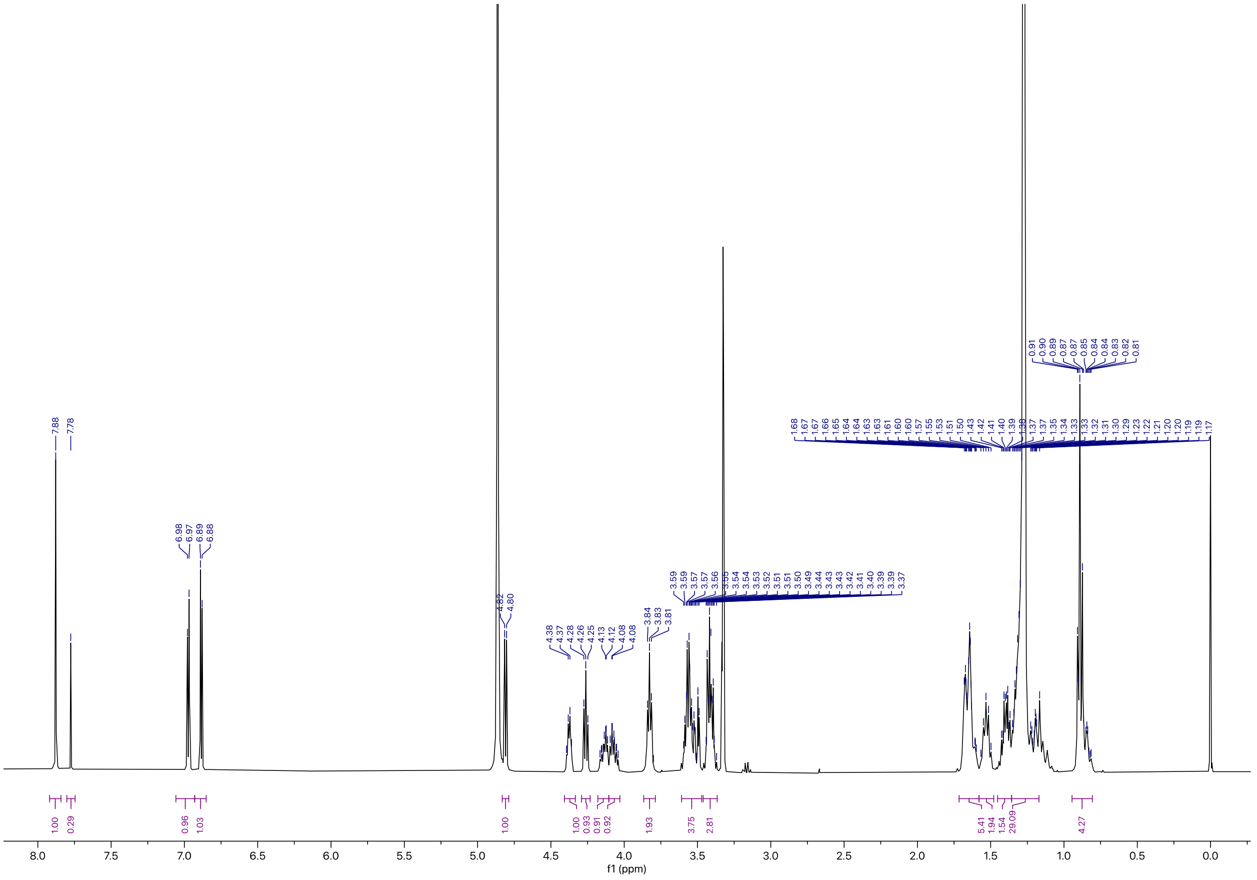

2. ^{13}C NMR

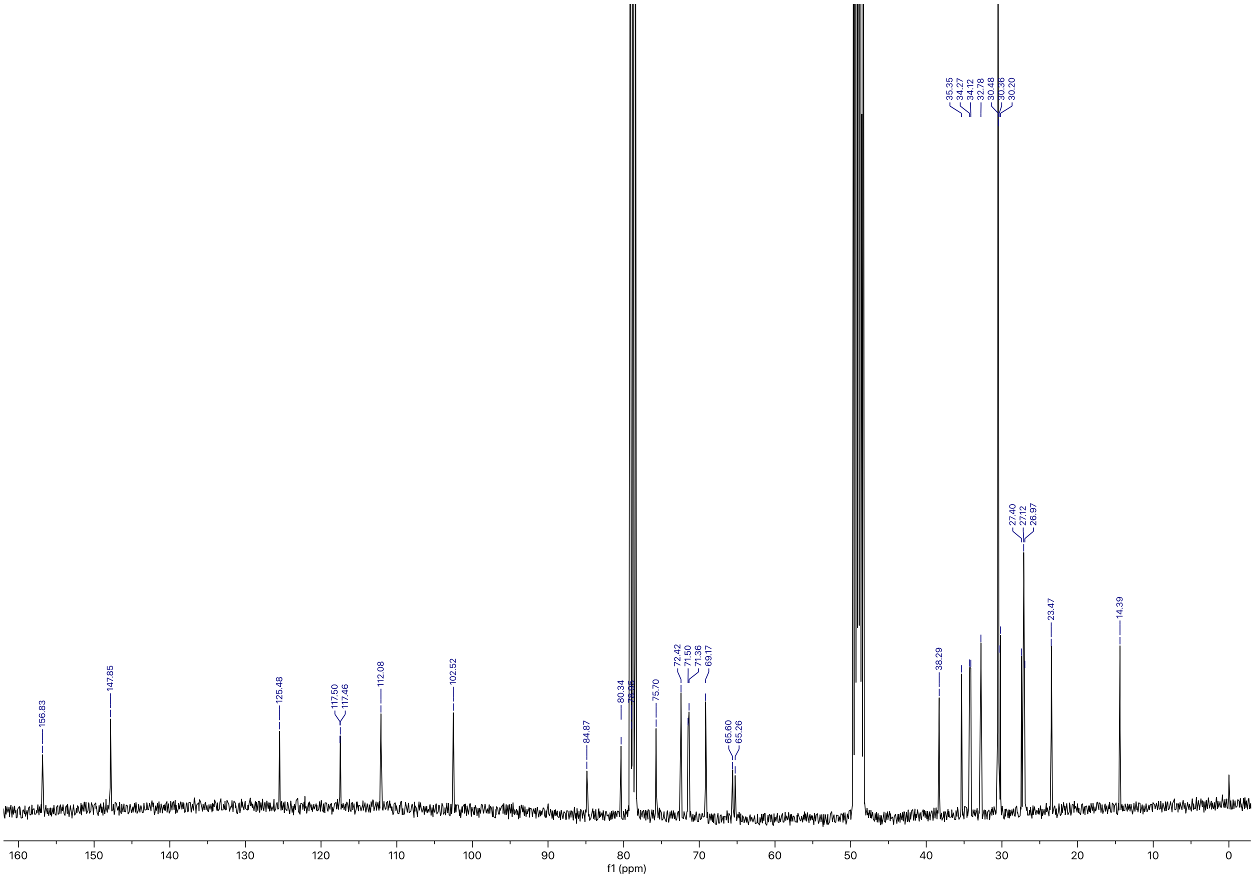
156.83
147.85
125.48
117.50
117.46
112.08
102.52
84.87
80.34
78.95
75.70
72.42
71.50
71.36
69.17
65.60
65.26
38.29
35.35
34.27
34.12
32.78
30.48
30.36
30.20
27.40
27.12
26.97
23.47
14.39
160 150 140 130 120 110 100 90 80 70 60 50 40 30 20 10 0
f1 (ppm)

3. HRMS

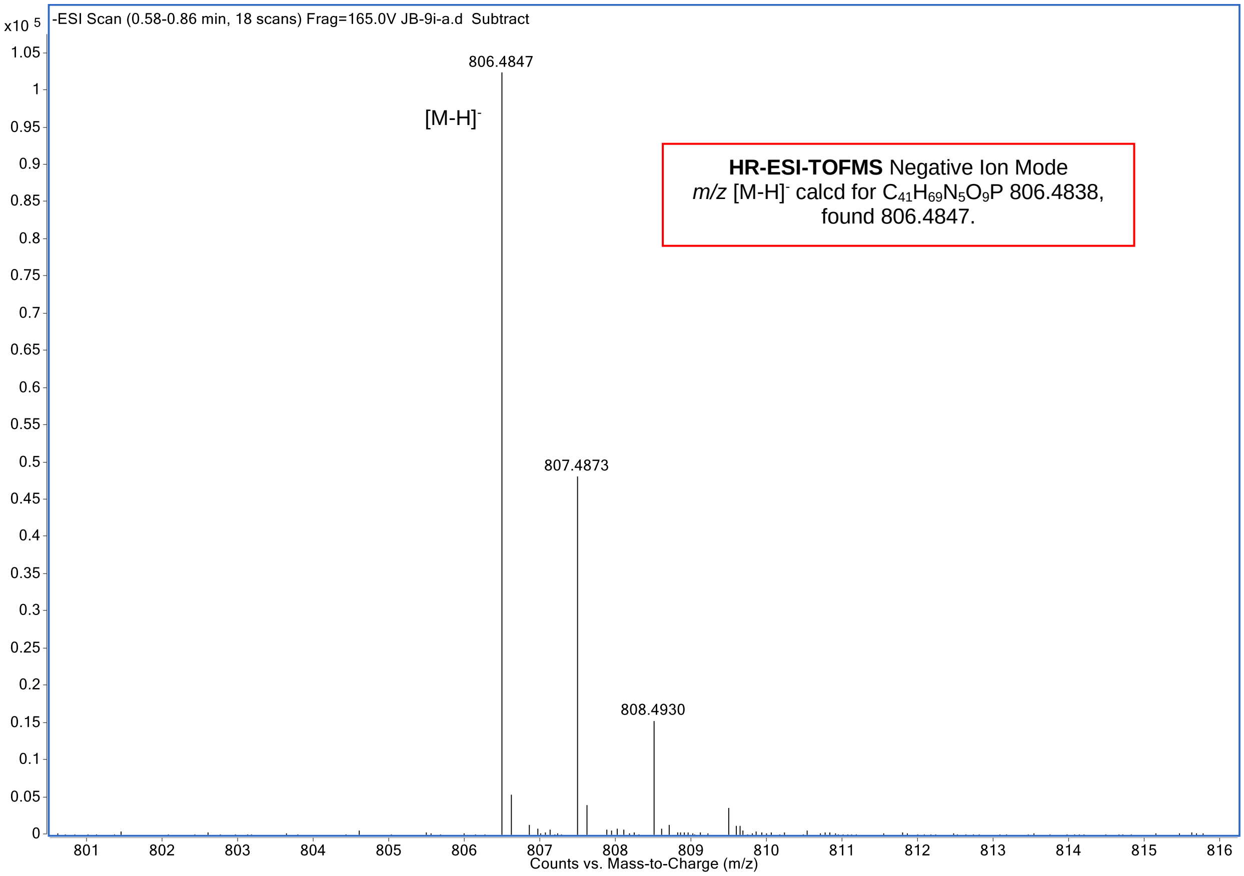

x10 5
-ESI Scan (0.58-0.86 min, 18 scans) Frag=165.0V JB-9i-a.d Subtract
806.4847
[M-H]-
807.4873
808.4930
HR-ESI-TOFMS Negative Ion Mode
m/z [M-H]- calcd for C41H69N5O9P 806.4838,
found 806.4847.
Counts vs. Mass-to-Charge (m/z)

4. LCMS

ms filename	fmla Structure	Purity (%)	Weight (mg)	MW	NSID	Structure
094BP065	C41H70N5O9P	99.8	246.2	807.491	NSN24883-094BP065_LCMS	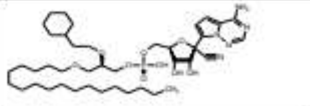

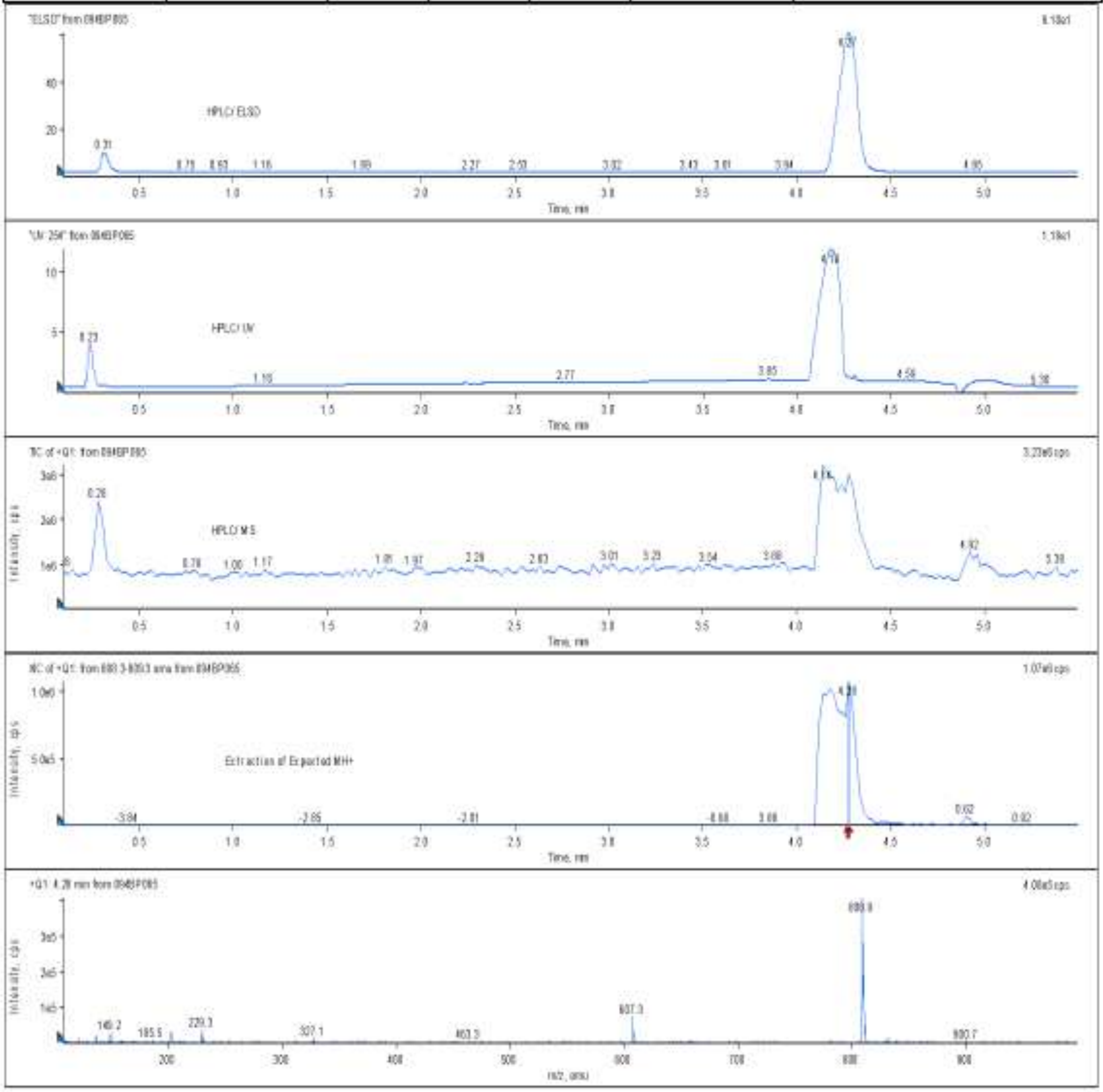

Compound 9j ((2*R*,3*S*,4*R*,5*R*)-5-(4-aminopyrrolo[2,1-*f*][1,2,4]triazin-7-yl)-5-cyano-3,4-dihydroxytetrahydrofuran-2-yl)methyl ((*R*)-2-((4-cyanobenzyl)oxy)-3-(octadecyloxy)propyl) hydrogen phosphate

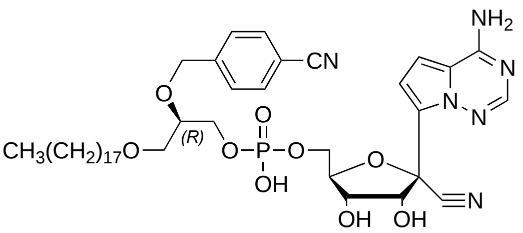

1. ^{1}H NMR

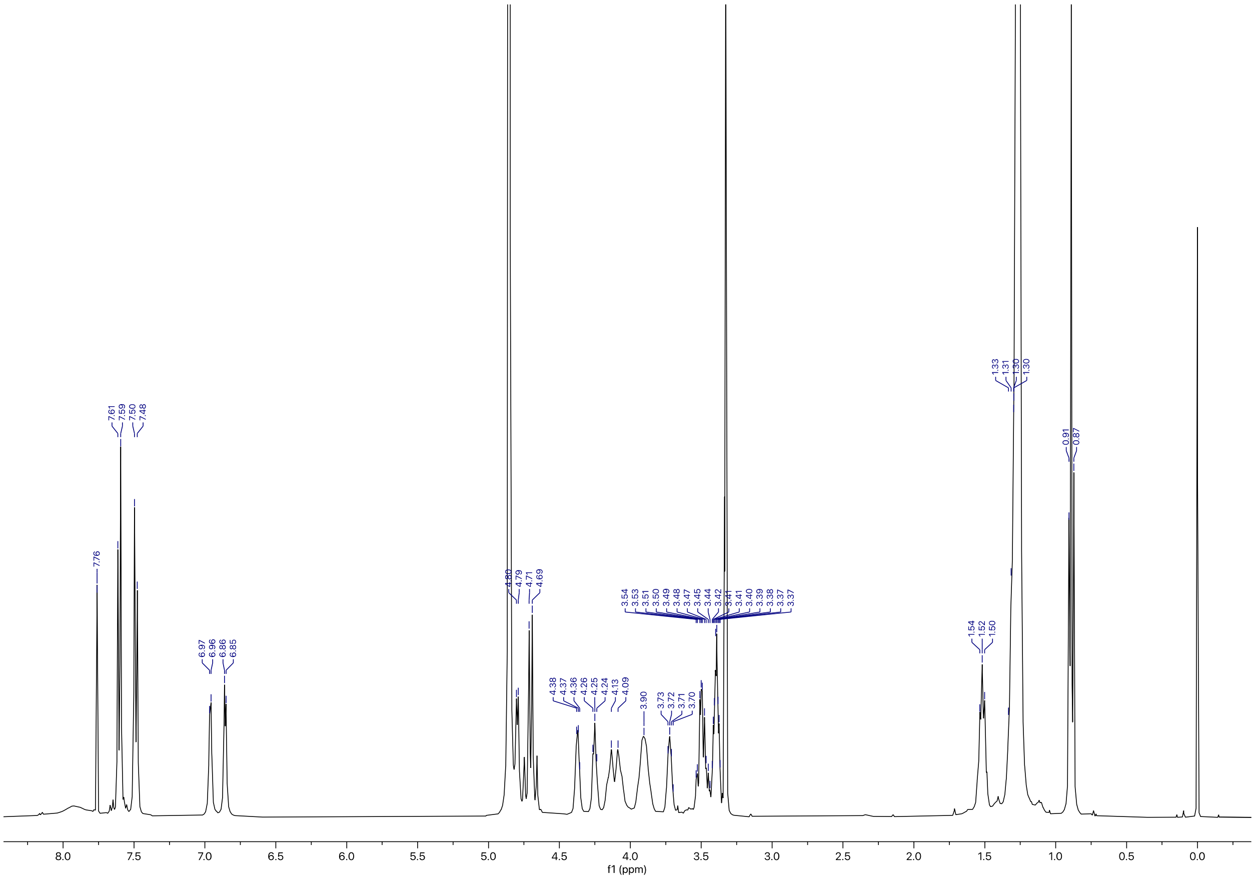

2. ^{13}C NMR

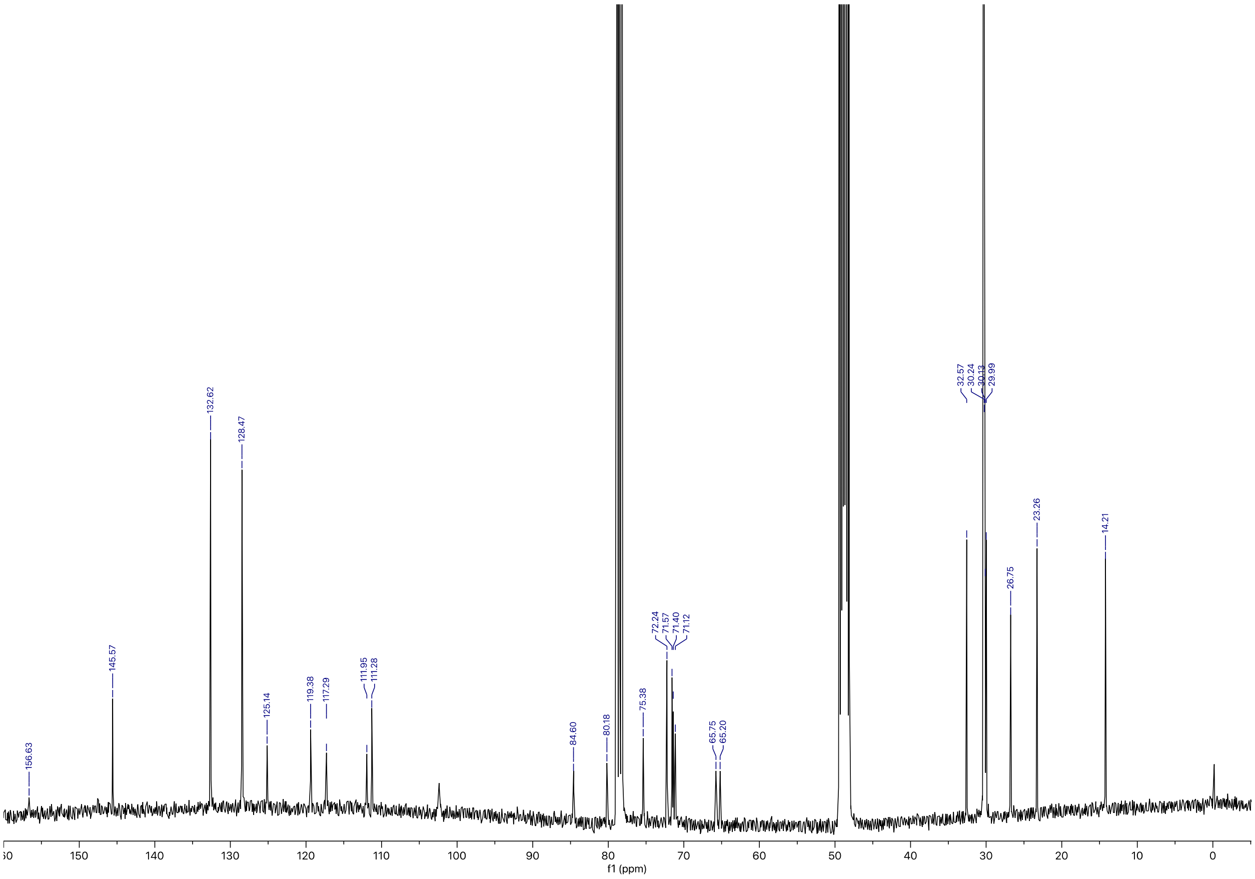
156.63
145.57
132.62
128.47
125.14
119.38
117.29
111.95
111.28
84.60
80.18
75.38
72.24
71.57
71.40
71.12
65.75
65.20
32.57
30.24
30.13
29.99
26.75
23.26
14.21
150
140
130
120
110
100
90
80
70
60
50
40
30
20
10
0
f1 (ppm)

3. HRMS

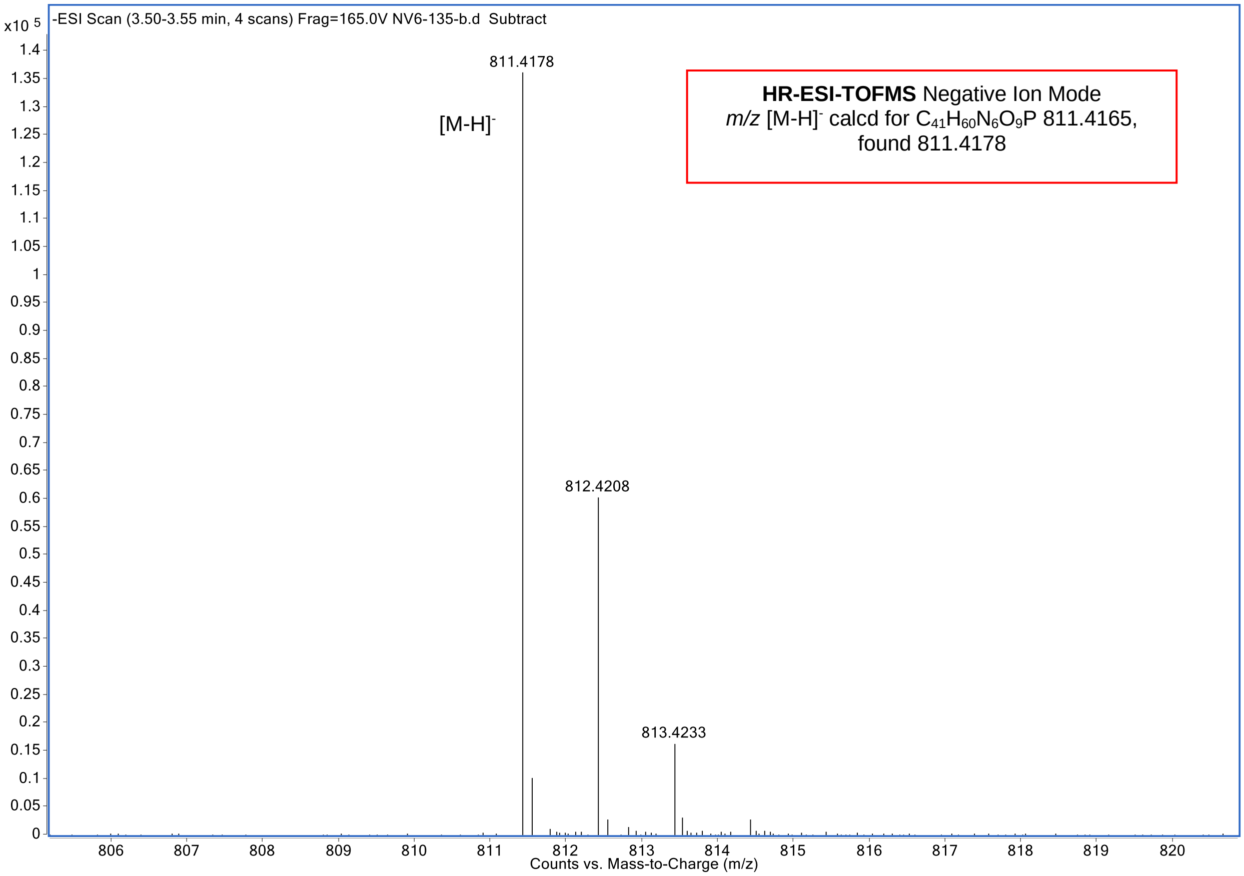

-ESI Scan (3.50-3.55 min, 4 scans) Frag=165.0V NV6-135-b.d Subtract
x10 5
811.4178
[M-H]-
812.4208
813.4233
HR-ESI-TOFMS Negative Ion Mode
m/z [M-H]- calcd for C41H60N6O9P 811.4165,
found 811.4178
Counts vs. Mass-to-Charge (m/z)

4. HPLC

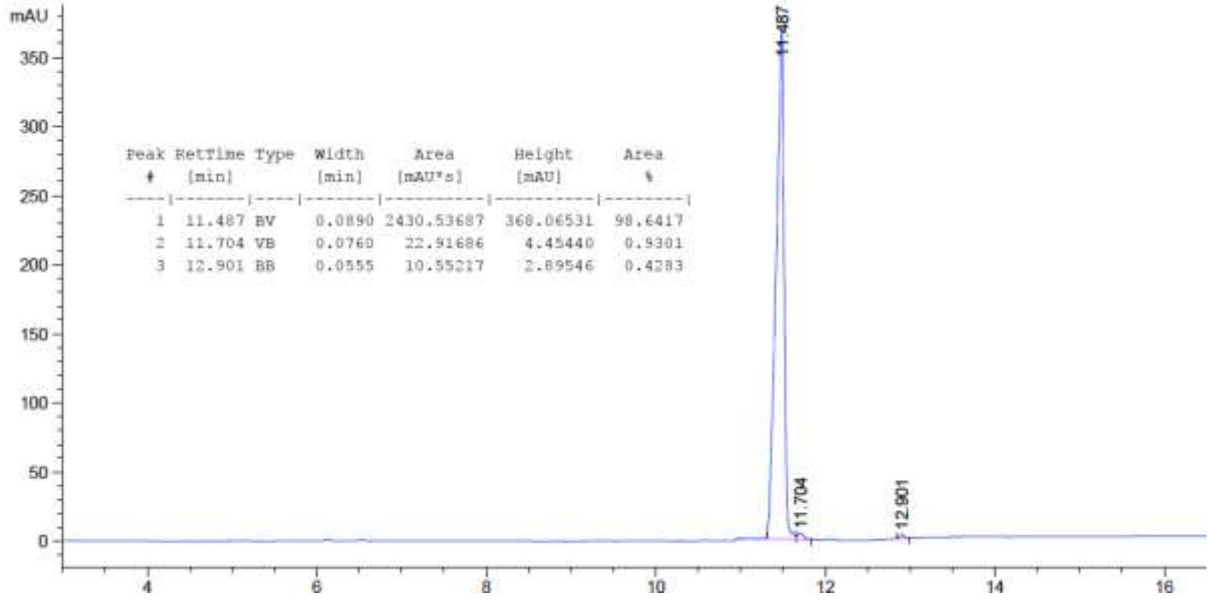

Peak #	RetTime [min]	Type	Width [min]	Area [mAU*s]	Height [mAU]	Area %
1	11.487	BV	0.0890	2430.53687	368.06531	98.6417
2	11.704	VB	0.0760	22.91686	4.45440	0.9301
3	12.901	BB	0.0555	10.55217	2.89546	0.4283

Compound 9k ((2R,3S,4R,5R)-5-(4-aminopyrrolo[2,1-f][1,2,4]triazin-7-yl)-5-cyano-3,4-dihydroxytetrahydrofuran-2-yl)methyl ((R)-2-((3-cyanobenzyl)oxy)-3-(octadecyloxy)propyl) hydrogen phosphate

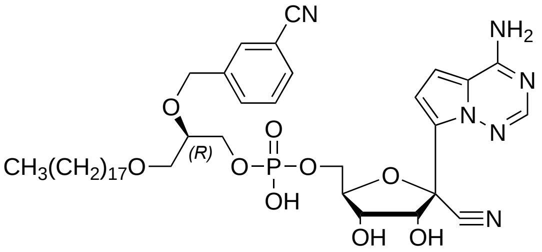

1. ^{1}H NMR

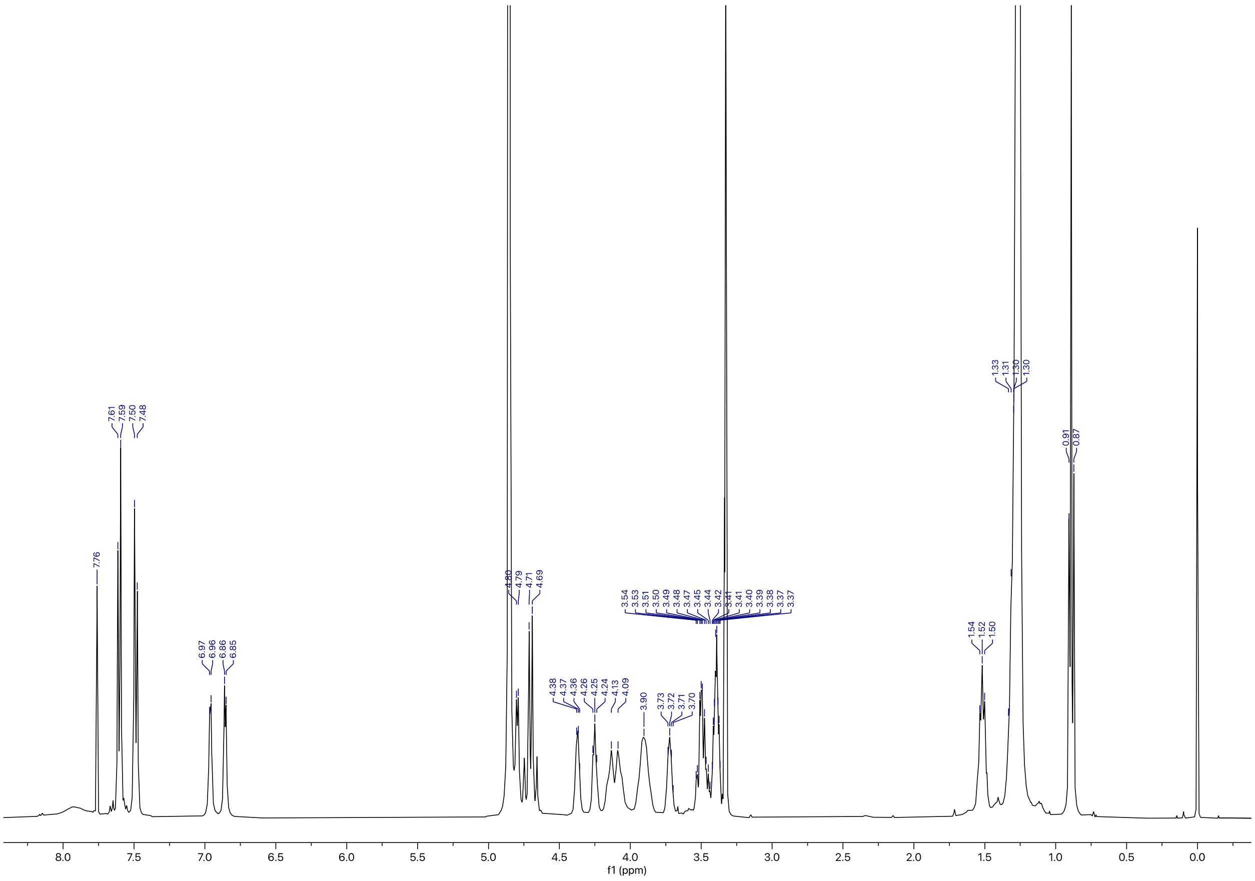

2. ^{13}C NMR

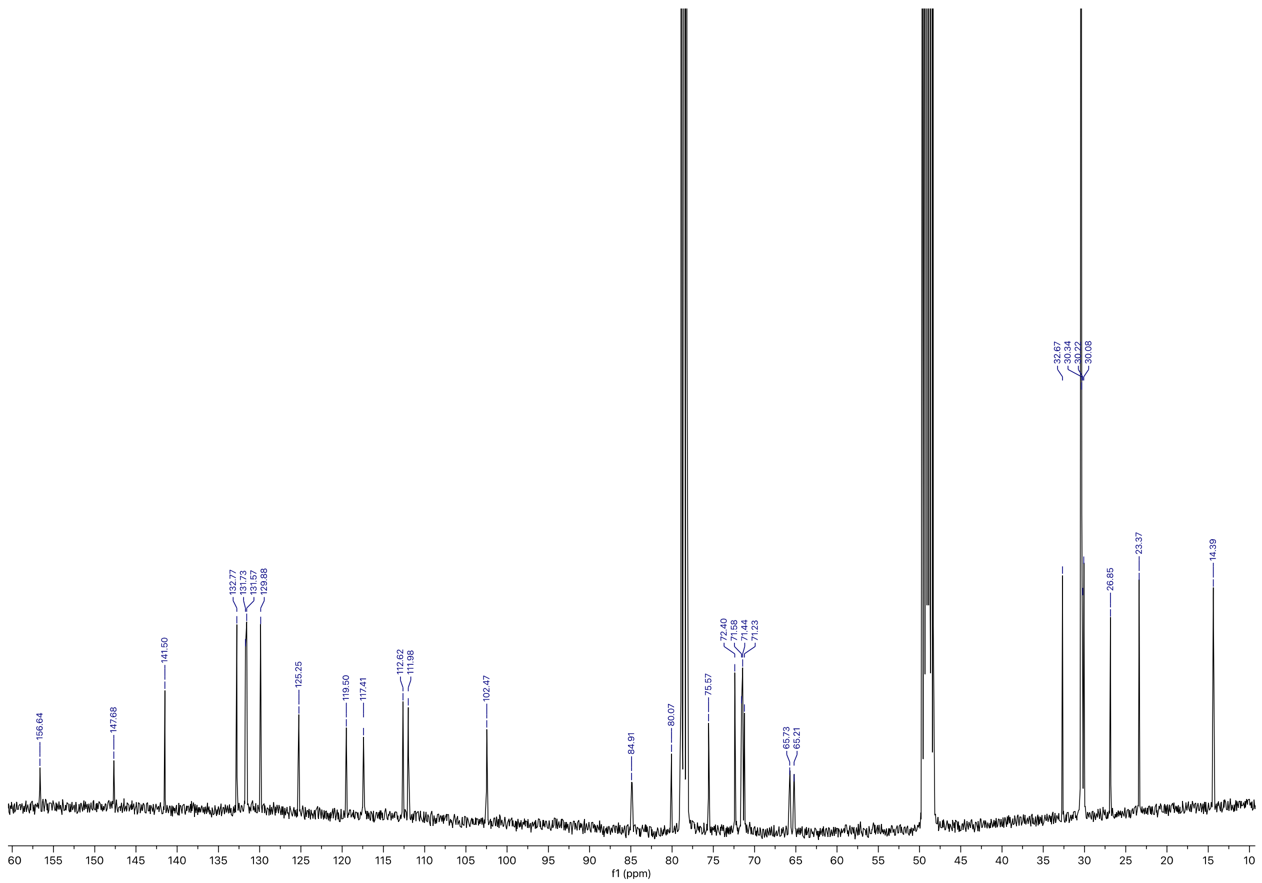

156.64
147.68
141.50
132.77
131.73
131.57
129.88
125.25
119.50
117.41
112.62
111.98
102.47
84.91
80.07
75.57
72.40
71.58
71.44
71.23
65.73
65.21
32.67
30.34
30.22
30.08
26.85
23.37
14.39
f1 (ppm)

3. HRMS

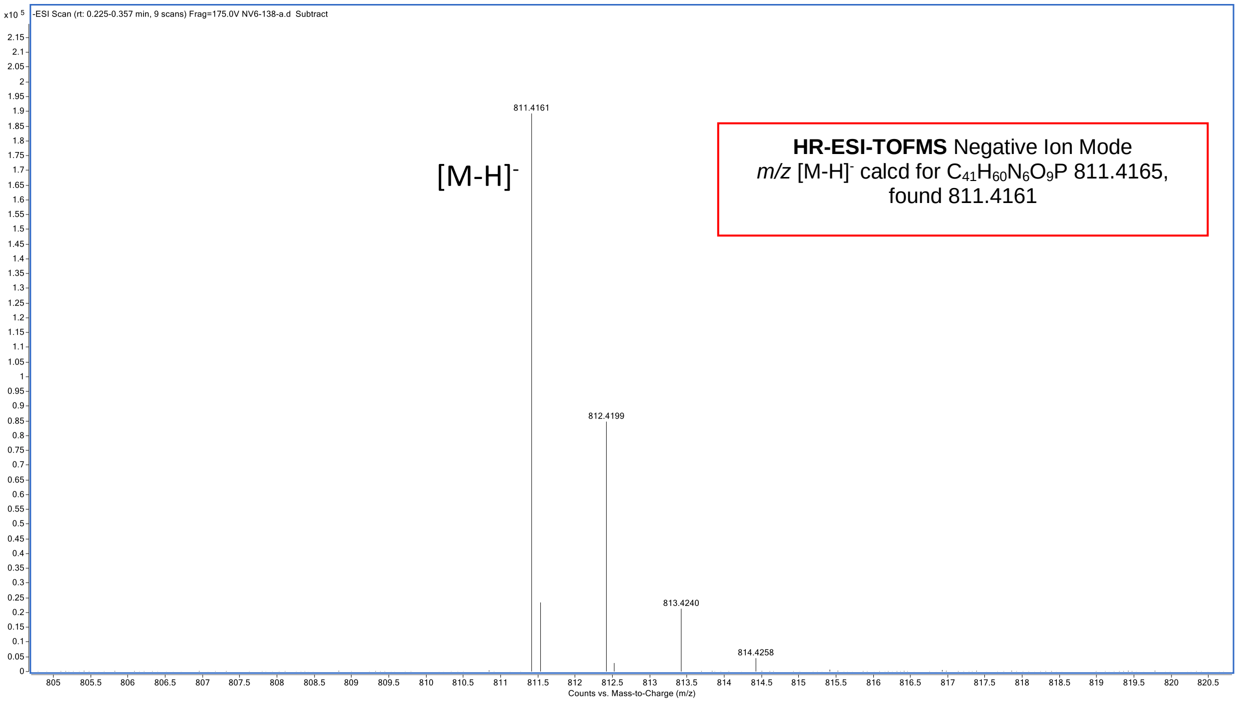

4. HPLC

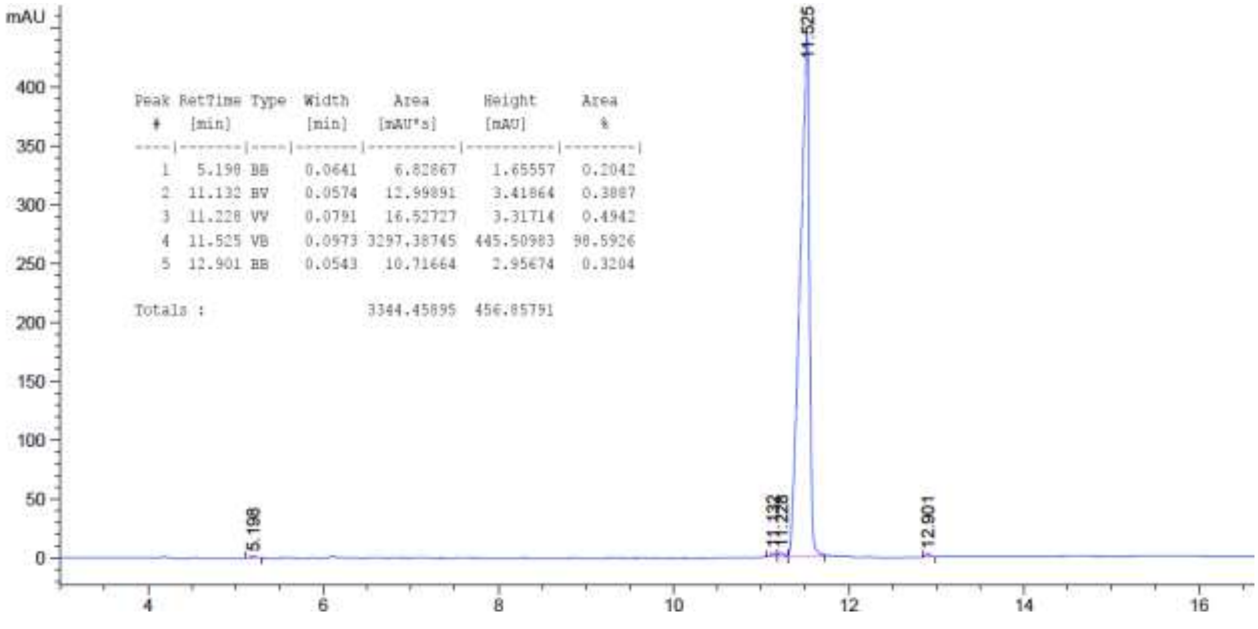

Peak #	RetTime [min]	Type	Width [min]	Area [mAU*s]	Height [mAU]	Area %
1	5.198	BB	0.0641	6.82867	1.65557	0.2042
2	11.132	BV	0.0574	12.99891	3.41864	0.3887
3	11.228	VV	0.0791	16.52727	3.31714	0.4942
4	11.525	VB	0.0973	3297.38745	445.50983	98.5926
5	12.901	BB	0.0543	10.71664	2.95674	0.3204
Totals :				3344.45895	456.85791	

Compound 9l ((2R,3S,4R,5R)-5-(4-aminopyrrolo[2,1-f][1,2,4]triazin-7-yl)-5-cyano-3,4-dihydroxytetrahydrofuran-2-yl)methyl ((R)-2-((3-cyanobenzyl)oxy)-3-(octadecyloxy)propyl) hydrogen phosphate

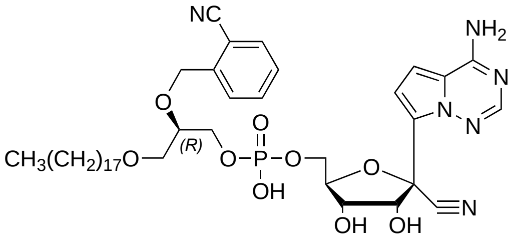

^{1}H NMR

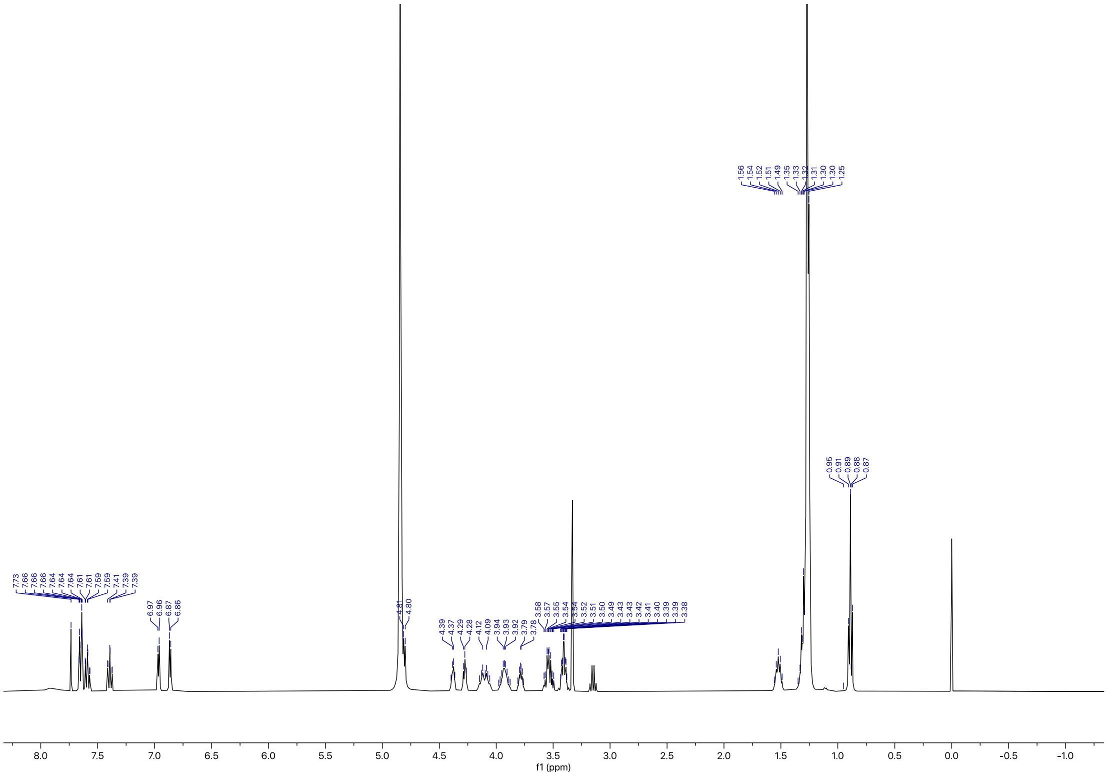

^{13}C NMR

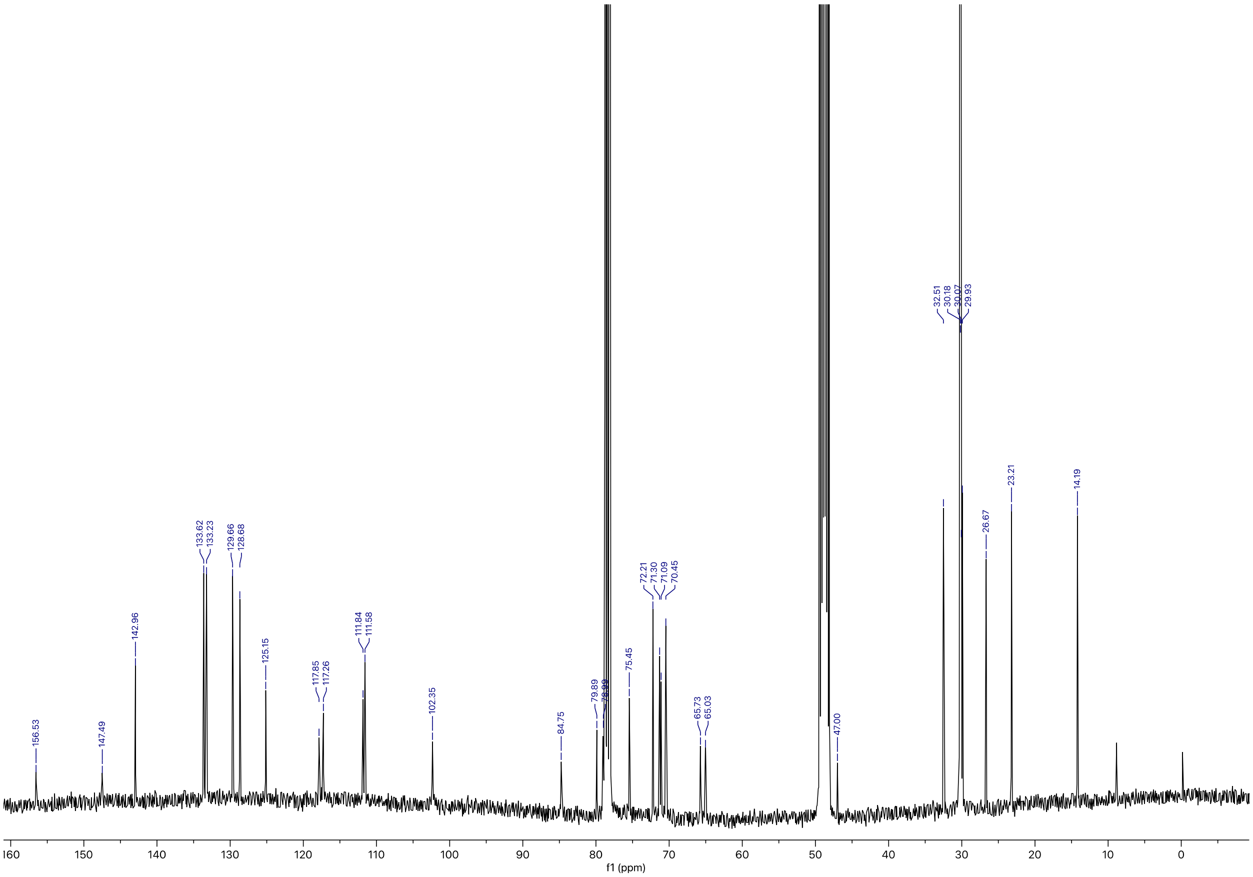
156.53
147.49
142.96
133.62
133.23
129.66
128.68
125.15
117.85
117.26
111.84
111.58
102.35
84.75
79.89
75.45
72.21
71.30
71.09
70.45
65.73
65.03
47.00
32.51
30.18
30.07
29.93
26.67
23.21
14.19
f1 (ppm)

HRMS

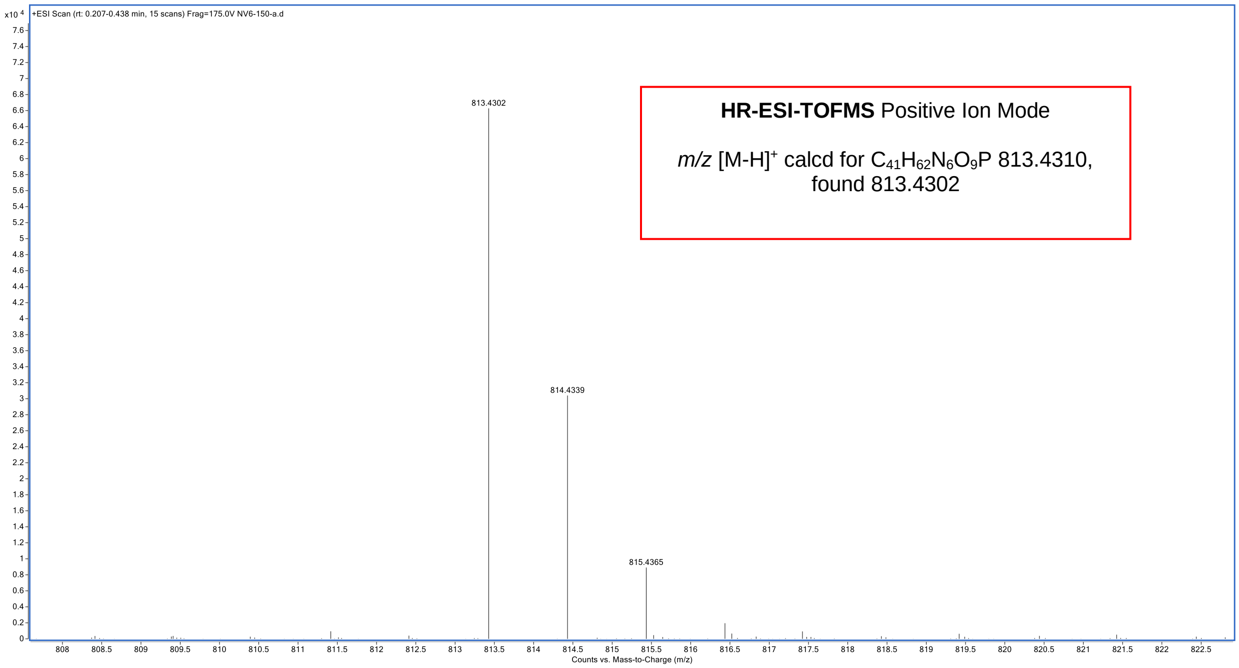

HPLC

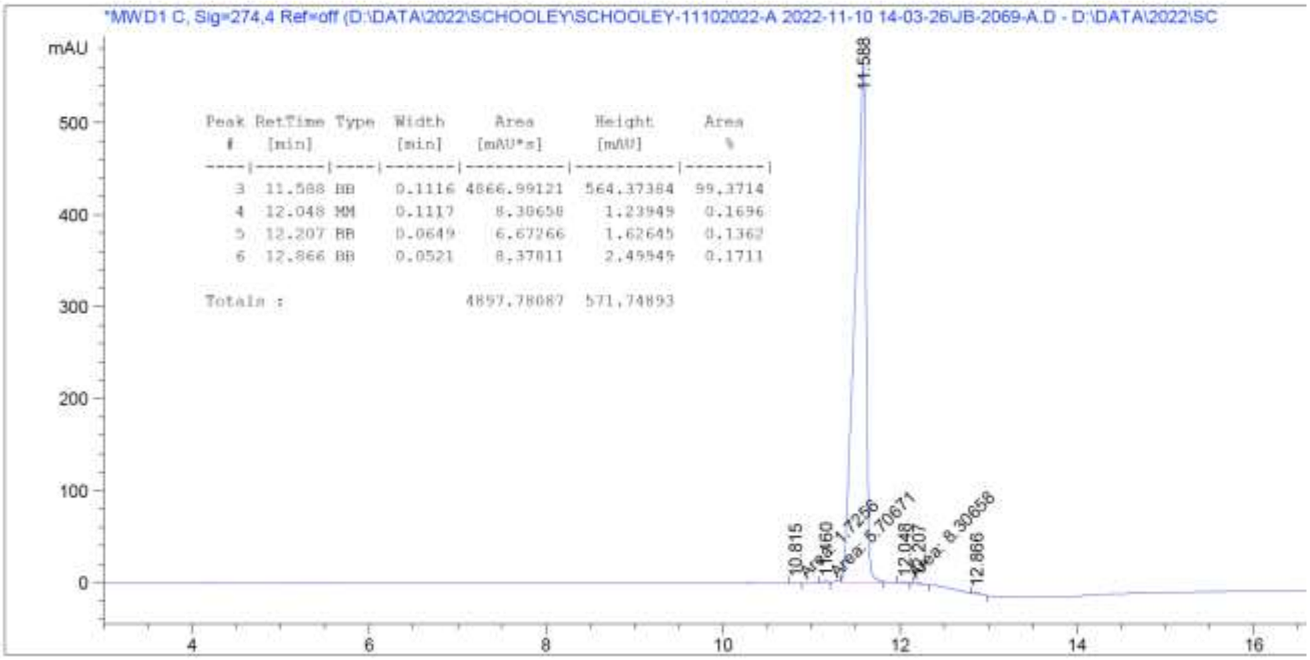

Peak #	RetTime [min]	Type	Width [min]	Area [mAU*s]	Height [mAU]	Area %
3	11.588	BB	0.1116	4866.99121	564.37384	99.3714
4	12.048	MM	0.1117	8.30658	1.23949	0.1696
5	12.207	BB	0.0649	6.67266	1.62645	0.1362
6	12.866	BB	0.0521	8.37811	2.45949	0.1711
Totals :				4897.78087	571.74893	

Compound 10a ((2*R*,3*S*,4*R*,5*R*)-5-(4-aminopyrrolo[2,1-*f*][1,2,4]triazin-7-yl)-5-cyano-3,4-dihydroxytetrahydrofuran-2-yl)methyl ((*R*)-2-(benzyloxy)-7-(tetradec-5-en-1-yloxy)heptyl) hydrogen phosphate

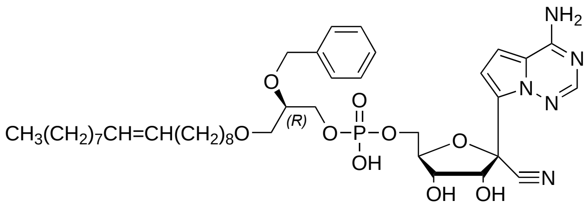

(J-Star Research)

1. ^{1}H NMR

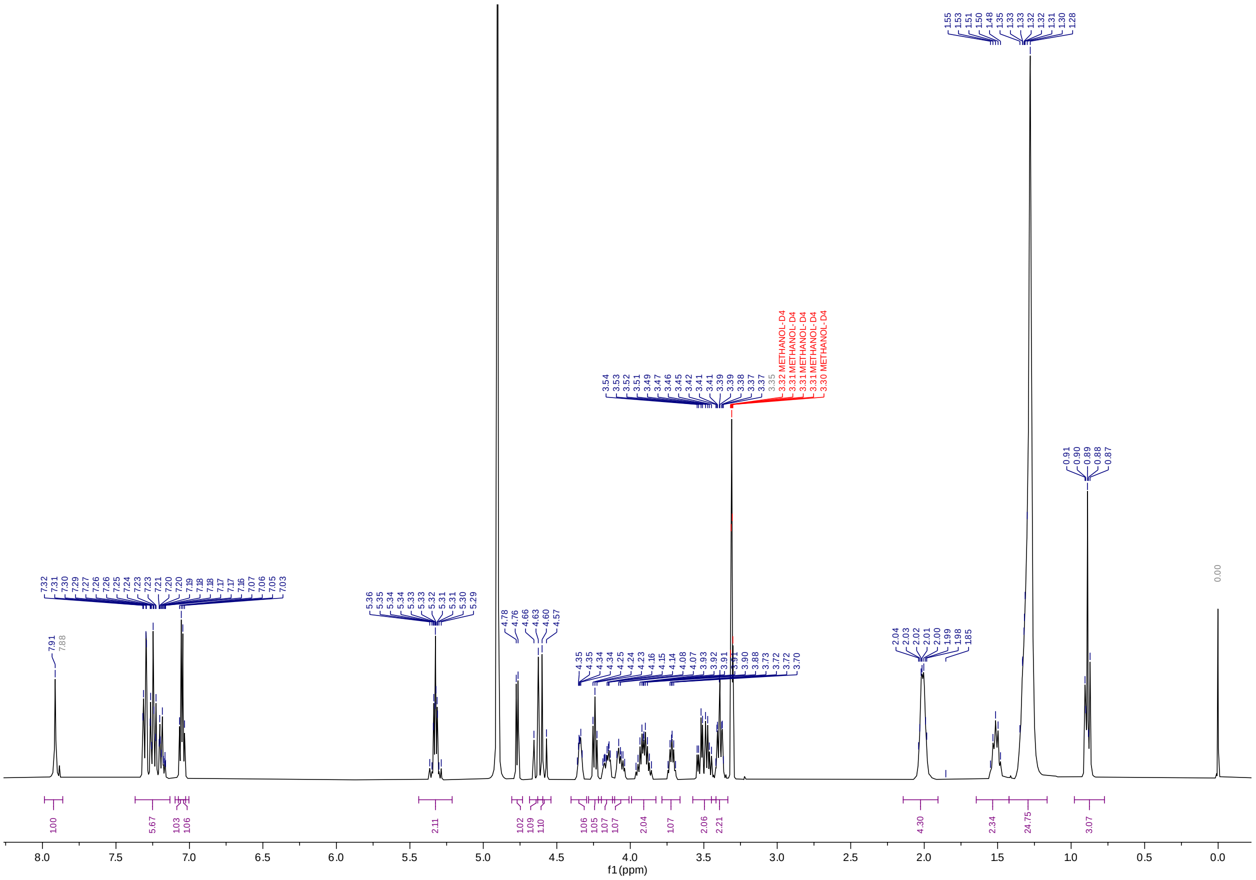

2. ^{13}C NMR

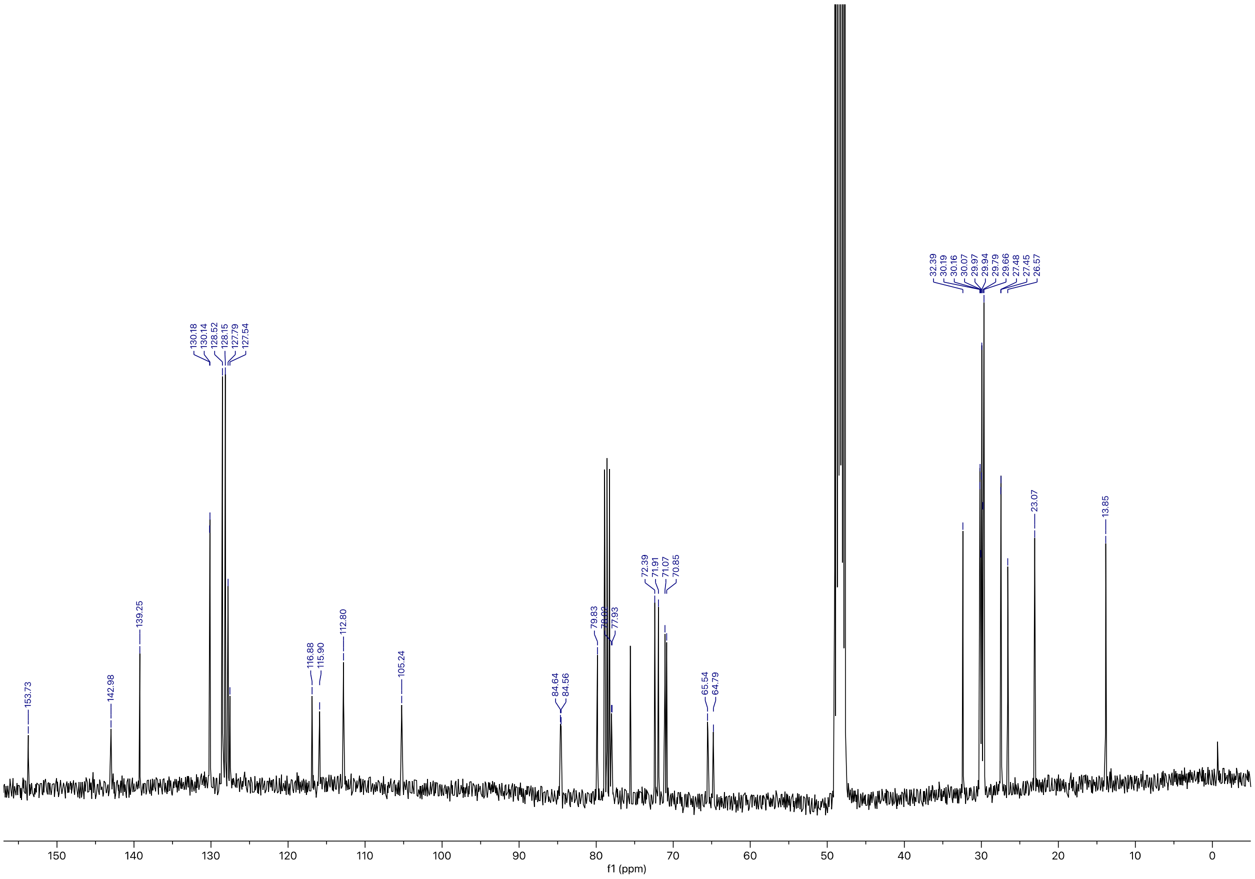

3. ^{31}P NMR

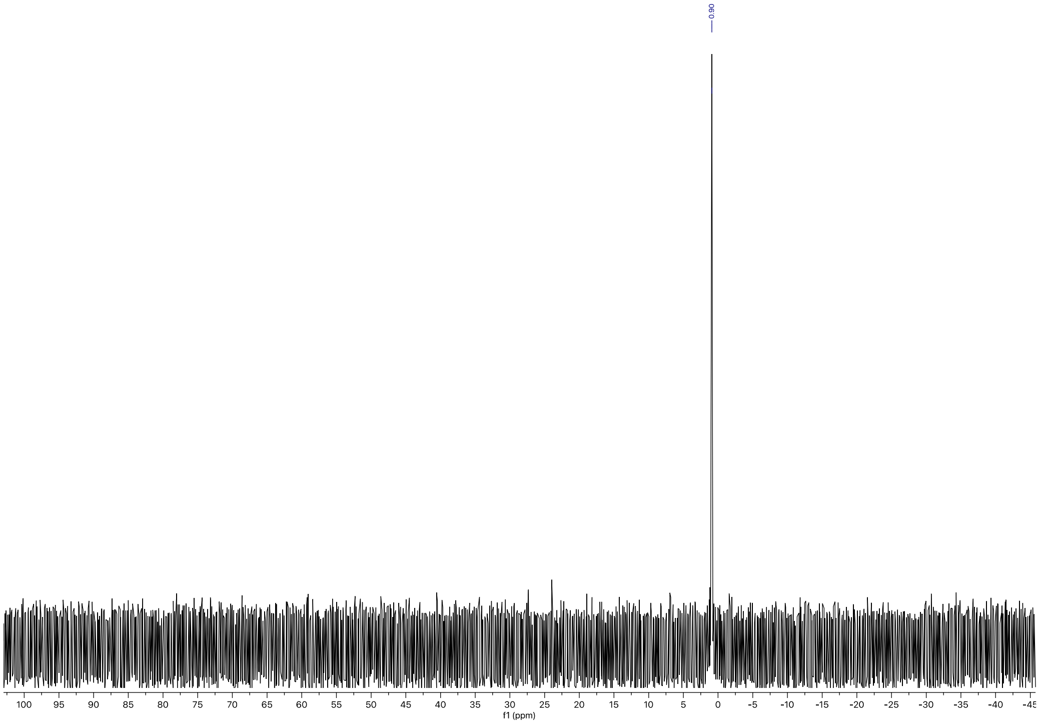

4. HRMS

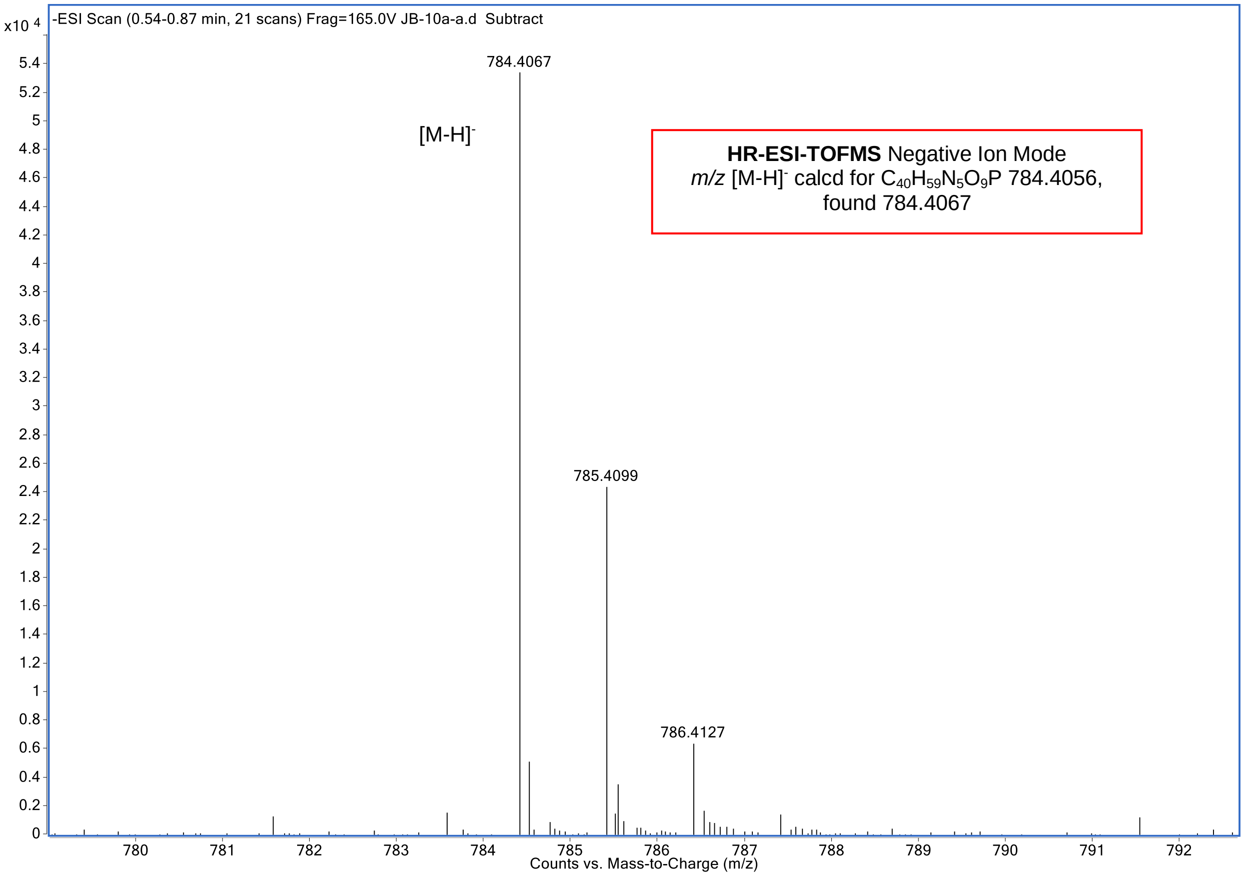

x10 4
-ESI Scan (0.54-0.87 min, 21 scans) Frag=165.0V JB-10a-a.d Subtract
784.4067
[M-H]-
785.4099
786.4127
HR-ESI-TOFMS Negative Ion Mode
m/z [M-H]- calcd for C40H59N5O9P 784.4056,
found 784.4067
Counts vs. Mass-to-Charge (m/z)

5. HPLC

Certificate of Analysis

Name of Compound: Phosphate A

Lot #: 861-77-28

Amount Shipped (Date): 0.302 g (06/17/2021)

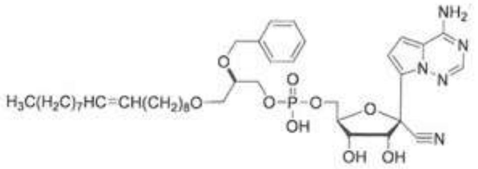

Physical Properties:

Molecular Weight:	785.92
Molecular Formula:	$C_{40}H_{60}N_5O_9P$
Color, Form and Appearance:	off white solid
HPLC Purity:	> 99 area %
LCMS (ESI; $[M+H]^+$))	786.78
300 MHz ^1H NMR ($CDCl_3$/MeOH-d4):	conforms

John Reichwein, Ph.D.
Associate Director Process Chemistry
Date: 06/17/2021

Compound 10g ((2*R*,3*S*,4*R*,5*R*)-5-(4-aminopyrrolo[2,1-*f*][1,2,4]triazin-7-yl)-5-cyano-3,4-dihydroxytetrahydrofuran-2-yl)methyl ((*R*)-2-((3-fluoro-4-methoxybenzyl)oxy)-7-(tetradec-5-en-1-yloxy)heptyl) hydrogen phosphate

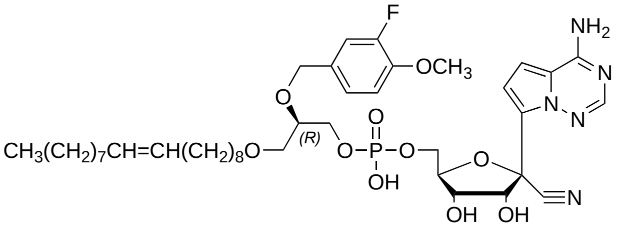

(J-Star Research)

1. ^{1}H NMR

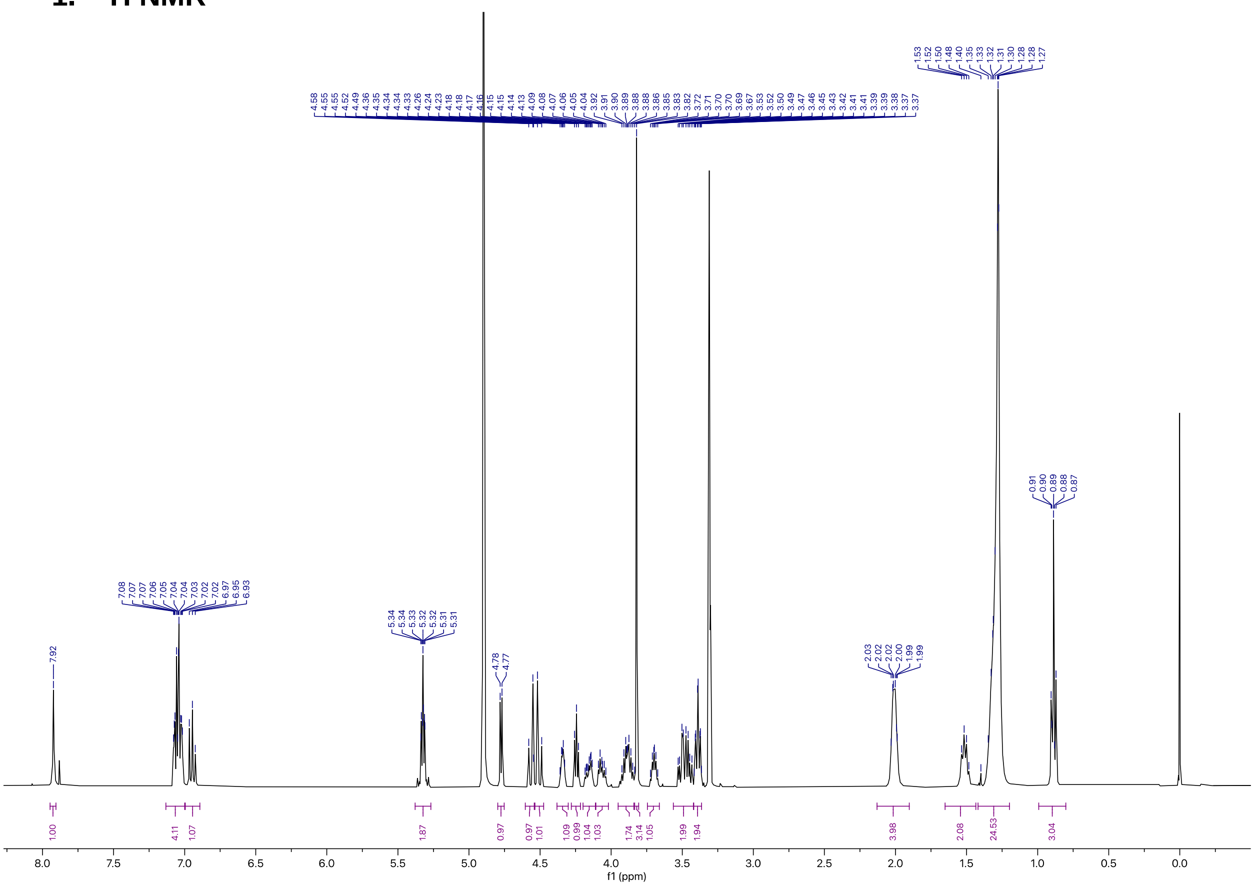

2. ^{13}C NMR

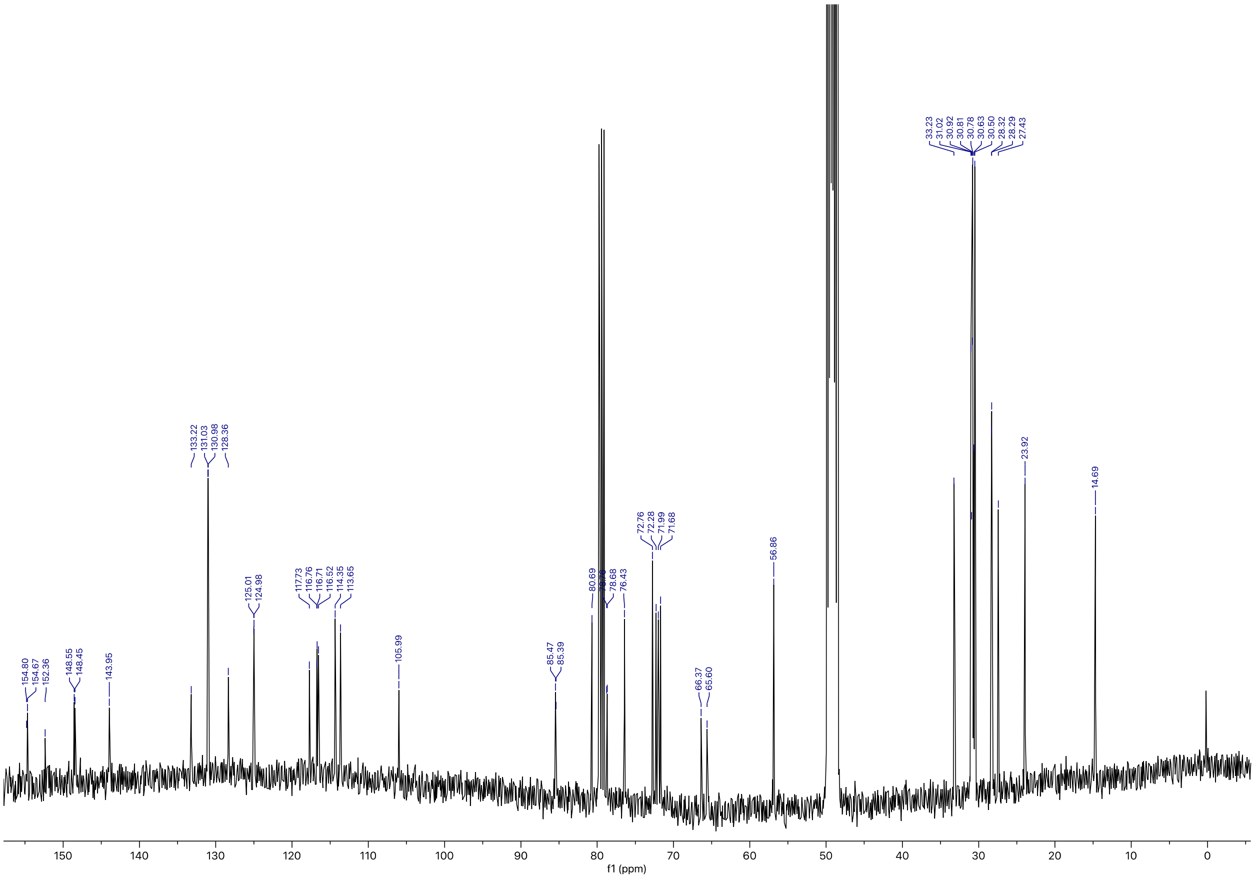

3. ^{31}P NMR

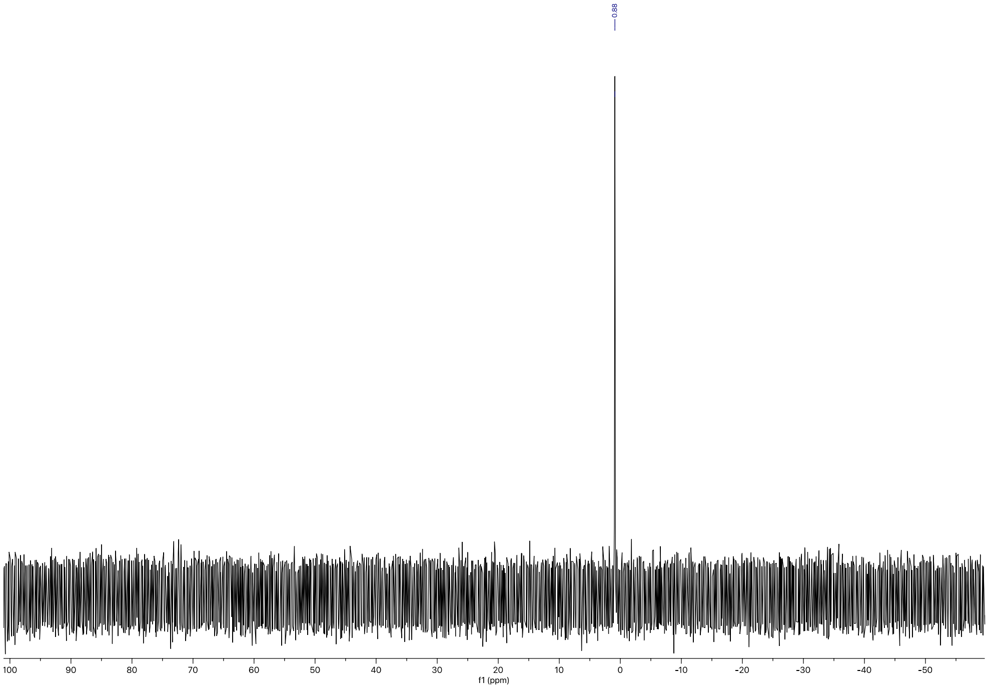

4. HRMS

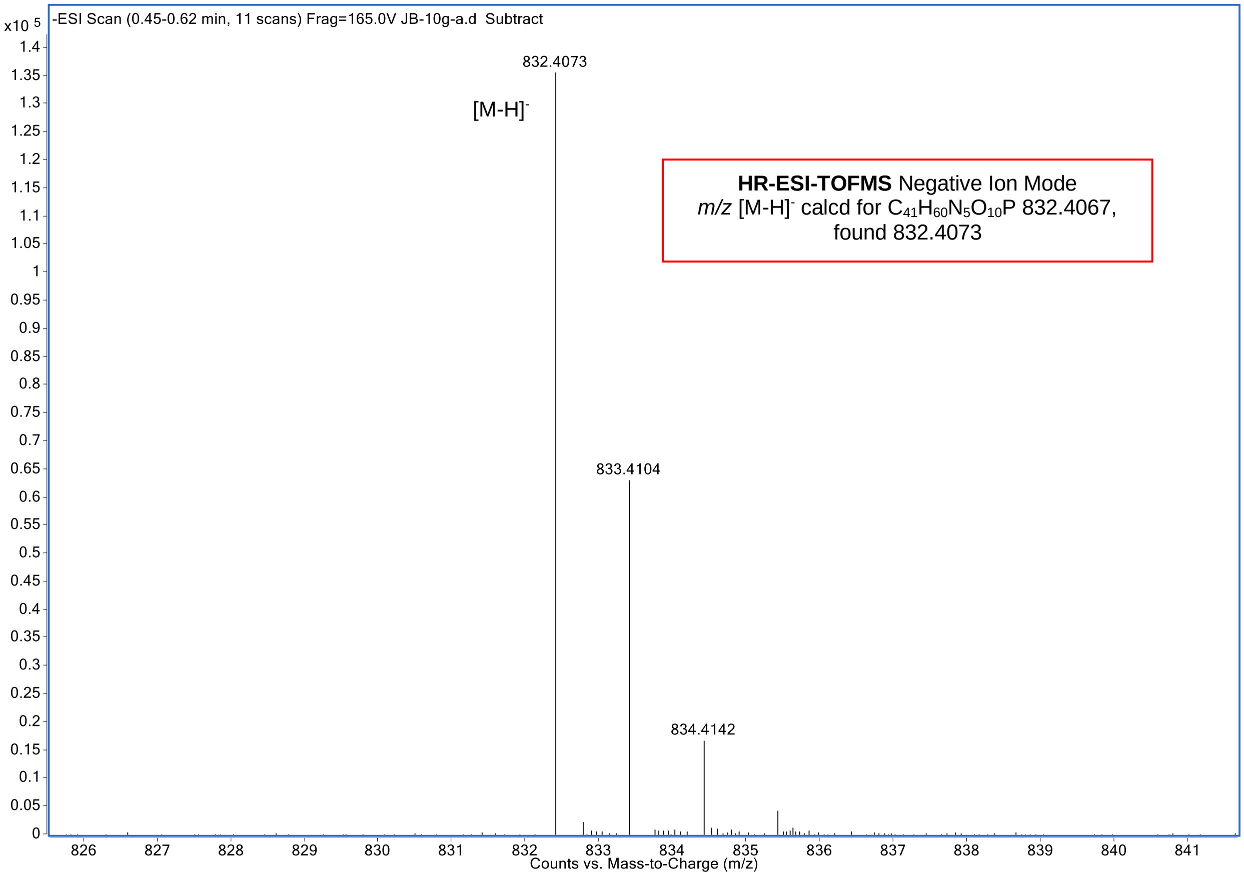

x10 5
-ESI Scan (0.45-0.62 min, 11 scans) Frag=165.0V JB-10g-a.d Subtract
832.4073
[M-H]-
833.4104
834.4142
HR-ESI-TOFMS Negative Ion Mode
m/z [M-H]- calcd for C41H60N5O10P 832.4067,
found 832.4073
Counts vs. Mass-to-Charge (m/z)

5. HPLC

Certificate of Analysis

Name of Compound: Phosphate B

Lot #: 861-79-24

Amount Shipped (Date): 0.362 g (06/17/2021)

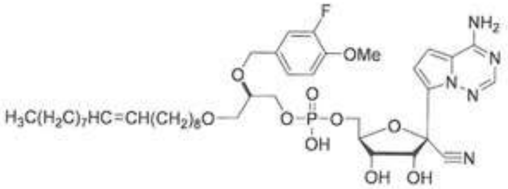

Physical Properties:

Molecular Weight:	833.94
Molecular Formula:	$C_{41}H_{61}FN_5O_{10}P$
Color, Form and Appearance:	off white solid
HPLC Purity:	> 99 area %
LCMS (ESI; $[M+H]^+$))	834.87
300 MHz ^1H NMR ($CDCl_3$ /MeOH-d4):	conforms

John Reichwein, Ph.D.
Associate Director Process Chemistry
Date: 06/17/2021

Compound 11a ((2*R*,3*S*,4*R*,5*R*)-5-(4-aminopyrrolo[2,1-*f*][1,2,4]triazin-7-yl)-5-cyano-3,4-dihydroxytetrahydrofuran-2-yl)methyl ((*R*)-2-(benzyloxy)-3-(hexadecyloxy)propyl) hydrogen phosphate

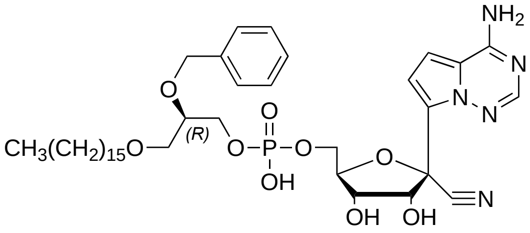

1. ^{1}H NMR

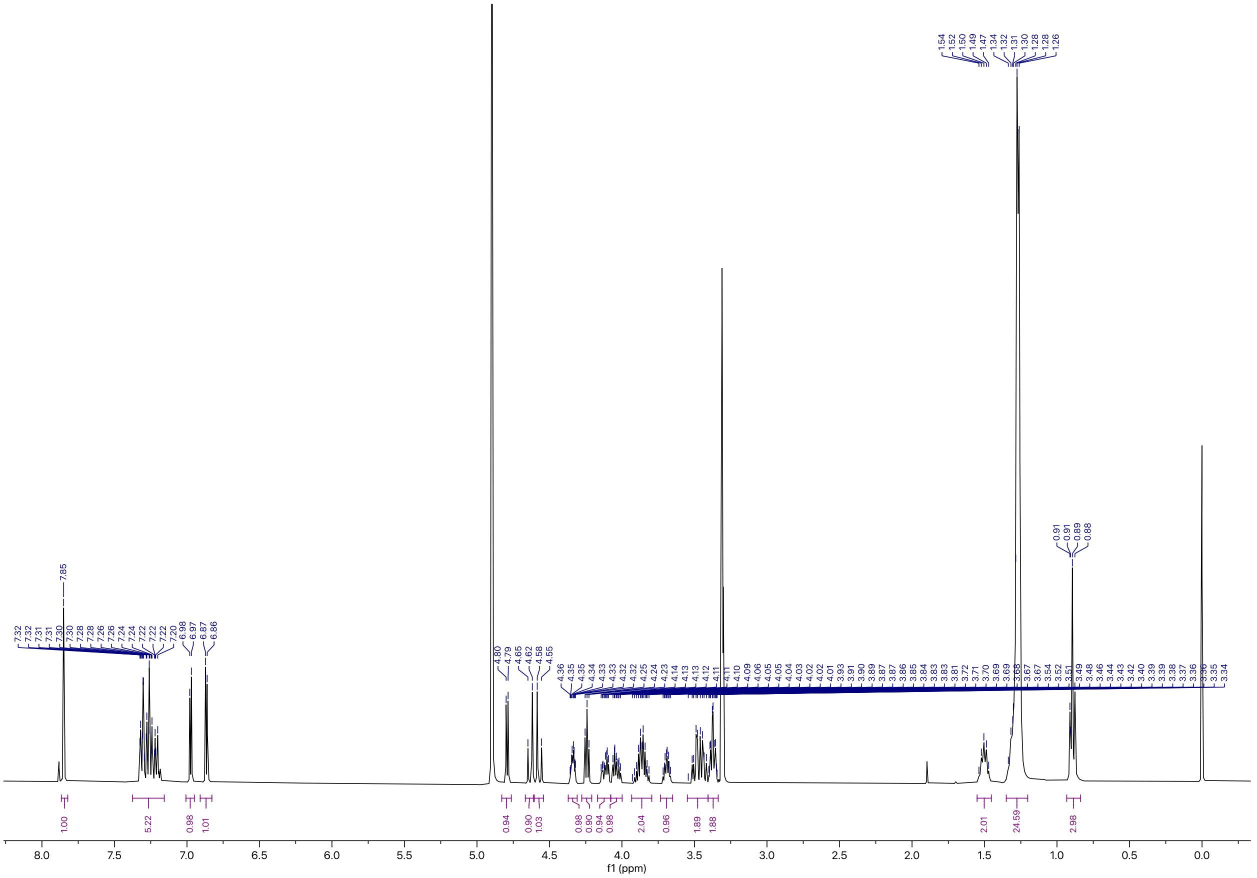

2. ^{13}C NMR

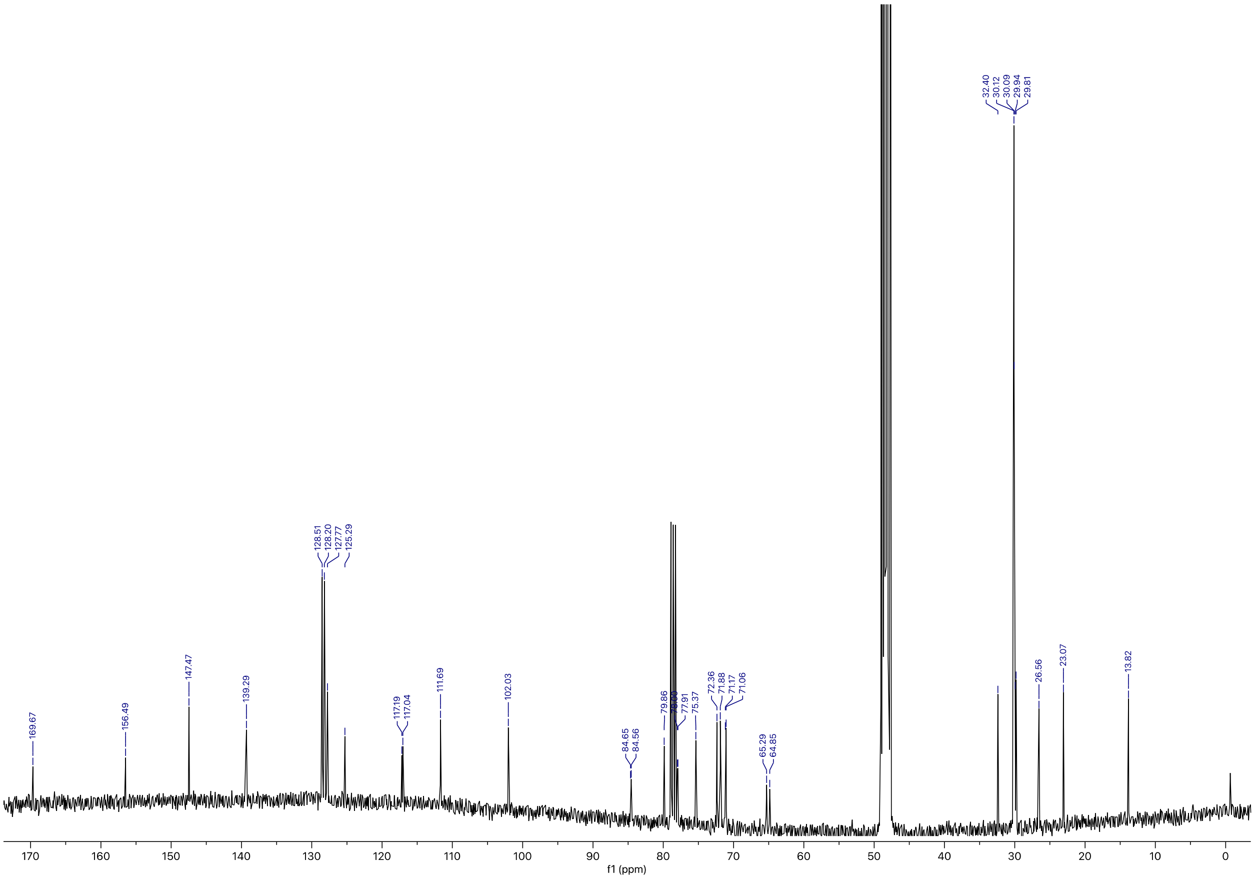

3. ^{31}P NMR

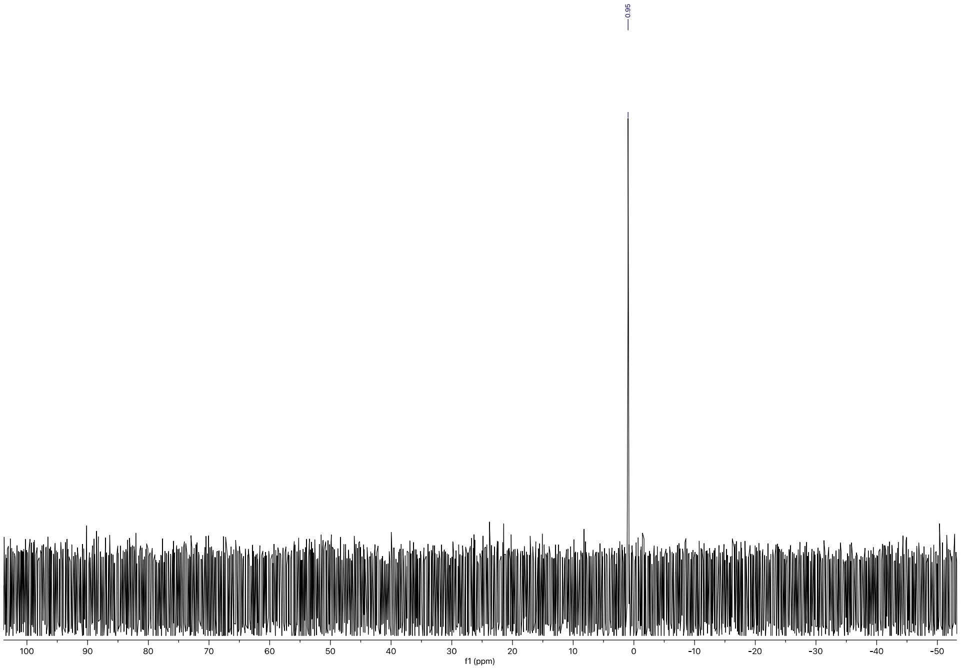

4. HRMS

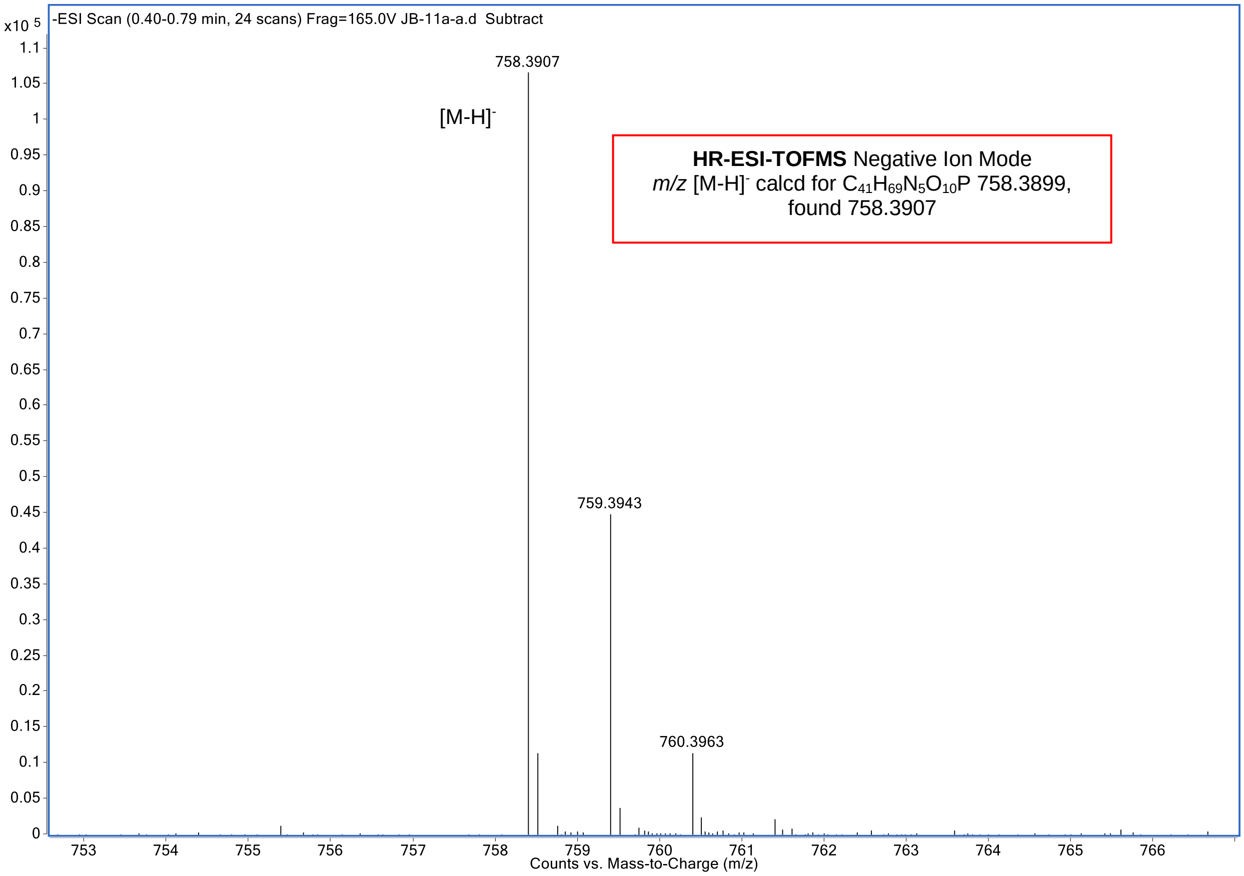

x10 5
-ESI Scan (0.40-0.79 min, 24 scans) Frag=165.0V JB-11a-a.d Subtract
758.3907
[M-H]-
HR-ESI-TOFMS Negative Ion Mode
m/z [M-H]- calcd for C41H69N5O10P 758.3899,
found 758.3907
759.3943
760.3963
Counts vs. Mass-to-Charge (m/z)

5. LCMS

ms filename	fmla Structure	Purity (%)	Weight (mg)	MW	NSID	Structure
094BP003	C38H58N5O9P	99.8	39.5	759.397	NSN24828-094BP003_LCMS	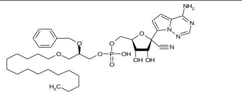

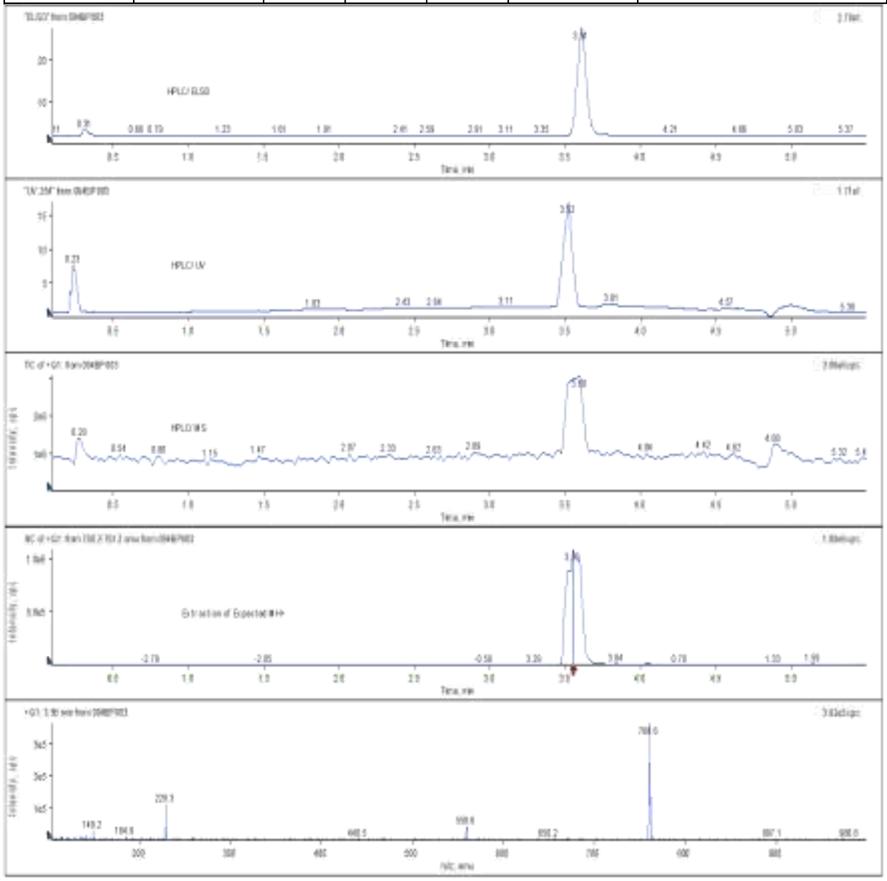

Compound 11g ((2*R*,3*S*,4*R*,5*R*)-5-(4-aminopyrrolo[2,1-*f*][1,2,4]triazin-7-yl)-5-cyano-3,4-dihydroxytetrahydrofuran-2-yl)methyl ((*R*)-2-((3-fluoro-4-methoxybenzyl)oxy)-3-(hexadecyloxy)propyl) hydrogen phosphate

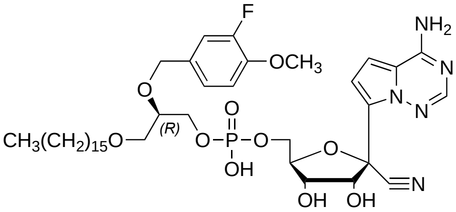

1. ^{1}H NMR

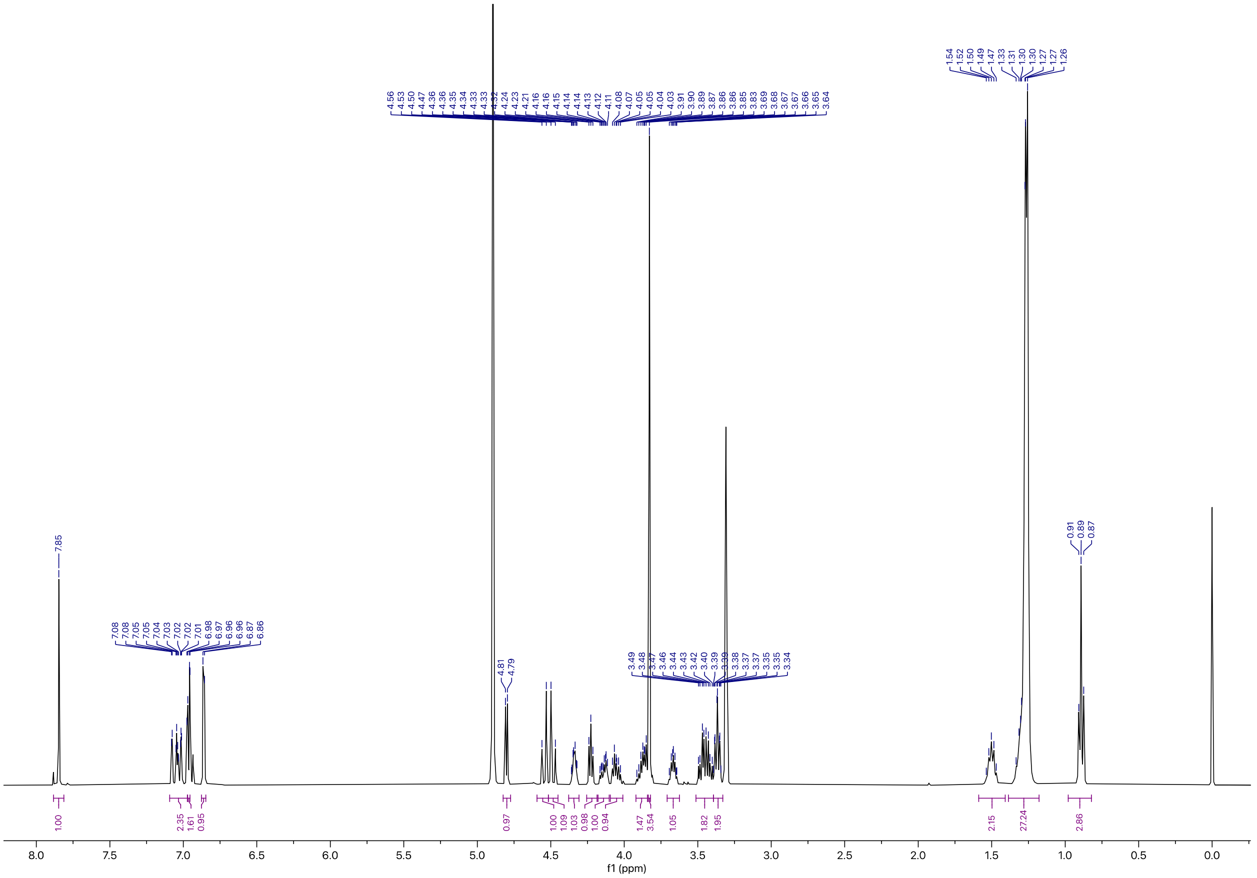

2. ^{13}C NMR

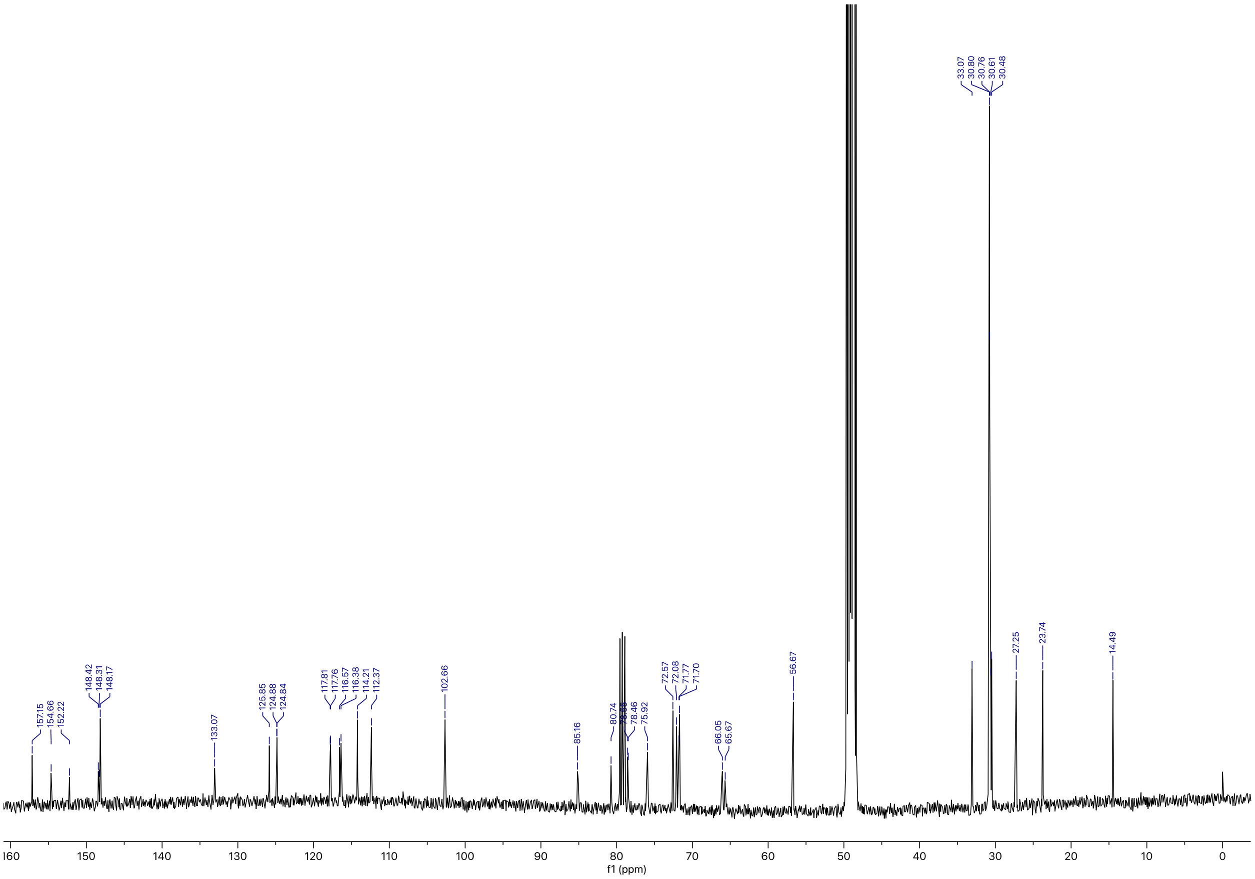

157.15
154.66
152.22
148.42
148.31
148.17
133.07
125.85
124.88
124.84
117.81
117.76
116.57
116.38
114.21
112.37
102.66
85.16
80.74
78.46
75.92
72.57
72.08
71.77
71.70
66.05
65.67
56.67
33.07
30.80
30.76
30.61
30.48
27.25
23.74
14.49
160
150
140
130
120
110
100
90
80
70
60
50
40
30
20
10
0
f1 (ppm)

3. HRMS

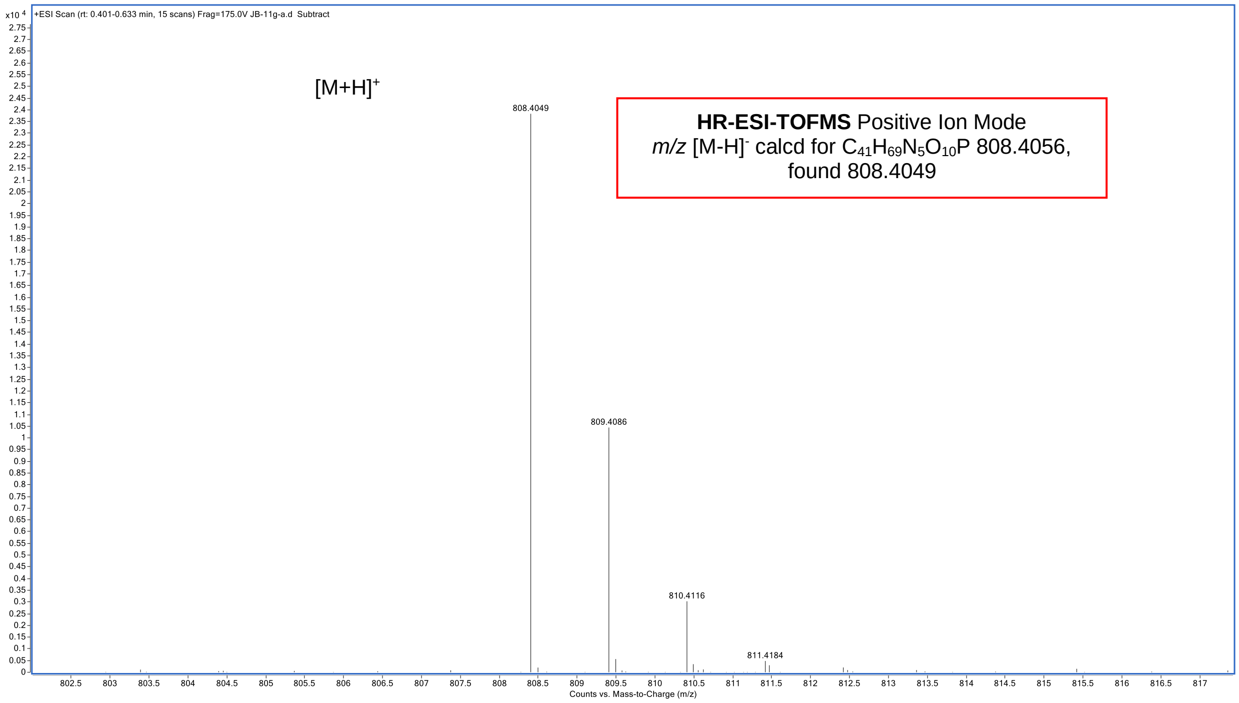

+ESI Scan (rt: 0.401-0.633 min, 15 scans) Frag=175.0V JB-11g-a.d Subtract
[M+H]+
808.4049
809.4086
810.4116
811.4184
HR-ESI-TOFMS Positive Ion Mode
m/z [M-H]- calcd for C41H69N5O10P 808.4056,
found 808.4049
Counts vs. Mass-to-Charge (m/z)

4. LCMS

ms filename	fmla Structure	Purity (%)	Weight (mg)	MW	NSID	Structure
094BP011	C39H59FN5O10P	99.8	294	807.398	NSN24829-094BP011_LCMS	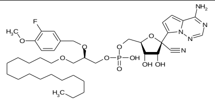

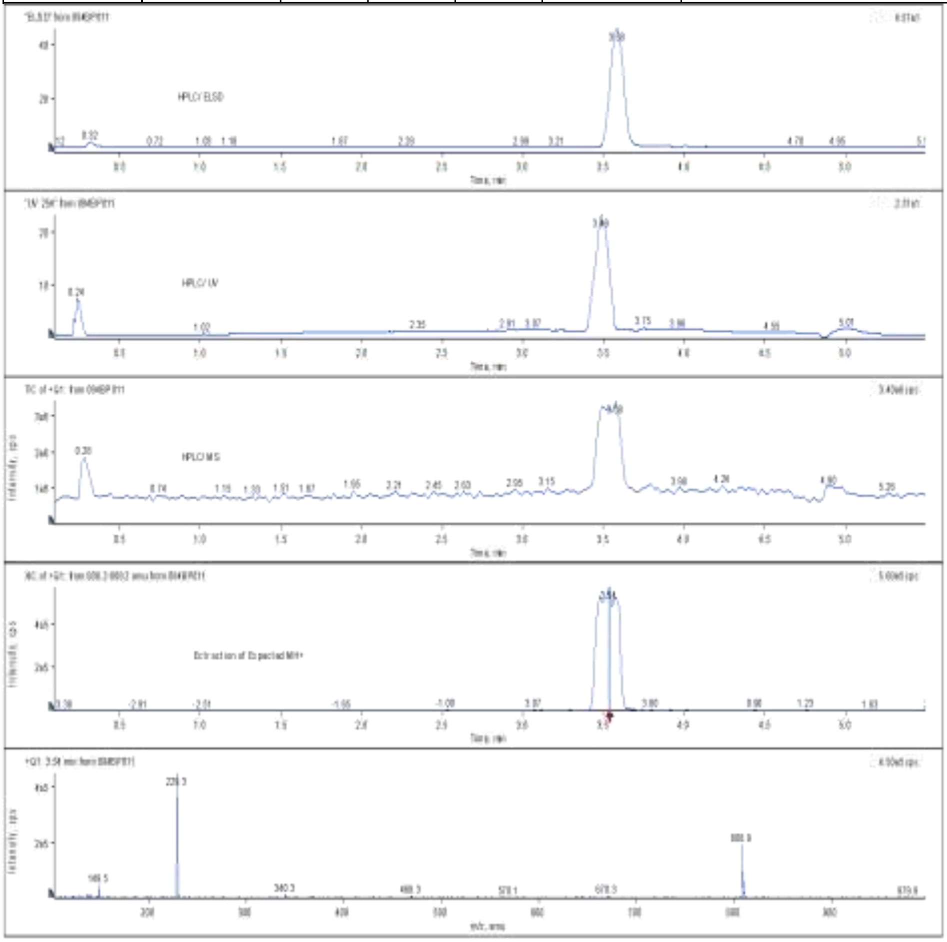

Compound 12a ((2*R*,3*S*,4*R*,5*R*)-5-(4-aminopyrrolo[2,1-*f*][1,2,4]triazin-7-yl)-5-cyano-3,4-dihydroxytetrahydrofuran-2-yl)methyl ((*R*)-2-(benzyloxy)-3-(tetradecyloxy)propyl) hydrogen phosphate

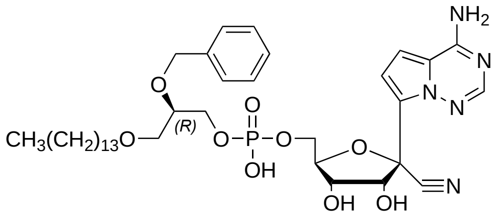

1. ^{1}H NMR

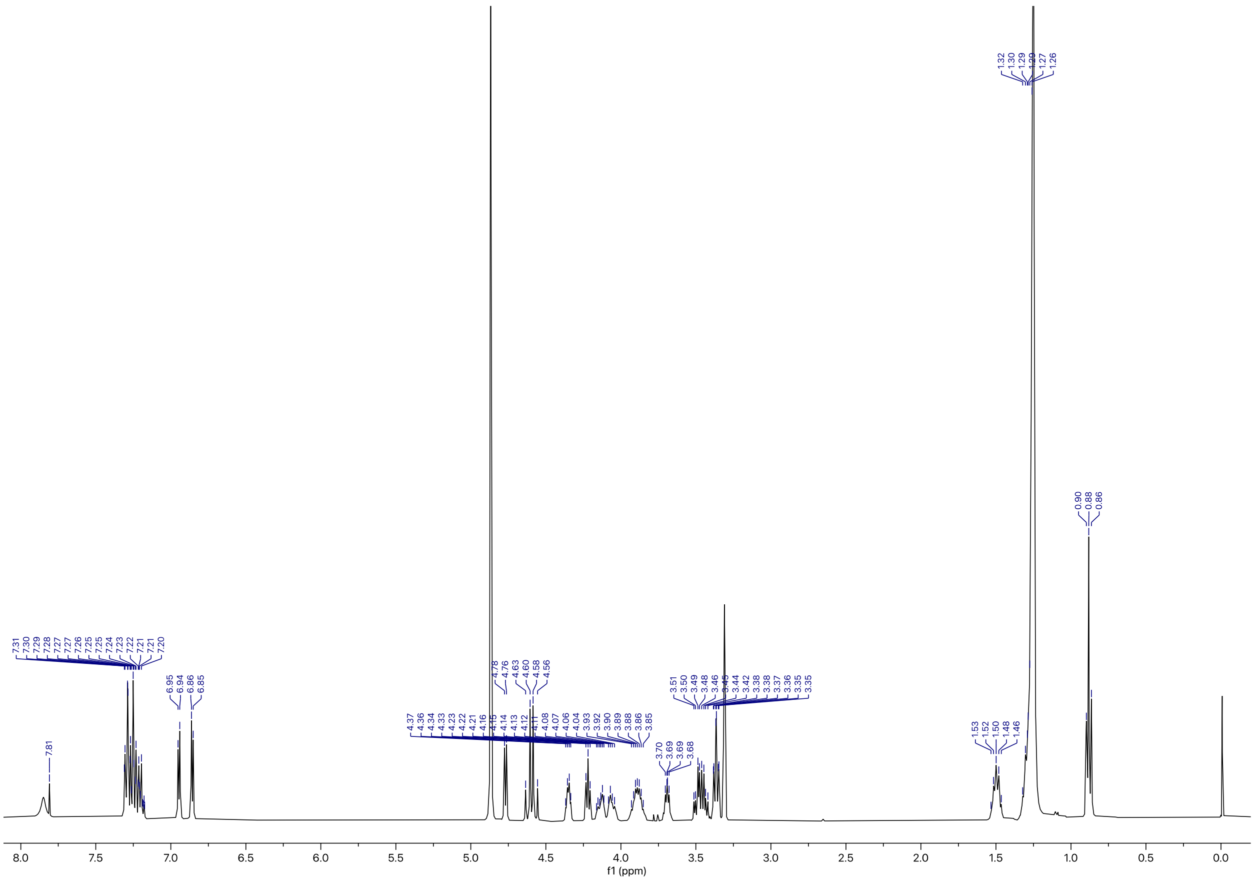

2. ^{13}C NMR

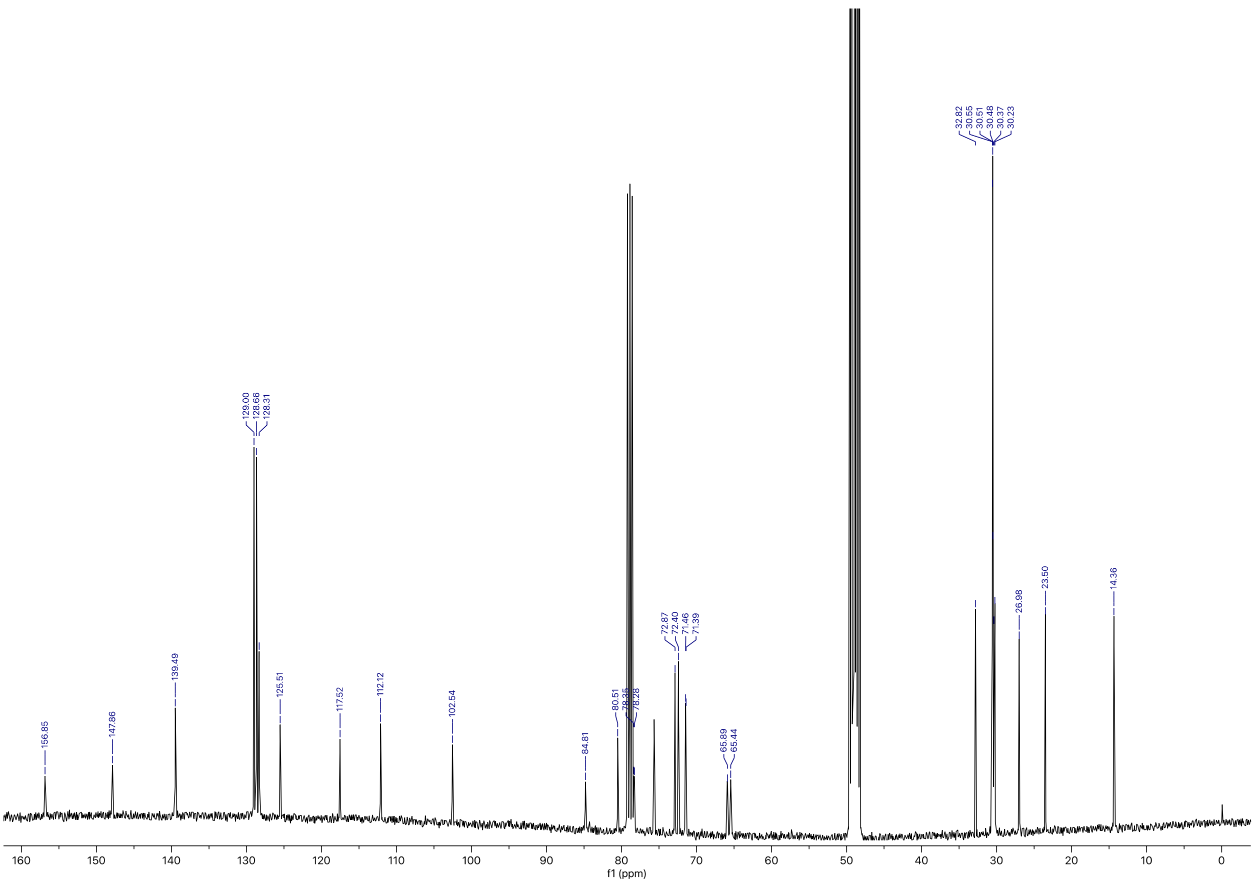

156.85
147.86
139.49
129.00
128.66
128.31
125.51
117.52
112.12
102.54
84.81
80.51
78.28
72.87
72.40
71.46
71.39
65.89
65.44
32.82
30.55
30.51
30.48
30.37
30.23
26.98
23.50
14.36
f1 (ppm)

3. HRMS

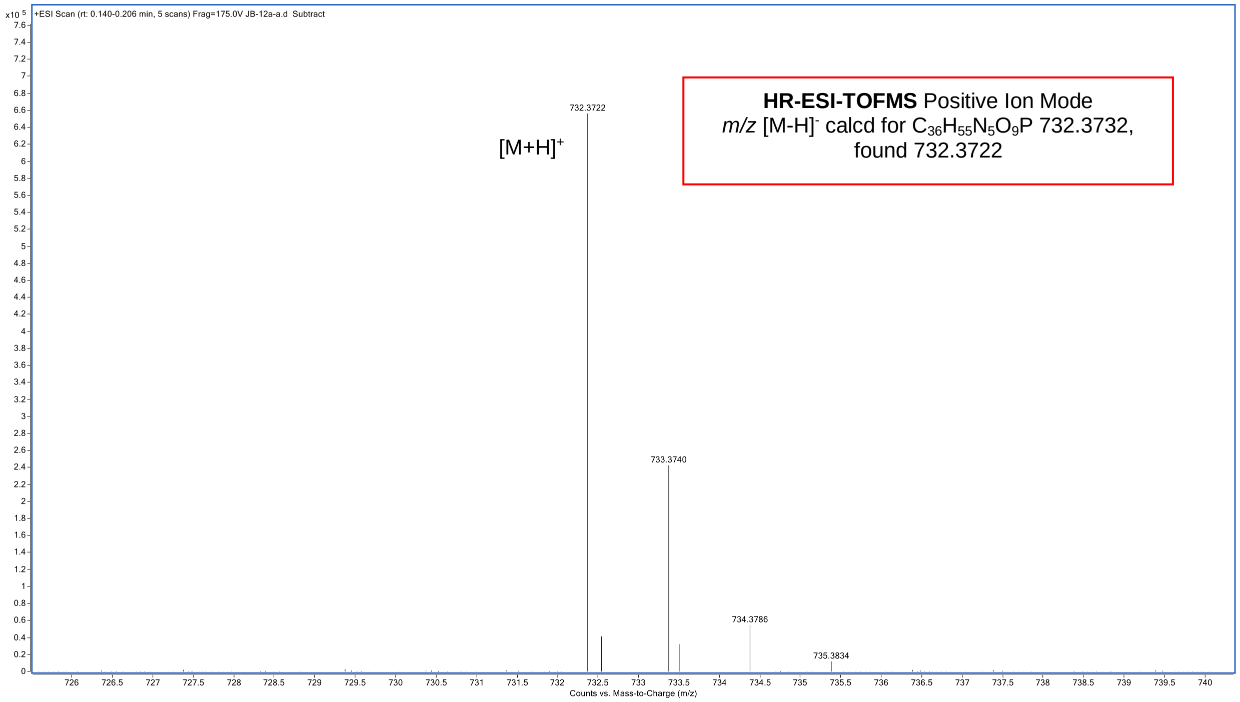

x10 5
+ESI Scan (rt: 0.140-0.206 min, 5 scans) Frag=175.0V JB-12a-a.d Subtract
732.3722
[M+H]+
733.3740
734.3786
735.3834
HR-ESI-TOFMS Positive Ion Mode
m/z [M-H]- calcd for C36H55N5O9P 732.3732,
found 732.3722
Counts vs. Mass-to-Charge (m/z)

4. HPLC

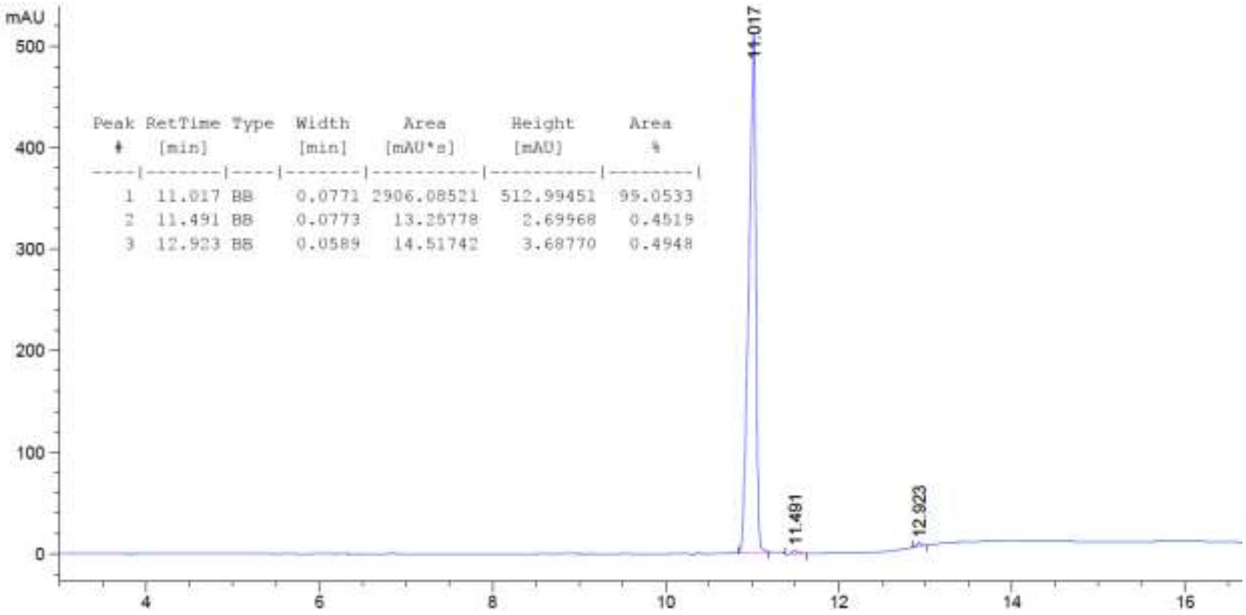

mAU
11.017
11.491
12.923
Peak # | RetTime [min] | Type | Width [min] | Area [mAU*s] | Height [mAU] | Area %
1 | 11.017 | BB | 0.0771 | 2906.08521 | 512.99451 | 99.0533
2 | 11.491 | BB | 0.0773 | 13.25778 | 2.69968 | 0.4519
3 | 12.923 | BB | 0.0589 | 14.51742 | 3.68770 | 0.4948

Figure S1. Relative cell viability in uninfected cells after treatment with V2043 and analogs

Calu-3 cells were treated for 44h (A), Huh7.5 cells were treated for 48h (B), and Vero-TMPRSS2 cells were treated for 38h (C) as in infection assays for each cell type. Cell viability was measured by CellTiter-Glo assay and reported as % cell viability relative to DMSO controls. Graphs show mean ± standard deviation of at least 3 (Calu-3 and Huh7.5) or 2 (Vero-TMPRSS2) independent experiments. Best fit curves calculated in Graphpad Prism 9 were used to compute cytotoxic concentration 50% (CC50) values in Tables 1-3.

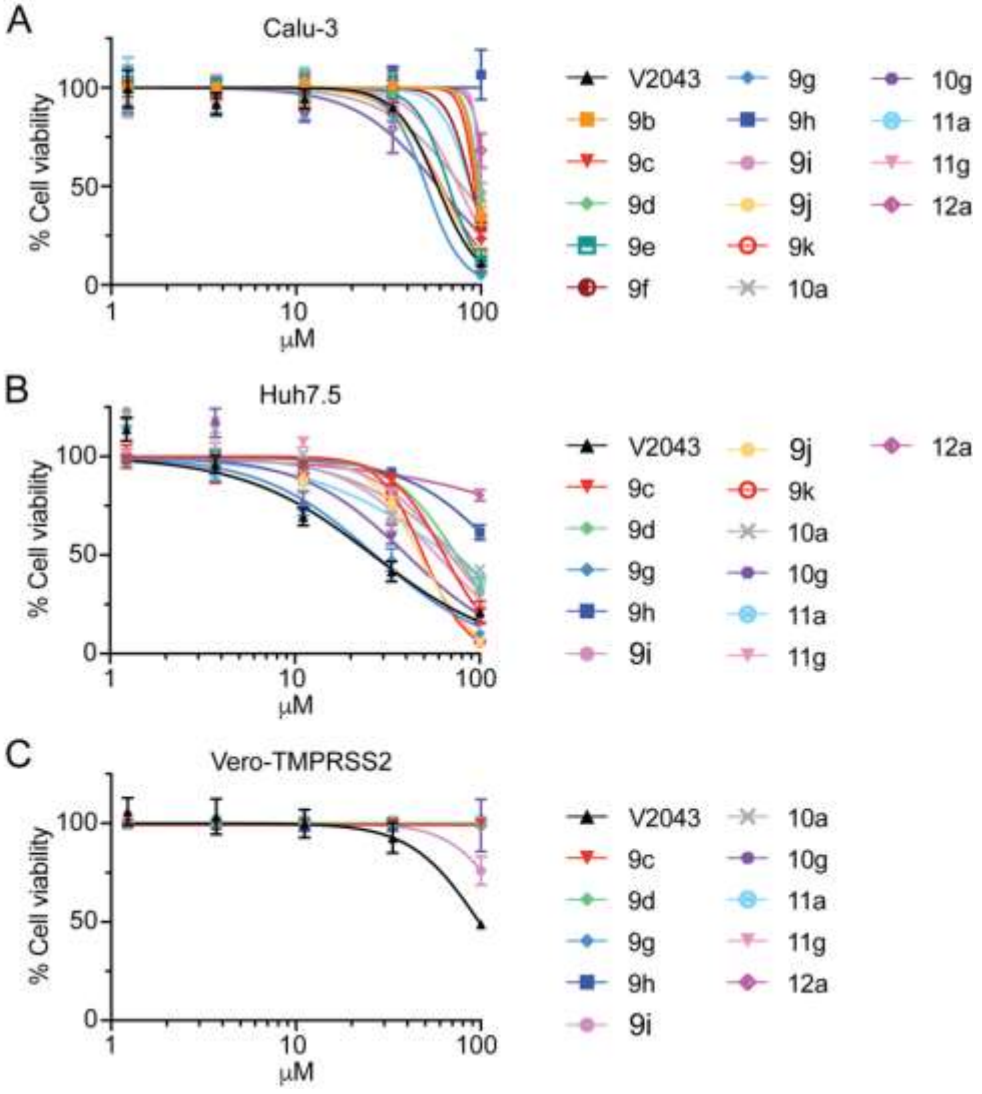

Table S1. Effect of V2043, GS-621763 or Molnupiravir given 12 or 24 hours postinfection on Lung Titers at Day 4 or 5 After SARS-CoV-2 Infection in Balb/c Mice

Compound	MW	Dose mg/kg	Total Dose μmoles/kg/day	Lung titer reduction log_{10} day 4	Lung titer reduction log_{10} day 5
				+12 hr / +24 hr	+12 hr / +24 hr
V2043	786	60 QD	76	-	>2.14/ >2.14
V2043	786	30 BID	76	-	>2.14/ >2.14
GS-621763*	501	30 BID	120	>2.99 / -	-
Molnupiravir*	329	30 BID	182	2.12 / -	-

Supplementary Table S1: Comparison of *in vivo* SARS-CoV-2 antiviral activity on a molar basis. *Data for GS-621763 and Molnupiravir were obtained from reference 13, Supplementary Data file S1 Schäfer, A et al. "Therapeutic treatment with an oral prodrug of the remdesivir parental nucleoside is protective against SARS-CoV-2 pathogenesis in mice". Abbreviations: BID – twice daily; QD - once daily; +12hr - 12 hours post-infection; +24hr - 24 hours post-infection; MW - molecular weight.

Comparing our data with the data of Schafer et al.,[13] V2043 is substantially more active on a molar basis than molnupiravir and has activity similar to GS-621763. The differences in molar dose levels are due to the variations in the size of the inactive prodrug moieties of the compounds.

(13) Schäfer, A.; Martinez, D. R.; Won, J. J.; Meganck, R. M.; Moreira, F. R.; Brown, A. J.; Gully, K. L.; Zweigart, M. R.; Conrad, W. S.; May, S. R.; Dong, S.; Kalla, R.; Chun, K.; Pont, V. D.; Babusis, D.; Tang, J.; Murakami, E.; Subramanian, R.; Barrett, K. T.; Bleier, B. J.; Bannister, R.; Feng, J. Y.; Bilello, J. P.; Cihlar, T.; Mackman, R. L.; Montgomery, S. A.; Baric, R. S.; Sheahan, T. P. Therapeutic treatment with an oral prodrug of the remdesivir parental nucleoside is protective against SARS-CoV-2 pathogenesis in mice. *Sci. Transl. Med.* **2022**, *14*, eabm3410.
